# PQQ‐dependent Dehydrogenase Enables One‐pot Bi‐enzymatic Enantio‐convergent Biocatalytic Amination of Racemic *sec*‐Allylic Alcohols

**DOI:** 10.1002/cctc.202001707

**Published:** 2020-12-22

**Authors:** Somayyeh Gandomkar, Raquel Rocha, Frieda A. Sorgenfrei, Lía Martínez Montero, Michael Fuchs, Wolfgang Kroutil

**Affiliations:** ^1^ Institute of Chemistry University of Graz, NAWI Graz 8010 Graz Austria; ^2^ Austrian Centre of Industrial Biotechnology c/o University of Graz 8010 Graz Austria; ^3^ Field of Excellence BioHealth University of Graz 8010 Graz Austria; ^4^ BioTechMed Graz 8010 Graz Austria

**Keywords:** Amination of alcohols, PQQ dependent dehydrogenase, *sec*-allylic alcohols, biocatalytic cascade, allylic amines, transaminases

## Abstract

The asymmetric amination of secondary racemic allylic alcohols bears several challenges like the reactivity of the bi‐functional substrate/product as well as of the α,β‐unsaturated ketone intermediate in an oxidation‐reductive amination sequence. Heading for a biocatalytic amination cascade with a minimal number of enzymes, an oxidation step was implemented relying on a single PQQ‐dependent dehydrogenase with low enantioselectivity. This enzyme allowed the oxidation of both enantiomers at the expense of iron(III) as oxidant. The stereoselective amination of the α,β‐unsaturated ketone intermediate was achieved with transaminases using 1‐phenylethylamine as formal reducing agent as well as nitrogen source. Choosing an appropriate transaminase, either the (*R*)‐ or (*S*)‐enantiomer was obtained in optically pure form (>98 % *ee*). The enantio‐convergent amination of the racemic allylic alcohols to one single allylic amine enantiomer was achieved in one pot in a sequential cascade.

## Graphical abstract

**Enantioconvergent amination**: Combining the chemoselective oxidation of racemic allylic secondary alcohols to the corresponding unsaturated ketone at the expense of Fe(III) as oxidant in the presence of a PQQ‐dependent enzyme with the formal reductive bio‐amination of the intermediate ketone opened a route to optically pure/enriched allylic amines.

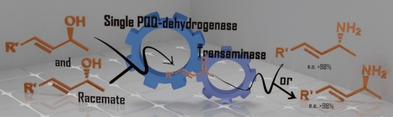

Chiral allylic amines are versatile building blocks in organic chemistry and have been widely used in the synthesis e. g. of pharmaceuticals.^[1]^ Although various methods for their preparation have been published,^[2]^ the direct metal catalyzed stereoselective amination of non‐activated allylic alcohols to unprotected primary allylic amines has not been reported, yet. One possible reason might be isomerization of the allylic alcohols in the presence of selected metal catalysts.^[3]^ Therefore, a concept for the stereoselective amination of allylic alcohols would expand the toolbox for their synthesis, ideally when racemic alcohols are used as starting material.

Biocatalytic methods have emerged as mild, sustainable alternatives for the synthesis of chiral amines using various classes of enzyme.^[4]^ For the amination of (racemic) *sec*‐alcohols (not allylic) artificial biocatalytic cascades^[5]^ have been reported by combining an alcohol‐dehydrogenase or oxidase catalyzed oxidation with asymmetric amination using transaminases^[6]^ or amine dehydrogenases (Scheme 1, top).^[7]^

**Scheme 1:**
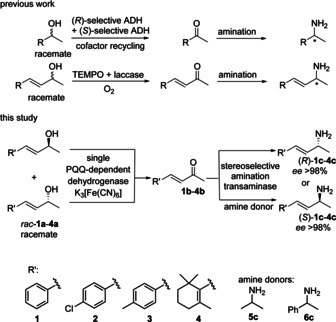
Stereoselective amination of racemic allylic *sec*‐alcohols. Previous amination cascades employed alcohol dehydrogenase (ADHs), mostly two to transform both enantiomers or used TEMPO as oxidant.

Allylic alcohols have only been investigated recently in a sequential two‐step chemoenzymatic approach^[8]^ by oxidizing the racemic alcohols using TEMPO in combination with a laccase followed by the reductive amination using transaminases yielding the chiral amines in moderate to good yields (29–75 %). Although here the non‐enantioselective chemical oxidation allows to transform both enantiomers, it requires a non‐natural and non‐chemoselective reagent (TEMPO) in combination with an oxidizing enzyme.

The ideal sequence would encompass a non‐enantioselective oxidation using a single (bio)catalyst followed by amination of the intermediate α,β‐unsaturated ketone employing also a single enzyme, thus requiring in total just two enzymes (Scheme 1, bottom). The bio‐amination of racemic *sec*‐alcohols in previous studies required at least two enzymes just for the oxidation step, namely for each enantiomer one,[^7g^, ^7h^, ^7i^] or in case where only one enzyme was used the conversion did not reach values >90 %.[^7h^, ^9^] Thus, a high yielding alcohol amination cascade with a single suitable active oxidative enzyme with low enantioselectivity applicable for a racemic substrate remains elusive.

Searching for a single enzyme capable of oxidizing both enantiomers of a racemate, we have previously reported on alcohol dehydrogenases (ADH)^[10]^ applicable for aliphatic *sec*‐alcohols and on oxidases for *sec*‐allylic alcohols.^[11]^ Also variants of an ADH from *Thermoanaerobacter ethanolicus* have been reported to display low enantioselectivity.^[12]^ Since ADHs require in general another enzyme for cofactor recycling, they were not considered here (the coupled substrate approach leads to chemoselectivity issues with the subsequent amination step). The O_2_‐dependent oxidases mentioned above showed low enantioselectivity, however the activity was not sufficient to enable also the oxidation of the slower reacting enantiomer within reasonable time and at a reasonable concentration of the enzyme. Since neither flavin‐dependent oxidases nor NAD(P)^+^‐dependent ADHs were available with satisfying properties, we turned our attention to a group of enzymes less investigated in biocatalysis, namely pyrroloquinoline quinone (PQQ) dependent dehydrogenases (PQQ‐DH). These enzymes have until now mainly been investigated from a structural or biochemical point of view.^[13]^ PQQ‐DHs can be used for instance with Fe(III) as the oxidant [e. g. in the form of Fe(CN)_6_^3−^], which additionally reduces the challenges of a gas‐liquid phase as present in the case of oxidases requiring molecular oxygen. Thus, for the PQQ‐DH dependent enzyme using Fe(III) as the oxidant, the oxidant as well as the enzyme are present in the same phase.

A PQQ‐dependent dehydrogenase identified in a detoxification sequence of deoxynivalenol (DON) at a substrate concentration of just 100 μM in the bacterial strain *Devosia mutans* (PQQ‐DH_*Dm*)^[14]^ attracted our attention. We investigated this enzyme for the oxidation of *sec*‐allylic alcohols **1** **a**–**4** **a** as model compounds and its potential for incorporation in a cascade for amination. Initially, the oxidation and amination step were tested separately, to check whether compatible conditions are feasible for the PQQ‐DH and a transaminase. A transaminase was considered as the enzyme of choice for the second step, since in contrast to other options (amine dehydrogenase) a single enzyme would be sufficient.

At an enzyme concentration of 16 μM, the purified PQQ‐DH_*Dm* enabled the quantitative oxidation of racemic substrate *rac*‐**1** **a**–**4** **a** at a substrate concentration of 10 mM in Tris‐HCl buffer (100 mM, pH 7.5) within only 0.5 hour at 21 °C (Table 1); thus, both enantiomers were readily oxidized to the corresponding ketone. This indicated that the PQQ‐DH_*Dm* may be indeed suitable for the desired purpose.

**Table 1:** Oxidation of racemic *sec*‐allylic alcohols using the PQQ‐dependent dehydrogenase from *Devosia mutans*.

Substr.	Conv._pure enzyme_^[a]^ [%]	App. Activity [U/mg]^[b]^	E^[c]^	Stereopref.^[d]^	Conv._*E. coli*/PQQ‐DH_^[e]^ [%]
*rac*‐**1** **a**	>99	1.6	19	(*S*)	90
*rac*‐**2** **a**	>99	0.9	22	(*S*)	98
rac‐**3** **a**	>99	1.4	16	(*S*)	95
*rac*‐**4** **a**	>99	1.8	41	(*S*)	98

[a] Reaction conditions: 1 mL volume, substrate (10 mM), purified PQQ‐DH (16 μM), Tris‐HCl buffer (100 mM, pH 7.5), PQQ (100 μM), potassium ferricyanide (20 mM), 0.5 hour, 170 rpm, 21 °C. The reaction was analyzed by GC‐FID. [b] Activity was deduced from the time course transforming the racemic substrate using 0.1 mg/mL of purified enzyme (see Table S4). Since the activity measured does not refer to a single enantiomer but to the racemate, it was termed apparent. [c] The enantioselectivity E was calculated from conv. and *ee* of the remaining substrate at a conv. ∼50 % (see Table S5) using the online tool http://biocatalysis.uni‐graz.at/biocatalysis‐tools/enantio. [d] Enantiomer which was oxidized faster. [e] Reaction conditions as in [a] but lyophilized *E. coli*/PQQ‐DH cells (20 mg/mL), 24 h.

Measuring the activity, the apparent value for the racemate varied between 0.9 and 1.8 U/mg depending on the substrate, whereby *rac*‐**4** **a** was oxidized fastest (1.8 U/mg). Since for applications the use of crude enzyme preparations or even permeabilized *E. coli* cells containing the overexpressed enzyme is preferred over purified enzyme, the oxidation was re‐investigated using lyophilized *E. coli*/PQQ‐DH_*Dm* cells. Luckily, it turned out, that also this crude preparation can be used, whereby conversion between 90–98 % were reached within 24 hours at a substrate concentration of 10 mM. To get an idea of the enantioselectivity E of the enzyme for the two enantiomers, thus, what is the ratio of the rate constants of the oxidation for the two enantiomers (k_S_/k_R_), the E‐value was determined from the *ee* of the remaining alcohol and the conversion. As expected, the E‐value was low, varying between 16 and 41. For all substrates investigated preferentially the (*S*)‐enantiomer was oxidized. Thus, the here investigated PQQ‐DH_*Dm* displayed the same stereopreference as O_2_‐dependent oxidases previously reported (e. g. HMFO).^[11]^

The organic solvent tolerance of the PQQ‐DH was rather encouraging, since for instance the enzyme was active in DMSO up to 30 % v/v (Table S6). Applying an aqueous two‐phase system, isooctane and *n*‐heptane can be used at up to 50 % v/v.

Turning to the amination step, the biocatalytic asymmetric synthesis of allylic amines from the corresponding α,β‐unsaturated ketones has only been reported using commercial enzymes with undisclosed amino acid sequence whereby e. g. optically active (3*E*)‐4‐phenylbut‐3‐en‐2‐amine was obtained with low conversion (<30 %)^[15]^ or with conversion up to 70 %.^[8]^

Especially the amination of an unsaturated ketone may be prone to undesired side products considering that the amination reagent such as e. g. 2‐propylamine or 1‐phenylethylamine may also undergo a Michael‐addition with the α,β‐unsaturated ketones. This side reaction may occur additionally to the expected hemiaminal and Schiff base formation between amines and ketones present in the reaction mixture.

Testing various (*R*)‐selective enzymes for the stereoselective amination of **1** **b**–**4** **b**, ArRmut11 ω‐TA^[16]^ was identified as the best suitable candidate (Table 2, see also Supporting Information section 2.2).

**Table 2:** Stereoselective amination of α,β‐unsaturated ketones **1** **b**–**4** **b**.^[a]^

Substr.	Transaminase	Amine donor	Donor conc. [M]	Conv. [%]	*ee* [%]^[b]^
**1** **b**	ArRmut11	(*R*)‐**6** **c**	1.25	93^[c]^	>98 (*R*)
**1** **b**	*S. pomeroyi*	(*S*)‐**6** **c**	0.3	97^[d]^	>98 (*S*)
**2** **b**	ArRmut11	(*R*)‐**6** **c**	1.25	96^[c]^	>98 (*R*)
**2** **b**	*S. pomeroyi*	(*S*)‐**6** **c**	0.3	96^[d]^	>98 (*S*)
**3** **b**	ArRmut11	(*R*)‐**6** **c**	1.25	78^[c]^	>98 (*R*)
**3** **b**	*S. pomeroyi*	(*S*)‐**6** **c**	0.3	76^[d]^	>98 (*S*)
**4** **b**	ArRmut11	(*R*)‐**6** **c**	1.25	55^[c]^	>98 (*R*)
**4** **b**	*S. pomeroyi*	(*S*)‐**6** **c**	0.3	24^[d]^	>98 (*S*)

[a] For a complete overview of all transaminases and conditions tested see Supporting Information. [b] The *ee* was measured by HPLC on a chiral phase after acetylation. [c] Reaction condition: 1 mL reaction volume, KPi (200 mM, pH 8.0), substrate (50 mM), 10 % v/v DMSO, amine donor, PLP (1 mM), lyophilized cells (40 mg/mL) in 2 mL tubes, 16 h, 450 rpm, 40 °C. Conv. was measured by GC‐FID. [d] Reaction conditions as in [c] but 1.5 mL volume, substrate (10 mM), 4 mL glass vials, 24 h, 350 rpm, 30 °C. The conversion reported is based on calibration.

Out of three amine donors tested [2‐propylamine (**5** **c**), (*R*)‐1‐phenylethylamine (*R*)‐**6** **c** and *all*‐*rac*‐1,2‐diaminocyclohexane], (*R*)‐1‐phenylethylamine (*R*)‐**6** **c** led to the highest conversion at 1.25 M compared to the other amine donors investigated (Table 2, Figures S3–S6). In all cases, optically pure (*R*)‐allylic amines (*R*)‐**1** **c**–**4** **c** were obtained (>98 % *ee*).

To access the (*S*)‐enantiomer, the transaminase originating from *Silicibacter pomeroyi* ^[17]^ was identified best (Tables S10‐S14) using ketone **1** **b** as the model substrate. Ketone **1** **b** was aminated with 97 % conversion leading to optically pure (*S*)‐**1** **c** (>98 % *ee*) (Table 2). This transaminase was subsequently also used for the asymmetric amination of the other α,β‐unsaturated ketones providing in all cases optically pure (*S*)‐amine (*S*)‐**2** **c**–**4** **c**. Thus, depending on the TA chosen, the (*R*)‐ as well as the (*S*)‐allylic amines **1** **c**–**4** **c** were accessible in optically pure form.

Turning now to the cascade to transform the racemic *sec*‐allylic alcohols to optically pure amines, the oxidation and amination steps were started simultaneously in one pot; however, this led only to low amounts of amine formation (2–6 %), which was mainly attributed to an incompatibility of the PQQ‐DH with the amine donor of the second step. Consequently, the cascade was performed in one pot in a sequential fashion using the optimal conditions for each step; thus, after the oxidation at pH 7.0, the transaminase, PLP and the amine donor were added and the pH was adjusted to pH 8.0 and the amination was run for 24 hours. Performing the first step at the same pH as the amination (pH 8.0) led to less amine formation.

The results of the cascade showed that the asymmetric (*R*)‐amination of racemic alcohols *rac*‐**1** **a**–**3** **a** went to completion giving optically pure amines (*R*)‐**1** **c**–**3** **c** (>98 % *ee*) (Table 3).

**Table 3:** Bi‐enzymatic (*R*)‐ and (*S*)‐stereoselective amination of racemic allylic alcohols *rac*‐**1** **a**–**4** **a** using a PQQ‐DH and a (*R*)‐ or (*S*)‐transaminase (TA).

Substr.	Transaminase	Amine Donor	Conv.^[b]^ [%]	*ee* [%]^[c]^
*rac*‐**1** **a**	ArRmut11	(*R*)‐**6** **c**	>99	>98 (*R*)
*rac*‐**1** **a**	*S. pomeroyi*	(*S*)‐**6** **c**	99	>98 (*S*)
*rac*‐**2** **a**	ArRmut11	(*R*)‐**6** **c**	>99	>98 (*R*)
*rac*‐**2** **a**	*S. pomeroyi*	(*S*)‐**6** **c**	96	>98 (*S*)
*rac*‐**3** **a**	ArRmut11	(*R*)‐**6** **c**	>99	>98 (*R*)
*rac*‐**3** **a**	*S. pomeroyi*	(*S*)‐**6** **c**	92	>98 (*S*)
*rac*‐**4** **a**	ArRmut11	(*R*)‐**6** **c**	57	>98 (*R*)
*rac*‐**4** **a**	*S. pomeroyi*	(*S*)‐**6** **c**	66	>98 (*S*)

[a] Reaction condition: substrate (10 mM), KPi (200 mM, pH 7.0 for oxidation step, 8.0 for amination step) DMSO (2 % v/v), PQQ‐DH (20 mg/mL), PQQ (100 μM), potassium ferricyanide (20 mM) for oxidation step, TA (40 mg/mL), 1‐phenylethylamine [(*R*)‐**6** **c**, 250 mM or (*S*)‐**6** **c**, 300 mM], PLP (1 mM) for amination step. In 4 mL glass vial, final reaction volume: 1.5 mL. The oxidation was run at 170 rpm, horizontal shaking, 21 °C for 48 h, after addition of TA and amine donor, samples were incubated at 350 rpm, horizontal shaking, 24 h, 40 °C for ArRmut11 or 30 °C for *S. pomeroyi*. [b] The conversion reported is based on calibration. [c] The *ee* was analyzed on HPLC on a chiral phase after acetylation.

For *rac*‐β‐ionol (*rac*‐**4** **a**) 57 % of amine were obtained. In the case of asymmetric (*S*)‐amination, the amount of amine for the racemic alcohols *rac*‐**1** **a**–**3** **a** was always above 90 % (91–98 %). For all cascade reactions, the conversion of the oxidation step was analyzed separately, and it turned out, that the oxidation has gone to completion prior the addition of the amination agents.

For the three best converted racemic alcohols *rac*‐**1** **a**–**3** **a**, the bi‐enzymatic (*R*)‐amination cascade was also performed on a 0.2 mmol scale to get sufficient amounts (30 %, 25 % and 41 % isolated yields for **1** **c**, **2** **c** and **3** **c**, respectively) to measure the optical rotation and confirm and support the absolute configuration (Table S18). Due to the still rather high price of PQQ, larger scale experiments were not performed.

In conclusion, the enantioconvergent biocatalytic sequential cascade enables the transformation of racemic allylic alcohols to one single enantiomer of the corresponding primary amine requiring just two enzymes in an oxidation‐reduction sequence. Although allylic alcohols and also the intermediate α,β‐unsaturated ketone are bi‐functionalized compounds prone to side reactions, this cascade run with high chemoselectivity without detecting any side products under the conditions employed. The oxidation was achieved by a PQQ‐dependent dehydrogenase displaying low enantioselectivity at the expense of iron(III) as the oxidant thereby circumventing the need for cofactor recycling as well as challenges to handle a gas such as molecular oxygen. For the formal reductive amination of the carbonyl functionality of the α,β‐unsaturated ketone defined enzymes were reported for the first time leading to the optically pure (*R*)‐ or (*S*)‐amine enantiomer. The results show, that PQQ‐dependent dehydrogenases relying on Fe(III) are highly interesting and useful enzymes for the oxidation of secondary allylic alcohols and can be incorporated in challenging sequential cascade reactions e. g. to prepare optically pure allylic amines paving the way to a new chapter for synthetic applications.

## Conflict of interest

The authors declare no conflict of interest.

## Supporting information

As a service to our authors and readers, this journal provides supporting information supplied by the authors. Such materials are peer reviewed and may be re‐organized for online delivery, but are not copy‐edited or typeset. Technical support issues arising from supporting information (other than missing files) should be addressed to the authors.

Supplementary
