# Supplementary material for: PQQ‐dependent Dehydrogenase Enables One‐pot Bi‐enzymatic Enantio‐convergent Biocatalytic Amination of Racemic sec‐Allylic Alcohols
Source: ChemCatChem. 2020 Dec 22;13(5):1290–3. doi: 10.1002/cctc.202001707 (PMC7986696; doi:10.1002/cctc.202001707)
Supplement: Supplementary file 1 — Supplementary [file CCTC-13-1290-s001.pdf]

ChemCatChem

Supporting Information

PQQ-dependent Dehydrogenase Enables One-pot Bi-enzymatic Enantio-convergent Biocatalytic Amination of Racemic *sec*-Allylic Alcohols

Somayyeh Gandomkar, Raquel Rocha, Frieda A. Sorgenfrei, Lía Martínez Montero, Michael Fuchs, and Wolfgang Kroutil*

Table of Contents

1 Material and methods 3

1.1. Chemicals 3

1.2. Synthesis 3

1.2.1. Synthesis of allylic alcohols from their corresponding ketones 3

1.2.2. NMR of the allylic alcohols

1.2.3. Synthesis of allylic amines from their corresponding ketones 4

1.2.4. NMR of allylic oximes and amines 7

1.2.5. NMR of isolated allylic amines from upscaling 9

1.3. Biocatalytic reactions 9

1.3.1. Preparation of the biocatalysts 9

1.3.2. General protocols for the biotransformations 12

1.4. Absolute configuration determination 17

2 Results 17

2.1. Oxidation step using PQQ-DH 17

2.2. Amination step using various ω-TAs and amine donors 21

2.2.1. Amination of **1b-4b** using ArRmut11-ωTA and various amine donors 21

2.2.2. Amination of **1b-4b** using ArRmut11-ωTA with (*R*)-1-phenyl-ethylamine as the amine donor at different pH level 24

2.2.3. Amination of **1b** using various ω-TAs 25

2.2.4. Enantiomeric excess measurements 27

2.3. One-pot two-step cascade 28

2.4. Amination cascade on 0.2 mmol scale 30

3 Analytics 30

4 Supplementary 35

4.1. NMRs 35

4.2. NMRs of purified amines from 0.2 mmol scale 45

4.3. HPLC chromatograms 47

4.4. GC chromatograms 57

4.5. GC-MS chromatograms 63

4.6. GC-MS calibration 71

4.7. GC-FID calibration 74

5 References 77

1. Material and methods

1.1. Chemicals

Reagents and organic solvents were obtained from commercial suppliers in reagent grade quality and used without further purification unless otherwise stated.

1.2. Synthesis

1.2.1. Synthesis of allylic alcohols from their corresponding ketones

Substrates **1a-3a** were synthesized from their corresponding ketones. For that purpose, to a solution of various ketones in methanol (30 mL), sodium borohydride was slowly added on ice (details in Table S1). The reaction mixture was stirred for 2 hours and the formation of the product was monitored by TLC. When the reaction was completed, quenching was done by using saturated aqueous NH_4Cl (15 mL). Then the resultant mixture was concentrated under reduced pressure and the residue was extracted with ethyl acetate (3 x 20 mL). The combined organic fractions were washed with brine, dried using Na_2SO_4 and concentrated under reduced pressure. Purification of the residue was done by flash chromatography (8:2, Cyclohex:EtOAc). The yields after the purification are reported in Table S1.

Table S1. Details of the reduction reactions

Sub.	Starting allylic ketones	$NaBH_4$	Yield
**1b**	MW: 146.07<br>2 g (14 mmol)	0.53 g (13 mmol)	2.16 g (quant.)
**2b**	MW: 180.03<br>2 g (11 mmol)	0.42 g (11 mmol)	2.13 g (quant.)
**3b**	MW: 160.22<br>2 g (13 mmol)	0.47 g (13 mmol)	1.74 g (87%)

1.2.2. NMR of the allylic alcohols

a) (3*E*)-4-phenylbut-3-en-2-ol (1a)

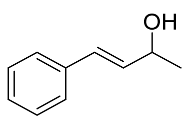

2.16 g (quant. yield, pale yellow solid, mp 32-34 ºC), ^{1}H NMR (300 MHz, $CDCl_3$): δ 7.52 – 7.21 (5H, m, C_6**H_5**), 6.60 (1H, dd, *J* = 16.0, 0.8 Hz, C_6H_5**H**C=), 6.30 (1H, dd, *J* = 15.9, 6.3 Hz, =C**H**CHOH), 4.51 (1H, pd, *J* = 6.4, 1.1 Hz, C**H**(OH)), 2.51 (1H, br s, O**H**), 1.41 (3H, d, *J* = 6.4 Hz, C**H_3**); ^{13}C NMR (75 MHz, $CDCl_3$): δ 136.8, 133.7, 129.3, 128.6, 127.6, 126.5, 68.8, 23.5.[1]

b) (3*E*)-4-(4-chlorophenyl)but-3-en-2-ol (2a)

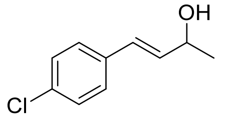

2.13 g (quant. yield), ^{1}H NMR (300 MHz, $CDCl_3$): 7.34 – 7.28 (4H, m, Ar), 6.55 (1H, dd, *J* =15.9, 1.0 Hz, Ar**H**C=), 6.26 (1H, dd, *J* =15.9, 6.2 Hz, =C**H**CHOH), 4.55 – 4.47 (1H, m, C**H**(OH)), 1.61 (1H, s, OH), 1.39 (1H, d, *J* =6.4 Hz, C**H_3**); ^{13}C NMR (75 MHz, $CDCl_3$) δ 135.2, 134.2, 133.2, 128.7, 128.1, 127.6, 68.8, 23.4.[2]

c) (3*E*)-4-(*p*-tolyl)but-3-en-2-ol (3a)

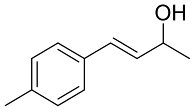

1.74 g (87% yield, pale yellow solid, mp 39-42 ºC), ^{1}H NMR (300 MHz, $CDCl_3$): δ 7.38 (2H, d, *J* = 8.1 Hz, Ar), 7.22 (2H, d, *J* = 8.0 Hz, Ar), 6.63 (1H, d, *J* =15.9 Hz, Ar**H**C=), 6.34 (1H, dd, *J* =15.9, 6.3 Hz, =C**H**CHOH), 4.58 (1H, p, *J* = 6.3 Hz, C**H**(OH)), 3.90 (1H, s, OH), 2.46 (3H, s, C**H$_3$**), 1.50 (3H, d, *J* =6.4 Hz, HOCHC**H$_3$**); ^{13}C NMR (75 MHz, $CDCl_3$) δ 137.3, 134.3, 133.0, 129.4, 129.1, 126.6, 68.8, 23.6, 21.3.[2]

1.2.3. Synthesis of allylic amines from their corresponding ketones

The reference racemic allylic amines including (3*E*)-4-phenylbut-3-en-2-amine (**1c**), (3*E*)-4-(4-chlorophenyl)but-3-en-2-amine (**2c**), (3*E*)-4-(*p*-tolyl)but-3-en-2-amine (**3c**) and (3*E*)-4-(2,6,6-trimethylcyclohex-1-en-1-yl)but-3-en-2-amine (**4c**) were synthesized by following procedures.

a) Preparation of (3*E*)-4-phenylbut-3-en-2-amine (1c)

Synthesis of (E)-4-phenylbut-3-en-2-one oxime

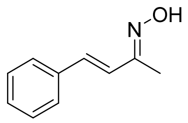

To a solution of (3*E*)-4-phenylbut-3-en-2-one (5 g, 34.2 mmol) and $NH_2OH{\cdot}HCl$ (5 g, 72 mmol) in ethanol (30 mL, 80% v/v), NaOH (8 g, 200 mmol) was slowly added at room temperature. The reaction mixture was heated to 80 °C and refluxed overnight. The reaction was monitored by TLC (7:3 cyclohexane:EtOAc). After completion, the reaction was subsequently quenched by adding saturated aqueous NH_4Cl (150 mL) and 2 N HCl (15 mL, pH ~ 7). The reaction mixture was extracted with CH_2Cl_2 (3 x 100 mL). The combined organic fractions were washed with brine (100 mL), dried with Na_2SO_4 and concentrated under reduced pressure yielding a yellowish solid (4.95 g, ~90%, R_f = 0.54, 7:3 cyclohexane:EtOAc). The NMR (^{1}H and ^{13}C in $CDCl_3$) data were in accordance with the literature.[3] The product of this step was used directly in the second step without further purification.

Synthesis (3E)-4-phenylbut-3-en-2-amine (1c)

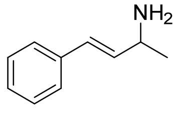

To an ice-cooled solution of (3*E*)-4-phenylbut-3-en-2-one oxime (2.52 g, 16 mmol) in ethanol (20 mL) and acetic acid (20 mL), zinc dust (10 g, 153 mmol) was added slowly. The reaction mixture was stirred for 10 min on ice, subsequently warmed to room temperature and refluxed at 50 °C for 1 h. The formation of the product was monitored by TLC (7:3 cyclohexane:EtOAc). The zinc dust was filtered *via* celite (celite 500 fine) and washed with a generous amount of ethanol (200 mL). The filtrate was evaporated under reduced pressure until a thick and dark yellowish liquid was obtained. The residue was dissolved in distilled water (50 mL) generating a pH value of ~6-7, then 2 N HCl (20 mL) was added till pH ~ 4-5 was obtained. Afterward, the mixture was extracted with EtOAc (3 x 40 mL). The combined organic fractions with the yellow color were discarded. The aqueous phase was basified by adding 10 N NaOH (5 mL) generating a pH value of ~10-

12 and forming a white precipitate. The basic aqueous phase was then extracted with Et_2O (2 x 40 mL). The combined organic fractions were washed with brine (50 mL), dried with Na_2SO_4, concentrated under reduced pressure and analyzed using NMR (^1H and ^{13}C in $CDCl_3$). 388 mg product (~17%) was obtained. The NMR (^1H and ^{13}C in $CDCl_3$) data were in agreement with those reported in the literature.[4] No further purification was needed.

b) Preparation of (*E*)-4-(4-chlorophenyl)but-3-en-2-amine (2c)

Synthesis of (3E)-4-(4-chlorophenyl)but-3-en-2-one oxime

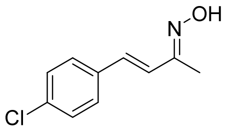

To a solution of (3*E*)-4-(4-chlorophenyl)but-3-en-2-one (2.0 g, 11 mmol) and $NH_2OH{\cdot}HCl$ (1.6 g, 23 mmol) in ethanol (20 mL, 80% v/v), NaOH (2.5 g, 64 mmol) was slowly added at room temperature. The reaction mixture was heated to 80 °C and refluxed overnight. The reaction was monitored by TLC (7:3 cyclohexane:EtOAc). After completion, the reaction was subsequently quenched by adding saturated aqueous NH_4Cl (150 mL) and 2 N HCl (15 mL, pH ~7). The reaction mixture was extracted with CH_2Cl_2 (3 x 100 mL). The combined organic fractions were washed with brine (100 mL), dried with Na_2SO_4 and concentrated under reduced pressure yielding a yellowish solid (1.82 g, 84%, $R_f = 0.6$, 7:3 cyclohexane:EtOAc). The NMR (^1H and ^{13}C in $CDCl_3$) data were in accordance with the literature.[5] The product of this step was used directly in the second step without further purification.

Synthesis (3E)-4-(4-chlorophenyl)but-3-en-2-amine (2c)

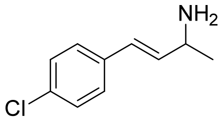

To an ice-cooled solution of (3E)-4-(4-chlorophenyl)but-3-en-2-one oxime (1.69 g, 9 mmol) in ethanol (20 mL) and acetic acid (20 mL), zinc dust (2.9 g, 46 mmol) was added slowly. The reaction mixture was stirred for 10 min on ice, subsequently warmed to room temperature and refluxed at 50 °C for 30 min. The formation of the product was monitored by TLC. After the completion, the zinc dust was filtered *via* celite (celite 500 fine) and washed with a generous amount of ethanol (200 mL). The filtrate was evaporated under reduced pressure until a thick and dark yellowish liquid was obtained. The residue was dissolved in distilled water (50 mL) generating a pH value of ~ 6-7, then 2 N HCl was added till pH ~ 3-4 was obtained. Afterward, the mixture was extracted with EtOAc (3 x 60 mL). The combined organic fractions with the yellow color were discarded. The aqueous phase was basified by adding 10 N NaOH generating a pH value of ~10-12 and forming a white precipitate. The basic aqueous phase was then extracted with Et_2O (2 x 60 mL). The combined organic fractions were washed with brine, dried with Na_2SO_4 and concentrated under reduced pressure yielding a yellowish oil (801 mg, 51%, $R_f = 0.35$, 7:3 cyclohexane:EtOAc). The NMR (^1H and ^{13}C in $CDCl_3$) data were in accordance with the literature.[6] No further purification was necessary.

c) Preparation of (3*E*)-4-(*p*-tolyl)but-3-en-2-amine (3c)

Synthesis of (3E)-4-(p-tolyl)but-3-en-2-one oxime

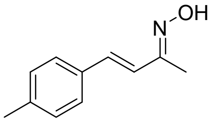

To a solution of (3*E*)-4-(*p*-tolyl)but-3-en-2-one (2 g, 12 mmol) and $NH_2OH·HCl$ (1.74 g, 25 mmol) in ethanol (18 mL, 80% v/v), NaOH (3 g, 37.5 mmol) was slowly added at room temperature over 20 min, then heated to 80 °C and refluxed overnight. The formation of the product was monitored by TLC (7:3 cyclohexane:EtOAc). After completion, the reaction mixture was quenched by adding saturated aqueous NH_4Cl (20 mL) and was subsequently extracted with CH_2Cl_2 (3 x 50 mL). The combined organic fractions were washed with brine (50 mL), dried with Na_2SO_4, and evaporated under reduced pressure. A yellow solid (2.10 g, ~96%) was obtained. The NMR (^1H and ^{13}C in $CDCl_3$) data were in agreement with the literature [5] and proved the formation and the purity of the desired product. The product of this step was used directly in the second step without further purification.

Synthesis of (3E)-4-(p-tolyl)but-3-en-2-amine (3c)

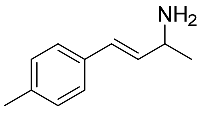

To an ice-cooled solution of (3*E*)-4-(p-tolyl)but-3-en-2-one oxime (1.5 g, 9 mmol) in ethanol (20 mL) and acetic acid (20 mL), zinc dust (6 g, 92 mmol) was added slowly. The reaction mixture was warmed to room temperature and subsequently heated to 50°C and refluxed for 1 h. The zinc dust was filtrated *via* celite (celite 500 fine) and washed with a generous amount of ethanol (200 mL). The solution was concentrated under reduced pressure until a reddish turbid liquid was obtained. The residue was dissolved in distilled water (50 mL), then 2 N HCl was added till pH ~ 3-4 leading to the formation of a white precipitate. The aqueous phase was extracted with EtOAc (3 x 40 mL). The combined organic fractions with reddish color were discarded. The aqueous phase was basified by adding 10 N NaOH (3 mL) forming a white precipitate (pH ~ 10-12) and was then extracted with Et_2O (3 x 50 mL). The combined ether fractions were washed with brine, dried with Na_2SO_4 and concentrated under reduced pressure yielding yellow oil (429 mg, ~31%). The NMR (^1H and ^{13}C in $CDCl_3$) data were in agreement with the literature[6] and proved the structure and the purity of the product.

d) Preparation of (3*E*)-4-(2,6,6-trimethylcyclohex-1-en-1-yl)but-3-en-2-amine (4c)

Synthesis of (3E)-4-(2,6,6-trimethylcyclohex-1-en-1-yl)but-3-en-2-one oxime

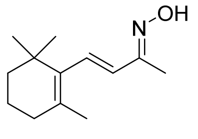

In a round-bottom, two-neck, 100 mL flask, a mixture of $NH_2OH·HCl$ (1.93 g, 24.7 mmol) and sodium acetate (3.30 g, 80.0 mmol) was dissolved with distilled water (5.0 mL), which was added with then an ethanol solution (12.5 mL) of β-ionone (5.26 g, 27.4 mmol). The reaction mixture was warmed to room temperature and subsequently heated to 50°C and refluxed for 4 h. The formation of the product was monitored by TLC. After completion, the reaction mixture was cooled down to room temperature and subsequently extracted with Et_2O (3 x 100 mL). The

combined organic fractions were washed with $NaHCO_3$ (10%, 50 mL) and water, then dried with Na_2SO_4 and evaporated under reduced pressure. A yellow solid (quant. yield) was obtained. The NMR (^1H and ^{13}C in $CDCl_3$) were recorded and showed the *E* and *Z* mixture of the desired oxime.[7] The product of this step was used directly in the second step without further purification.

Synthesis of (3E)-4-(2,6,6-trimethylcyclohex-1-en-1-yl)but-3-en-2-amine (4c)

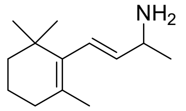

To an ice-cooled solution of (3*E*)-4-(2,6,6-trimethylcyclohex-1-en-1-yl)but-3-en-2-one oxime (1.25 g, 6 mmol) in ethanol (20 mL) and acetic acid (10 mL), zinc dust (2.0 g, 30 mmol) was added slowly. The reaction mixture was warmed to room temperature and subsequently heated to 50°C and refluxed for 45 min. The zinc dust was filtrated *via* celite (celite 500 fine) and washed with a generous amount of ethanol (200 mL). The solution was concentrated under reduced pressure until a turbid liquid was obtained. The residue was dissolved in distilled water (50 mL) and acidified using 2 N HCl (pH ~ 1-2). The aqueous phase was extracted with EtOAc (3 x 100 mL). The combined organic fractions with were discarded. Afterward, the aqueous phase was basified using 10 N NaOH forming a white precipitate (pH ~ 10-12) and then extracted with Et_2O (3 x 100 mL). The combined ether fractions were washed with brine, dried with Na_2SO_4 and concentrated under reduced pressure to yield a yellowish oil (292 mg, ~25%). The residue was subjected to the column to purify the desired amine (91 mg, ~8%). GC-MS of the final amine showed 13% of impurity with the mass of 221.2. The NMR (^1H and ^{13}C in $CDCl_3$) proved the formation of the desired amine.[8]

1.2.4. NMR of allylic oximes and amines

a) (3E)-4-phenylbut-3-en-2-one oxime

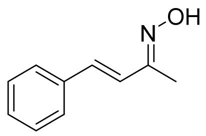

4.95 g (yield ~90%); ^1H NMR (300 MHz, $CDCl_3$): δ 9.34 (1H, br s, O**H**), 7.50 (2H, dt, *J* = 3.3, 2.0 Hz, Ar), 7.38 – 7.28 (3H, m, Ar), 6.97 – 6.85 (2H, m, **H**C=), 2.19 (3H, s, C**H₃**); ^{13}C NMR (75 MHz, $CDCl_3$): δ 156.8, 136.3, 133.4, 128.8, 128.5, 126.9, 125.7, 9.7.[3]

b) (3E)-4-phenylbut-3-en-2-amine

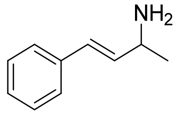

388 mg (yield ~17%); ^1H NMR (300 MHz, $CDCl_3$): δ 7.40 – 7.21 (5H, m, Ar), 6.50 (1H, d, *J* = 15.9 Hz, **H**C=), 6.23 (1H, dd, *J* = 15.9, 6.9 Hz, **H**C=), 3.79 – 3.70 (1H, m, H_2NC**H**), 1.82 (2H, br s, **H_2**N), 1.30 (3H, d, *J* = 6.5 Hz, C**H_3**); ^{13}C NMR (75 MHz, $CDCl_3$): δ 136.8, 134.6, 129.0, 128.6, 127.5, 126.3, 49.7, 23.5.[4]

c) (3E)-4-(4-chlorophenyl)but-3-en-2-one oxime

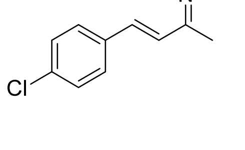

1.82 g (yield ~84%); ^1H NMR (300 MHz, $CDCl_3$) δ 9.14 (1H, br s, O**H**), 7.41 – 7.26 (4H, m, Ar), 6.91 – 6.79 (2H, m, Hz, =C**H**), 2.14 (3H, s, C**H_3**); ^{13}C NMR (75 MHz, $CDCl_3$): δ 156.6, 134.8, 134.1, 132.0, 129.0, 128.0, 126.4, 9.7.[5]

d) (3E)-4-(4-chlorophenyl)but-3-en-2-amine

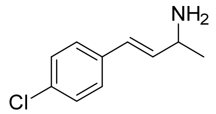

801 mg (yield ~51%); ^{1}H NMR (300 MHz, $CDCl_3$) δ 7.37 – 7.25 (m, 4H, Ar), 6.43 (1H, dd, *J* = 15.9, 1.0 Hz, **H**C=), 6.19 (1H, dd, *J* = 15.9, 6.6 Hz, **H**C=), 3.72 – 3.63 (1H, m, C**H**NH_2), 1.50 (2H, br s, N$\mathbf{H_2}$), 1.26 (3H, d, *J* = 6.5 Hz, C$\mathbf{H_3}$); ^{13}C NMR (75 MHz, $CDCl_3$) δ 136.8, 135.7, 132.8, 128.7, 127.5, 126.7, 49.2, 23.9.[6]

e) (3E)-4-(p-tolyl)but-3-en-2-one oxime

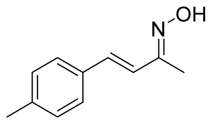

2.10 g (yield ~96%); ^{1}H NMR (300 MHz, $CDCl_3$): δ 8.98 (1H, br s, O**H**), 7.39 (2H, d, *J* = 8.1 Hz, Ar), 7.18 (2H, d, *J* = 7.9 Hz, Ar), 6.93– 6.79 (2H, m, =C**H**), 2.38 (3H, s, $\mathbf{H_3}$CC_6H_4), 2.16 (3H, s, C$\mathbf{H_3}$); ^{13}C NMR (75 MHz, $CDCl_3$): δ 156.9, 138.5, 133.5, 133.3, 129.5, 126.8, 124.8, 21.3, 9.7.[5]

f) (3E)-4-(p-tolyl)but-3-en-2-amine

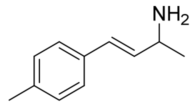

429 mg (yield ~31%); ^{1}H NMR (300 MHz, $CDCl_3$): δ 7.28 (2H, d, *J* = 8.1 Hz, Ar), 7.12 (2H, d, *J* = 8.1 Hz, Ar), 6.44 (1H, d, *J* = 15.9 Hz, =C**H**), 6.17 (1H, dd, *J* = 15.9, 6.7 Hz, =C**H**), 3.70 – 3.61 (1H, m, C**H**NH_2), 2.34 (3H, s, $\mathbf{H_3}$CC_6H_4), 1.63 (2H, s, $\mathbf{H_2}$N), 1.26 (3H, d, *J* = 6.5 Hz, C$\mathbf{H_3}$); ^{13}C NMR (75 MHz, $CDCl_3$): δ 137.0, 135.1, 134.4, 129.2, 127.7, 126.2, 49.4, 23.9, 21.2.[6]

g) (3E)-4-(2,6,6-trimethylcyclohex-1-en-1-yl)but-3-en-2-one oxime

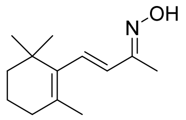

Mixture of *E* and *Z* from oxime, (quant. yield); ^{1}H NMR (300 MHz, $CDCl_3$): δ isomer 1: 9.52 (1H, br S, OH), 6.91 (1H, d, *J* = 16.7 Hz, =C**H**), 6.52 (1H, dd, *J* = 16.5, 0.8 Hz, =C**H**), 2.07 (3H, s, C$\mathbf{H_3}$), 2.03 (2H, m, C$\mathbf{H_2}$), 1.76 (3H, s, C$\mathbf{H_3}$), 1.65 – 1.61 (2H, m, C$\mathbf{H_2}$), 1.50 – 1.46 (2H, m, C$\mathbf{H_2}$), 1.06 (6H, s, 2 x C$\mathbf{H_3}$); δ isomer 2: 9.52 (1H, br S, OH), 6.61 (1H, dd, *J* = 16.7, 0.8 Hz, =C**H**), 6.15 (1H, d, *J* = 16.5 Hz, =C**H**), 2.08 (3H, s, C$\mathbf{H_3}$), 1.72 (3H, s, C$\mathbf{H_3}$), 1.65 – 1.61 (2H, m, C$\mathbf{H_2}$), 1.50 – 1.46 (2H, m, C$\mathbf{H_2}$),1.04 (6H, s, 2 x C$\mathbf{H_3}$);^{13}C NMR (75 MHz, $CDCl_3$): δ isomer 1: 153.5, 136.9, 135.9, 132.2, 121.5, 39.6, 34.0, 33.2, 28.9, 21.7, 19.1, 16.7. δ isomer 2: 156.7, 136.8, 132.5, 130.8, 129.8, 39.6, 34.1, 33.0, 28.8, 21.6, 19.1, 9.6.[7]

h) (3E)-4-(2,6,6-trimethylcyclohex-1-en-1-yl)but-3-en-2-amine

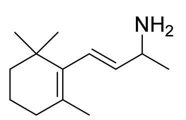

91 mg (yield ~8%), ^{1}H NMR (300 MHz, $CDCl_3$): δ 5.92 (1H, d, *J* = 15.9 Hz, =C**H**), 5.39 (1H dd, *J* = 13.9, 6.9 Hz, =C**H**), 3.56 – 3.51 (1H, m, C**H**NH_2), 1.96 (2H, t, *J* = 6.1 Hz, C$\mathbf{H_2}$), 1.65 (3H, s, C$\mathbf{H_3}$), 1.61 – 1.56 (2H, m, C$\mathbf{H_2}$), 1.48 – 1.41 (2H, m, C$\mathbf{H_2}$), 1.19 (3H d, *J* = 6.5 Hz, H_2NCHC$\mathbf{H_3}$), 0.98 (6H, s, 2 x C$\mathbf{H_3}$); ^{13}C NMR (75 MHz, $CDCl_3$): δ 140.1, 137.0, 128.1, 125.5, 49.8, 39.4, 33.9, 32.6, 28.7, 24.1, 21.3, 19.3.[8]

1.2.5. NMR of isolated allylic amines from upscaling

a) (R,E)-4-phenylbut-3-en-2-amine

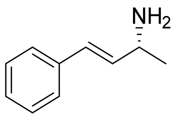

4.9 mg (yield ~30 %), $[\alpha]^{20}_{D}$ +22.5 (C=0.4, $CHCl_3$, *ee* 97%), lit $[\alpha]^{20}_{D}$ +25.8 (C=1.16, $CHCl_3$, *ee* >98% [9]); ^{1}H NMR (300 MHz, $CDCl_3$): δ 7.40 – 7.22 (5H, m, C_6**H_5**), 6.51 (1H, d, *J* = 16.0 Hz, =C**H**), 6.23 (1H, dd, *J* = 15.9, 6.9 Hz, =C**H**), 3.79 – 3.70 (1H, m, H_2NC**H**), 1.69 (2H, br s, **H_2**N), 1.29 (3H, d, *J* = 6.5 Hz, C**H_3**).

b) (R,E)-4-(4-chlorophenyl)but-3-en-2-amine

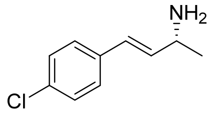

4.1 mg (yield ~25%), $[\alpha]^{20}_{D}$ +18.0 (C=0.1, $CHCl_3$, *ee* 97%); ^{1}H NMR (300 MHz, $CDCl_3$): δ 7.33 – 7.28 (m, 4H, Ar), 6.44 (1H, dd, *J* = 15.9, 0.9 Hz, =C**H**), 6.20 (1H, dd, *J* = 15.9, 6.6 Hz, =C**H**), 3.71 – 3.66 (1H, m, C**H**NH_2), 1.56 (2H, br s, N**H_2**), 1.27 (3H, d, *J* = 6.5 Hz, C**H_3**).

c) (R,E)-4-(p-tolyl)but-3-en-2-amine

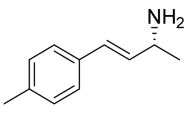

4.6 mg (yield ~41%), $[\alpha]^{20}_{D}$ +36.1 (C=0.5, $CHCl_3$, *ee* 97%); ^{1}H NMR (300 MHz, $CDCl_3$): δ 7.28 (2H, d, *J* = 8.0 Hz, Ar), 7.13 (2H, d, *J* = 8.0 Hz, Ar), 6.45 (1H, d, *J* = 15.9 Hz, =C**H**), 6.17 (1H, dd, *J* = 15.9, 6.7 Hz, =C**H**), 3.77 – 3.63 (1H, m, H_2NC**H**), 2.35 (3H, s, **H_3**CC_6H_4), 1.55 (2H, s, **H_2**N), 1.27 (3H, d, *J* = 6.5 Hz, C**H_3**).

1.3. Biocatalytic reactions

1.3.1. Preparation of the biocatalysts

For the oxidation step, PQQ-DH was expressed, lyophilized, and used as whole cells in the biotransformations.

For the amination step, the transaminase from *Arthrobacter* sp.– ArRmut11[10] (mutations: S8P; Y60F; L61Y; H62T; V65A; V69T; D81G; M94I; I96L; F122M; S124T; S126T; G136F; Y150S; V152C; A169L; V199I; A209L; G215C; G217N; S223P; L269P; L273Y; T282S; A284G; P297S; S321P) was expressed, lyophilized, and used in the biotransformations. Besides, other transaminases including TA from *Alcaligenes denitrifcans* (AD-ωTA-pET21a, pEG 19)[11], TA from *Arthrobacter sp.* (Ar-*R*-ωTA-pET21a, pEG 23)[12], TA from *Paracoccus denitrificans* (pCR6-StrepII, pEG 24)[13], TA from *Pseudomonas putida* (pCR7-StrepII, pEG 25)[14], TA from *Arthrobacter citreus* (Ar*S*-ωTA-His, pEG 29)[15], TA from *Alcaligenes denitrificans* (AD-ωTA-His, pEG 30)[16], TA from *Bacillus megaterium* (BM-ωTA-His, pEG 31)[17], TA from *Arcobacter aurescens* (TA_R-Arth1-A1RAS9, pEG 70), TA from *Aspergillus tereus* (TA(*R*)-AspTerr (1077745), pEG 97)[10b,18], TA from *Hyphomonas neptunium* (TA(*R*)-HyphNep (1077747), pEG 98)[10b,18], TA from *Pseudomonas fluorescens* (ωTA-pf, pEG 148)[13b], TA from *Giberella zeae* (GZ-ωTA in pET21a, pEG 202)[18,19], TA from *Neosartorya fischeri* (NF-ωTA in pET21a, pEG 203)[18,19], TA from *Ochrobactrum anthropi* (OA-ωTA in pET21a, pEG

204)[19,20], TA from *Silicibacter pomeroyi* (SP-ωTA in pET21a, pEG 205)[19,21] and TA from *Vibrio fluvialis* (VFmut-ωTA, pEG 209)[22] were tested in the amination step using (*R*)/(*S*)-1-phenylethylamine and isopropylamine as amine donors.

a) PQQ-DH (pEG 404)

<u>DNA sequence</u>

catATGCATGCCGATGGTGCAGCAGCAGAAACCGCAGCACCGGGTCAGAGCGCAATTGAA
AACTTTCAGCCGGTTACAGCCGAAGATCTGGCAGGCGGTAATGCAGCAAATTGGCCGATT
CTGCGTGGTAATTATCAAGGTTGGGGTTATACCCAGCTGGATCAGATTAACAAAGATAAT
GTTGGTCAGCTGCAACTGGCATGGGCACGTACCATGGAACCGGGTAGCAATGAAGGTAG
CGCCATTGCATATAATGGTGTTGTTTTTCTGGGCAATGCCAATGATGTTGTTCAGGCAATT
GATGGTAAAACCGGTAATCTGATTTGGGAGTATCGTCGTAAACTGCCTCCGGCAAGCAAA
TTTATCAATAGCCTGGGTGCAGCAAAACGTAGCATTGCACTGTTTGGTGATAAAGTGTAT
TTTGTGAGCTGGGATAATTTTGTTGTTGCCCTGGATGCAAAAACCGGCAAACTGGCCTGG
GAAACCAATCGTGGTCAGGGTGTTGAAGAAGGTGTTAGCAATAGCAGCGGTCCGATTGTT
GTTGATGGTGTGGTTATTGCAGGTAGCACCTGTCAGTATAGCGGTTTTGGTTGTTATGTTA
CCGGCACCGATGCAGAAAGCGGTGAAGAACTGTGGCGTAATACCTTTATTCCGCGTCCGG
GTGAAGAGGGTGATGATACCTGGGGTGGTGCACCGTATGAAAATCGTTGGATGACCGGT
GCATGGGGTCAGATTACGTATGATCCGGAACTGGATCTGGTTTATTATGGTAGTACCGGT
GCAGGTCCGGCAAGTGAAGTTCAGCGTGGCACCGAAGGTGGCACCCTGGCAGGTACAAA
TACCCGTTTTGCAGTTAAACCGAAAACCGGTGAAGTTGTTTGGAAACATCAGACCCTGCC
TCGTGATAATTGGGATAGCGAATGTACCTTTGAAATGATGGTTGTTAGCACCACCGTTAA
TCCGGATGCGGGTGCAGATGGTATGATGAGCGTTGGTGCAAATGTTCCGCGTGGTGAAAC
CCGTAAAGTTCTGACCGGTGTTCCGTGTAAAACAGGTGTTGCATGGCAGTTTGATGCCGA
AACAGGTGATTACTTTTGGAGCAAAGCAACCGTTGAACAGAATAGCATTGCCAGCATTGA
TGATAAAGGTCTGGTTACCGTTAACGAGGATATGATTCTGAAAGAACCGGGAAAAGATT
ATAACTATTGCCCGACCTTTTTAGGTGGTCGTGATTGGCCGAGCGCAGGTTATCTGCCGA
AAAGCAATCTGTATGTTATTCCGCTGAGCAATGCATGCTATGACCTGAAAGCCCGTACCA
CCGAAGCAACACCGGCAGATGTGTATAATACCGATAGCACCGTTAAACTGGCACCGGGT
AAAACCAATATGGGTCGTGTTGATGCGATTGATGTTGCAACCGGTGCCACCAAATGGTCA
TTTGAAACCGAAGCAGCACTGTATGATCCTGTTATGACCACCGCAGGCGATCTGGTGTTT
GTTGGTAGCACCGATCGTATGTTTCGTGCACTGGATGCGGAAACGGGTAAAGAAGTTTGG
AGCACCCGTCTGCCTGGTGCAATTAGCGGTTATACCACCAGTTATAGTATTGATGGTCGTC
AGTATGTTGCAGTTGTTGCCGGTGGTAGCTTAGGTACAGGTTTTTTCAAAGCAGCAGTTCC
TGGTGTGGATGCAGTTCAAGGTGGTAATGGTATTTATGTTTTTGCACTGCCGGAAGCCAA
ATAA

Amino acid sequence

MHADGAAAETAAPGQSAIENFQPVTAEDLAGGNAANWPILRGNYQGWGYTQLDQINKDNV
GQLQLAWARTMEPGSNEGSAIAYNGVVFLGNANDVVQAIDGKTGNLIWEYRRKLPPASKFIN
SLGAAKRSIALFGDKVYFVSWDNFVVALDAKTGKLAWETNRGQGVEEGVSNSSGPIVVDGV
VIAGSTCQYSGFGCYVTGTDAESGEELWRNTFIPRPGEEGDDTWGGAPYENRWMTGAWGQI
TYDPELDLVYYGSTGAGPASEVQRGTEGGTLAGTNTRFAVKPKTGEVVWKHQTLPRDNWDS
ECTFEMMVVSTTVNPDAGADGMMSVGANVPRGETRKVLTGVPCKTGVAWQFDAETGDYF
WSKATVEQNSIASIDDKGLVTVNEDMILKEPGKDYNYCPTFLGGRDWPSAGYLPKSNLYVIP
LSNACYDLKARTTEATPADVYNTDSTVKLAPGKTNMGRVDAIDVATGATKWSFETEAALYD
PVMTTAGDLVFVGSTDRMFRALDAETGKEVWSTRLPGAISGYTTSYSIDGRQYVAVVAGGSL
GTGFFKAAVPGVDAVQGGNGIYVFALPEAK

Expression: For PQQ-DH expression, an overnight culture of *E. coli* BL21(DE3) cells bearing the encoding plasmid (PQQ-DH_pET28a(+) (UniProt A0A087LLP8)) was prepared. For this purpose, 15 mL of Lysogeny Broth containing 0.1% v/v of kanamycine stock was inoculated with 10 µL of glycerol stock and incubated overnight at 30 °C. To an LB medium containing 0.1% v/v of kanamaycine, 1% v/v of the overnight culture was added and incubated at 37 °C until it reached an OD_{600} of 0.6-0.8. Cells were induced with isopropyl-*β*-D-thiogalactopyranoside (IPTG, 0.5 mM) and grown overnight at 20 °C. Cells were harvested by centrifugation at 3730 ×g for 20 min (Hettich® Rotina 420R centrifuge, 4 °C), resuspended in KPi buffer (50 mM, pH 7.5), frozen in liquid nitrogen and lyophilized. Lyophilized cells were used in the biotransformations.

In case of activity assay, the purified enzyme was used. For that purpose, cells after harvesting and washing were resuspended in Tris-HCl (35 mL, 100 mM, pH 8.0) supplemented with NaCl (150 mM) and PQQ (25 µM). The cell extract was obtained by sonification with a Branson Digital Sonifier 250 (30 % amplitude, 2 min, 2 sec pulse, 4 sec pause). The lysate was cleared by centrifugation (20000×g for 20 min) and after filtration was objected to the purification using HisTrap FF column.

Purification: His-Tagged PQQ-DH was purified by immobilized Ni-affinity chromatography (5 mL HisTrap FF column, GE Healthcare) using a syringe and applying a 5 to 500 mM gradient of imidazole (binding buffer: Tris-HCl, 100 mM, pH 8.0 containing 150 mM NaCl and 5 mM imidazole; elution buffer: Tris-HCl, 100 mM, pH 8.0 containing 150 mM NaCl and 500 mM imidazole). Collected fractions were analyzed by SDS-PAGE. Fractions containing PQQ-DH were pooled, concentrated by ultrafiltration (20 mL, 30 kDa cut-off, Vivaspin) and desalted (SephadexTM G-25 M, GE Healthcare) with Tris-HCl, 100 mM, pH 8.0 containing 60 µM PQQ. After desalting the fractions were stored at 4 °C overnight to be used in the biotransformations next day. The remaining fractions were frozen and stored at -20 °C.

b) ArRmut11-ωTA (pEG 90)

Expression: For ArRmut11-ωTA expression, an overnight culture of *E. coli* BL21(DE3) cells bearing the encoding plasmid (pEG 90, pTA_MutR11_ pET21a) was prepared. For this purpose, 15 mL of Lysogeny Broth containing 15 µL of ampicillin stock was inoculated with 10 µL of glycerol stock and incubated overnight at 30 °C. LB-Amp medium (330 mL, 100 mg/L ampicillin) was inoculated with cell suspension from an overnight culture (5 mL) and cells were grown at 30°C at 120 rpm until it reached an OD600 of 0.5. Cells were induced with isopropyl-β-D-thiogalactopyranoside (IPTG, 0.5 mM) and grown overnight at 20 °C. Cells were harvested by centrifugation at 3730 ×g for 20 min (Hettich® Rotina 420R centrifuge, 4 °C) and washed with KPi buffer (50 mM, pH 7.5 containing 0.5 mM PLP) frozen with liquid nitrogen and lyophilized.

1.3.2. General protocols for the biotransformations

a) Oxidation step using PQQ-DH

The oxidation step of the cascade was run in 4 mL glass vials under the conditions described below:

The lyophilized PQQ-DH cells (20 mg/mL final concentration in 1 mL reaction volume), PQQ (100 µM final concentration), potassium ferricyanide (PFC- 20 mM final concentration), the substrate (10 mM final concentration, 2% v/v DMSO) were added to the buffer (KPi, 200 mM, pH 7.0). The biotransformation vials were incubated 48 h at 21 ºC (170 rpm, horizontal shaking). After 48 h, the extraction was done using EtOAc (2 x 500 µL). Combined organic phases were dried with Na_2SO_4. Samples were prepared from the dried organic phase without further treatment and measured on GC-MS.
When substrate concentration was increased to 50 mM, 10% v/v of DMSO was used. In addition, potassium ferricyanide (PFC- 100 mM final concentration), PQQ (225 µM final concentration) and the lyophilized PQQ-DH cells (50 mg/mL final concentration) were used and added to the buffer (KPi, 200 mM, pH 7.0).

b) Activity assay using purified PQQ-DH

For the activity assay of the purified PQQ-DH with different substrates, first the concentration of the purified enzyme which was desalted with Tris-HCl buffer (100 mM, pH 7.5) was measured using Bradford protein assay. Bradford reagent was diluted 1:4 with water. The protein solution (20 µL) was put in the reagent solution (980 µL) and incubated at room temperature for 15 min before the measurement in the photometer at a wavelength of 595 nm.

Substrate stock solutions were prepared for the activity assay as well as for the biotransformations with varying reaction conditions. All stock solutions were prepared in buffer (Tris-HCl, 100 mM, pH 7.5).

The purified enzyme (90 µL, 1 mg/mL final concentration in 1 mL reaction volume), PFC stock solution (20 µL from 1 M stock, 20 mM final concentration), substrate buffer stock solution (700 µL from 14.3 mM stock, 10 mM final concentration), PQQ stock solution (13.5 µL from 7.5 mM stock, 100 µM final concentration) and additional buffer (176.5 µL, Tris-HCl, 100 mM, pH 7.5) were mixed in 4 mL glass vials. In addition, one blank per substrate was prepared with the same composition and buffer instead of enzyme solution. In case of 1-phenylethan-1-ol, only one blank with the racemic compound was prepared. All samples were then shaken overnight with 170 rpm at room temperature. The extraction was done with EtOAc (2 × 500 µL). Combined organic phases were dried with Na_2SO_4. Samples were prepared from the dried organic phase without further treatment and measured on GC-MS.

c) Amination step using ω-TAs

In the amination step, various transaminases were implemented as the biocatalysts to transform the allylic ketones (**1b-4b**) into their corresponding amines. For substrates, stocks with 0.5 M concentration in DMSO were prepared, then the desired volume (100 µL for 50 mM and 20 µL for 10 mM final substrate concentration in 1 mL reaction volume resulting 10% v/v and 2% v/v DMSO in final reaction volume, respectively) from these stocks was added to the reaction mixture (see details in Table S2 and S3).

Isopropylamine (**5c**), 1,2-diaminocyclohexane, (*R*)-1-phenylethylamine ((*R*)-**6c**) and (*S*)-1-phenylethylamine ((*S*)-**6c**) with different concentration (0.3-2.5 M final concentration in 1 mL reaction volume) were used as amine donors. For the amine donors, 5 M stocks were prepared, and the pH was adjusted to pH 8 using aqueous HCl (35%), then the desired volume (100 µL for 0.5 M, 200 µL for 1.0 M, 250 µL for 1.25 M and 500 µL for 2.5 M final amine donor concentration in 1 mL reaction volume) from these stocks were added to the reaction mixtures. The application of isopropylamine as an amine donor has a wide range of advantages: isopropylamine is achiral, volatile, and inexpensive. Moreover, the oxidized co-product – acetone – is volatile. Consequently, the equilibrium can be easily shifted towards product formation *via* a gas sweep or under reduced pressure. However, in contrast to these summarized advantages, isopropylamine is accepted only by a limited number of transaminases. In this context, Savile and collaborators described the development of a variant of the transaminase from *Arthrobacter* sp.– the ArRmut11. This mutant was designed to accept bulky-bulky ketones, high concentrations of isopropylamine and to exhibit solvent and temperature tolerance.[10]

In addition to ArRmut11, other ω-TAs (40 mg/mL final concentration of lyophilized cells in 1 mL reaction volume) were tested in the amination step as well. ω-TAs lyophilized cells were rehydrated in buffer containing PLP (2.5 mM stock concentration for PLP and 100 mg/mL stock concentration for cells with pH adjusted to 8.0), then 400 µL from this stock was taken and added to the reaction mixture resulting 40 mg/mL final concentration of lyophilized cells and 1 mM PLP in 1 mL reaction volume. Additional buffer was used to reach 1 mL reaction volume (400 µL for 0.5 M amine donor concentration,

300 µL for 1.0 M amine donor concentration and 250 µL for 1.25 M amine donor concentration). In the case of other ω-TAs (except ArRmut11), 10 mM substrate concentration was used. All reactions were done in triplicates. Blank samples were prepared for each substrate, which had the same composition as the other reaction mixtures, but instead of the cell solution, only buffer was used. The biotransformation samples using ArRmut11 were incubated overnight at 40 ºC (450 rpm, horizontal shaking), while other transaminases were incubated overnight at 30 ºC (450 rpm, horizontal shaking). For working up, the reactions were basified using 100 µL of 10 N NaOH and subsequently extracted with EtOAc (3 x 500 µL), dried with Na_2SO_4 and analyzed using GC-FID without further treatment.

Table S2 Details on the reaction set-up for the amination step with 50 mM substrate concentration, 10% v/v DMSO and various amine donor concentration

Sub. (0.5 M stock in DMSO)	Amine donor (5 M stock)	Final conc. of amine donor	KPi (100 mg/cell and 2.5 mM PLP)	Additional buffer
100 µL	100 µL	0.5 M	400 µL	400 µL
100 µL	200 µL	1.0 M	400 µL	300 µL
100 µL	250 µL	1.25 M	400 µL	250 µL
100 µL	500 µL	2.5 M	400 µL	-

Table S3. Details on the reaction set-up for the amination step with 10 mM substrate concentration, 2% v/v DMSO and 300 mM-2.5 M amine donor

Sub. (0.5 M stock in DMSO)	Amine donor (5 M stock)	Final conc. of amine donor	KPi (100 mg/cell and 2.5 mM PLP)	Additional buffer
20 µL	60 µL	300 mM	400 µL	520 µL
20 µL	100 µL	500 mM	400 µL	480 µL
20 µL	200 µL	1 M	400 µL	380 µL
20 µL	400 µL	2 M	400 µL	180 µL
20 µL	500 µL	2.5 M	400 µL	80 µL

d) Amination step using ArRmut11 at different pH

To check the pH effect on the amination step, ArRmut11 was implemented as the biocatalyst to transform the allylic ketones (**1b-4b**) into their corresponding amine at different pH levels. For the substrate, a stock with 0.5 M concentration in DMSO was prepared, then the desired volume (100 µL for 50 mM final substrate concentration in 1 mL reaction volume resulting 10% v/v) from this stock was withdrawn and added to the reaction mixture. (*R*)-1-Phenylethylamine ((*R*)-**6c**) with 1.25 M final concentration was used as an amine donor. For the amine donor, three different stocks (5 M) were prepared and the pH of each was adjusted to pH 8.0, 9.0 or 10.0 using aqueous HCl (35%), then the desired volume (250 µL for 1.25 M final amine donor concentration in 1 mL reaction volume) from these stocks were withdrawn and added to the reaction mixtures, which were KPi buffers (200 mM) with the pH values adjusted to pH 8.0, 9.0 or 10.0. ω-TA lyophilized cells were rehydrated separately in buffers containing PLP (2.5 mM stock concentration for PLP and 100 mg/mL stock concentration for cells with pH adjusted to 8.0, 9.0 or 10.0), then 400 µL from this stock was withdrawn and added to the reaction mixture resulting 40 mg/mL final concentration of lyophilized cells and 1 mM PLP in 1 mL

reaction volume. Additional buffers with proper pH levels (pH 8.0, 9.0 or 10.0) were used to reach 1 mL reaction volume (250 µL).

All reactions were done in triplicates. Blank samples were prepared for each substrate, which had the same composition as the other reaction mixtures, but instead of the cell solution, only buffer was used. The biotransformation samples were incubated overnight at 40 ºC (450 rpm, horizontal shaking). For working up, the reactions were basified using 100 µL of 10 N NaOH and subsequently extracted with EtOAc (3 x 500 µL), dried with Na_2SO_4 and analyzed using GC-FID without further treatment.

e) Cascade set-ups

The cascade was performed in a one-pot two-step fashion. For this purpose, first, the oxidation step was performed using PQQ-DH in KPi (200 mM, pH 7.0) for 48 h (170 rpm, 21 ºC, horizontal shaking) in 4 mL glass vials with 1 mL total reaction volume. All substrates (racemic alcohols) with 10 mM concentration were subjected to the oxidation step. After 48 h reaction time, the transaminase (lyophilized cells) and the amine donor in KPi buffer (200 mM, pH 8.0) were added to the reaction mixture resulting in 1.5 mL total reaction volume. For PLP, a stock with 12 mM concentration in KPi (200 mM, pH 8.0) was prepared and 125 µL from this stock was added to the reaction vessels resulting in 1 mM final concentration of PLP in 1.5 mL total reaction volume. Isopropylamine (**5c**) and (*R*)-phenylethylamine ((*R*)-**6c**) were used as amine donors.

The samples were shaken another 24 h at 40 ºC (350 rpm, horizontal shaking). After 24 h, the samples were basified using 10 N NaOH (300 µL), then each sample was divided equally between two 2 mL eppis (750 µL in each eppi). Afterward, each eppi was extracted with EtOAc (3 x 500 µL) and dried with Na_2SO_4. Equal organic phases were combined in a 4 mL glass vial and dried under compressed air flow, afterward, the samples were re-dissolved in 800 µL EtOAc plus 200 µL MeOH and analyzed using GC-MS without further treatment. When running the cascade, blank reactions, as well as control reactions including the samples for measuring the conversion of oxidation step and samples for measuring the conversion of the amination step were performed in parallel.

f) Alcohol amination cascade on 0.2 mmol scale

The cascade was performed in 20 mL in a one-pot two-step fashion. For this purpose, first the oxidation step was performed using lyophilized PQQ-DH cells (300 mg, 20 mg/mL final concentration in 15 mL total reaction volume), which were rehydrated in KPi buffer (14.2 mL, 200 mM, pH 7.0) containing PQQ (200 µL from 7.5 mM stock, 100 µM final concentration), PFC (300 µL from 1 M stock, 20 mM final concentration) and substrate (300 µL from 0.5 M stock in DMSO, 10 mM final concentration, 2% v/v DMSO) for 48 h (170 rpm, 21 ºC, horizontal shaking) in a 50 mL falcon tube with 15 mL total reaction volume. After 48 h reaction time, the lyophilized ArRmut11 cells (800 mg, 40 mg/mL final concentration in 20 mL total reaction volume), PLP (5.3 mg, 1 mM final concentration), the amine donor (5 mL from 5 M stock for isopropylamine (**5c**), 1.25 M final concentration; or 1 mL from 5 M stock for

(*R*)-1-phenylethylamine ((*R*)-**6c**), 250 mM final concentration) in KPi buffer (200 mM, pH 8.0) were added to the reaction mixture resulting 20 mL total reaction volume.

The pH was adjusted to 8.0 and the samples were shaken another 24 h at 40 ºC (350 rpm, horizontal shaking). Afterward, the aqueous phase was extracted with CH_2Cl_2 (5 x 50 mL). Then the aqueous phase was checked on TLC to make sure that no ketone is left in the aqueous phase. The combined organic fractions were discarded. Afterward, the aqueous phase was basified (pH ~ 11-12) using 10 N NaOH and then extracted with CH_2Cl_2 (3 x 50 mL). The combined organic fractions were washed with brine, dried with Na_2SO_4 and concentrated under reduced pressure. GC-MS samples were prepared and analyzed. Purification of the final amines was done using preparative TLC using toluene/MeOH/TEA (20:1:0.2%). After recording NMRs and measuring the optical rotations, samples were derivatized and HPLC measurements using these samples were done to prove the optical purity and the absolute configuration of final (*R*)-amine.

g) Biocatalytic approach for the synthesis of (R)- and (S)-4c

<u>Using ArRmut11 ((R)-selective transaminase)</u>

To identify the enantiomers of **4c**, the amination of β-ionone (**4b**) was performed in KPi buffer (200 mM, pH 8.0) containing the ArRmut11 (40 mg/mL), isopropylamine (**5c**, 3 M), PLP (1 mM), the substrate **4b** (50 mM final concentration) with 10% v/v DMSO as cosolvent in 20 mL total reaction volume. The reaction mixture was shaken horizontally for 72 h (450 rpm, 40 ºC) (Scheme S1). After 72 h reaction time, 7% conversion was observed.

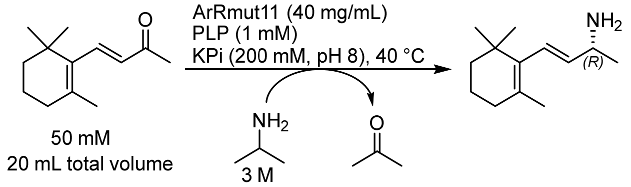

Scheme S1. Amination of β-ionone implementing ArRmut11 transaminases and isopropylamine (**5c**, 3 M) as the amine donor

After the work-up, using the method mentioned above, derivatization using DMAP and acetic anhydride was done, and this sample was measured on HPLC equipped with OJ column for the identification of (*R*)-**4c**.

<u>Using ATA-117 ((S)-selective transaminase)</u>

To identify the enantiomers of the amine **4c**, the amination of β-ionone (**4b**) was performed in KPi buffer (200 mM, pH 8.0) containing ATA-117 (from transaminases kit) (5 mg/mL), D-alanine (250 mM), PLP (1 mM), the substrate **4b** (20 mM final concentration) with 10% v/v DMSO as cosolvent in 17 mL total reaction volume. The reaction mixture was shaken horizontally for 72 h (450 rpm, 30 ºC) (Scheme S2). After 72 h reaction time, 4% conversion was observed.

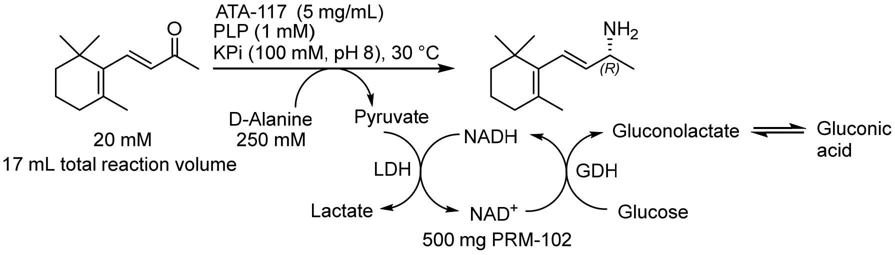

Scheme S2. Amination of *β*-ionone implementing ATA-117 transaminases and D-alanine (250 mM) as amine donor

After the work-up, using the method mentioned above, derivatization using DMAP and acetic anhydride was done, and this sample was measured on HPLC equipped with OJ column for the identification of (*S*)-**4c**.

1.4. Absolute configuration determination

Absolute configurations were assigned by the comparison of the elution order of the enantiomers on chiral HPLC with the published data (see Table S23-26). For measuring the enantiomeric excess of the final amines, samples were derivatized. For that purpose, 1 mL of the organic phase (ethyl acetate) was transferred into 2 mL eppis, then 4-(*N*,*N*-dimethylamino)pyridine (DMAP) was added to the solutions. Afterward, 25 µL of acetic anhydride was added to the reaction mixture and shaken overnight (650 rpm, 30 ºC). Afterward, 400 µL H_2O was added to the mixture to remove the unreacted acetic anhydride and the mixture was shaken for another hour. Afterward, the water was removed, and the mixture was dried with Na_2SO_4. For measuring the samples on HPLC, the solvent (EtOAc) was removed under compressed airflow, then the residue was re-dissolved in the mixture of *n*-heptane/isopropanol (8:2).

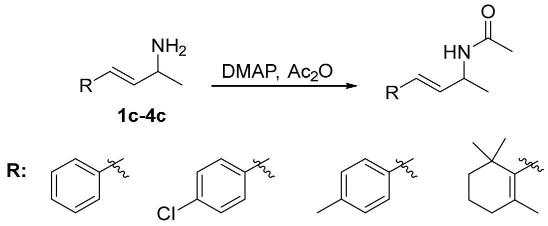

Scheme S3. Derivatization of amines

2. Results

2.1. Oxidation using PQQ-DH

a) PQQ-DH purification

The purification of PQQ-DH was done using the procedure mentioned before and SDS-PAGE by loading 15 µg of protein for each fraction was done. The SDS-PAGE is shown in Figure S1.

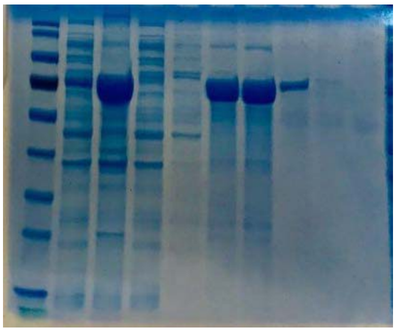

a b c d e f g h i j

Figure S1. **Gel A**: a) page ruler, b) PQQ-DH supernatant, c) PQQ-DH pellets, d) PQQ-DH flow through, e) PQQ-DH washing fraction, f) PQQ-DH elution fraction 1, g) PQQ-DH elution fraction 2, h) PQQ-DH elution fraction 3, i) PQQ-DH elution fraction 4, j) Empty

Elution fractions 1 to 3 were added to each other, concentrated, and desalted using procedure mentioned in the method section. The concentration of the enzyme was measured by Bradford assay before using in the biotransformations.

b) Testing the purified PQQ-DH in the oxidation of various substrates

In order to define the specific activity of the enzyme, time study using various enzyme concentration was performed. Results show that when 0.5 mg/mL of the purified enzyme was used, after 3.5 h conversions higher than 90% was observed (Table S4).

Table S4. Time study for the oxidation of various substrates using purified PQQ-DH [a]

		Conv. [%]		
**Sub.**	**Reaction time [h]**	**0.1 [mg/mL]**	**0.5 [mg/mL]**	**1.0 [mg/mL]**
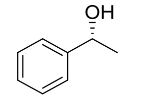	15 min	9	n.a.[b]	>99
	0.5	40	n.a.	>99
	1.5	79	84	>99
	3.5	84	90	>99
(*R*)-1-phenylethan-1-ol	20	>99	>99	>99
	0.5	49	n.a.[b]	>99
***rac*-1a**	1.5	78	89	>99
	3.5	84	90	>99
	20	91	>99	>99
	0.5	26	n.a.[b]	>99
***rac*-2a**	1.5	50	83	>99
	3.5	66	>99	>99
	20	83	>99	>99
	0.5	43	n.a.[b]	>99
***rac*-3a**	1.5	72	>99	>99
	3.5	97	>99	>99
	20	97	>99	>99
	0.5	53	n.a.[b]	>99
***rac*-4a**	1.5	60	89	>99
	3.5	67	>99	>99
	20	90	>99	>99

[a] Condition: Tris-HCl (100 mM, pH 7.5) containing purified PQQ-DH (0.1 -1.0 mg/mL final concentration in 1 mL reaction volume), PQQ (100 µM final concentration), PFC (20 mM final concentration), the substrate (10 mM final concentration) without cosolvent. The reaction mixtures and blanks were shaken overnight (170 rpm, 21ºC) and extracted with ethyl acetate (2 x 500 µL), dried with Na_2SO_4 and measured by GC-FID (low boiler method). Conversions based on area percentages of the formed ketone peaks. Reactions have been done in duplicate and the average of data was reported.
GC Method: Injector temperature: 250 °C; Injection volume: 5 µL; Flow rate: 0.7 mL/min; Temperature program (low boilers method): 40 °C, hold time 2.0 min, 10 °C/min to 180 °C, hold time 1.0 min. using HP-5 column (30 m x 0.32 mm x 0.25 µm, J&W Scientific, Agilent Technologies) using He as carrier gas.
[b] Data not available

c) Oxidation step employing E. coli/PQQ-DH cells

Table S5. Time study for the oxidation of substrates *rac*-**1a**-**4a** using lyophilized *E. coli*/PQQ-DH [a]

Substrates	Reaction time [h]	Conversion [%]	ee_s [%]	E[b]
	1	52	83	19
	2	64	98	
rac-**1a**	3	69	>98	
	7	74	98	
	24	90	98	
	1	36	51	
	2	53	87	22
rac-**2a**	3	64	>98	
	7	83	>98	
	24	98	>98	
	1	48	71	16
	2	73	>98	
rac-**3a**	3	78	>98	
	7	87	>98	
	24	95	>98	
	1	56	>98	41
rac-**4a**	2	64	>98	
	3	75	>98	
	7	94	>98	
	24	98	>98	

[a] Condition: Tris-HCl (100 mM, pH 7.5) containing the lyophilized PQQ-DH cells (20 mg/mL), PQQ (100 µM final concentration in 1 mL reaction volume), potassium ferricyanide (PFC, 20 mM final concentration in 1 mL reaction volume), the substrate (10 mM final concentration in 1 mL reaction volume) without cosolvent. The reaction mixtures and blanks were shaken overnight (170 rpm, 21ºC) and extracted with ethyl acetate (2 x 500 µL), dried with Na_2SO_4 and measured by GC-FID (low boiler method). Reactions were done in duplicates and the average is reported. Conversions were stated based on area percentages of the formed ketones.
[b] The E-value determined at a conversion closest to 50% is given, since the error is expected here to be smallest.

d) Cosolvent study of PQQ-DH whole cells in the oxidation of 1a

Substrate *rac*-**1a** was used as the model substrate for the cosolvent study. Various cosolvents with different ratios, 5% v/v to 50% v/v, were tested in the oxidation of *rac*-**1a** (Table S6, Fig S2).

Table S6. Cosolvent study of PQQ-DH whole cells in the oxidation of substrate *rac*-**1a**

	Conv. [%]				
**Cosolvents**	**5% v/v**	**10% v/v**	**20% v/v**	**30% v/v**	**50% v/v**
DMSO	>99	>99	>99	68	n.d.[b]
Isooctane	98	98	97	>99	99
Glycerol	87	90	83	69	47
n-Heptane	>99	>99	96	98	89
MeOH	98	86	41	n.d.[b]	n.d.[b]
EtOH	65	41	n.d.[b]	n.d.[b]	n.d.[b]
*i*PrOH	22	8	0.3	n.d.[b]	n.d.[b]
1-Hexanol	1	0.6	0.5	n.d.[b]	n.d.[b]
2-Butanone	41	0.3	n.d.[b]	n.d.[b]	n.d.[b]
Acetone	62	14	0.4	n.d.[b]	n.d.[b]
DMF	95	61	0.3	n.d.[b]	n.d.[b]
Dioxane	83	50	0.1	0.1	n.d.[b]
CH_2Cl_2	1	2	3	n.d.[b]	n.d.[b]
EtOAC	61	0.6	n.d.[b]	n.d.[b]	n.d.[b]

[a] Condition: Tris-HCl (100 mM, pH 7.5) containing the lyophilized PQQ-DH cells (20 mg/mL in 1 mL reaction volume), PQQ (100 µM final concentration), potassium ferricyanide (PFC, 20 mM), the substrate (10 mM final concentration) with 5 to 50% v/v of various cosolvents. The reaction mixtures and blanks were shaken overnight (170 rpm, 21ºC) and extracted with ethyl acetate (2 x 500 µL), dried with Na_2SO_4 and measured by GC-FID (low boiler method). Reactions were done in duplicates and the average is reported. Conversions were stated based on area percentages of the formed ketones.
[b] No conversion was determined.

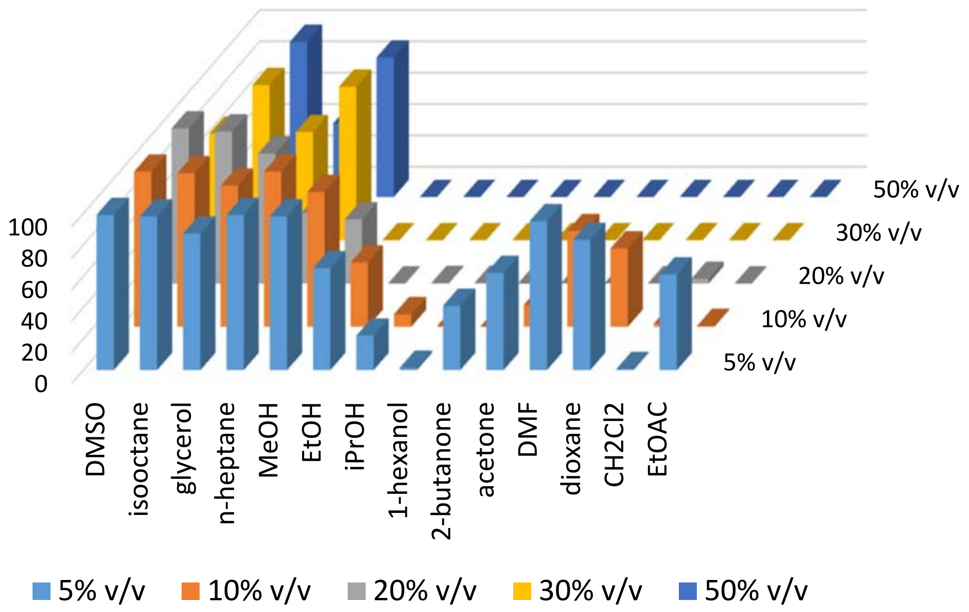

Figure S2. Cosolvent study using 20 mg of PQQ-DH whole cells in the oxidation of **1a**

Some samples from the cosolvent study were subjected to chiral analytics (Table S7). The E values varies from 3 in case of using 5% v/v of 2-butanone till 77 in case of using 20% v/v of MeOH.

Table S7. Enantioselectivity of reactions with cosolvent using PQQ-DH (20 mg/mL) in the oxidation of **1a**

Cosolvents	v/v [%]	Conv. [%]	*ee*$_S$ [%]	E
DMSO	30	68	n.a. [b]	n.a. [b]
Glycerol	5	87	>98	n.d. [c]
n-Heptane	50	89	>98	n.d. [c]
	10	86	>98	n.d. [c]
MeOH	20	41	66	77
	5	65	>98	16
EtOH	10	41	62	33
*i*PrOH	5	22	26	5
2-Butanone	5	41	60	3
	5	62	>98	20
Acetone	10	14	16	8
DMF	10	61	>98	22
	5	83	>98	n.d. [c]
Dioxane	10	50	78	19
EtOAC	5	61	92	12

[a] Condition: Tris-HCl (100 mM, pH 7.5) containing the lyophilized PQQ-DH cells (20 mg/mL in 1 mL reaction volume), PQQ (100 µM final concentration), potassium ferricyanide (PFC, 20 mM), the substrate (10 mM final concentration) with 5 to 50% v/v of various cosolvents. The reaction mixtures and blanks were shaken overnight (170 rpm, 21 ºC) and extracted with ethyl acetate (2 x 500 µL), dried with Na_2SO_4 and measured by GC-FID (low boiler method). Reactions were done in duplicates and the average is reported. Conversions were stated based on area percentages of the formed ketones.
[b] Data are not available.
[c] Not determined due to the inappropriate level conversion to perform the calculation in a reliable fashion.

e) Testing the effect of different buffers in the oxidation step using PQQ-DH whole cells

According to the obtained data from cosolvent study and the solubility of the substrates in different cosolvents, DMSO was chosen as the preferred cosolvent for the cascade. In the next step the effect of buffer composition and pH was investigated. For that purpose, the oxidation of *rac*-**1a-4a** (10 mM) was performed in two different buffers (KPi, 200 mM, pH 7.0 and Tris-HCl 100 mM, pH 7.5). Higher

conversions were obtained, when KPi was used as the reaction media (Table S8). Afterwards the effect of the pH of KPi buffer was checked after 24 h and 48 h reaction time (Table S8). Results revealed that increasing the pH to 8.0 led to a drop in the conversions but increasing the reaction time to 48 h in pH 7.0 led to higher conversions.

Considering all the results from different experiments in the oxidation step, the best condition was concluded to be using 20 mg/mL of PQQ-DH whole cells in the presence of 2% v/v DMSO as cosolvent in the KPi buffer (200 mM, pH 7.0) and running the reactions for 48 h.

Table S8. Oxidation of *rac*-**1a**-**4a** using PQQ-DH in KPi at pH 7.0 and pH 8.0 and Tris-HCl at pH 7.5 after 24 and 48 h reaction time [a]

**Sub.**	**Conv. [%]**					
	**KPi (200 mM, pH 7.0)**		**KPi (200 mM, pH 8.0)**		**Tris-HCl (100 mM, pH 7.5)**	
	24 h	48 h	24 h	48 h	24 h	48 h
***rac*-1a**	98	>99	81	97	>99	>99
***rac*-2a**	73	97	79	70	64	86
***rac*-3a**	93	>99	83	80	89	>99
***rac*-4a**	90	94	78	62	87	90

[a] Condition: The buffer (KPi, 200 mM, pH 7.0 or 8.0 or Tris-HCl 100 mM, pH 7.5) containing the lyophilized PQQ-DH cells (20 mg/mL in 1 mL reaction volume in 4 mL glass vials), PQQ (100 µM), potassium ferricyanide (PFC, 20 mM), the substrate (10 mM) with 2% v/v DMSO as cosolvent. The reaction mixtures and blanks were shaken horizontally for 24 and 48 hours (170 rpm, 21 ºC) and extracted with ethyl acetate (2 x 500 µL), dried with Na_2SO_4 and measured by GC-FID (low boiler method). Conversions were stated based on area percentages of the formed ketones.

2.2. Amination step using various ω-TAs and amine donors

2.2.1. Amination of 1b-4b using ArRmut11-ωTA and various amine donors

Different amine donors including isopropylamine (**5c**), (*R*)-1-phenylethylamine ((*R*)-**6c**) and 1,2-diaminocyclohexane (0.5-2.5 M final concentration in 1 mL reaction volume) were tested in the amination of **1b-4b** (50 mM final concentration). All reactions were done in triplicates. Results from these experiments are shown in Table S9 and Figures S3-S6.

Table S9. Amination of **1b**-**4b** (50 mM) in buffer (KPi, 200 mM, pH 8.0) employing ArRmut11 ω-TA and various amine donors with different concentrations at 40 ºC for 24 h [a]

**Amine donor**	Isopropylamine (**5c**) **Conv. [%]**				*all-rac*-1,2-Diaminocyclohexane **Conv. [%]**				(*R*)-1-Phenylethylamine ((*R*)-**6c**) **Conv. [%]**			
**Amine donor conc.** / **Sub.**	0.5 M	1 M	1.25 M	2.5 M	0.5 M	1 M	1.25 M	2.5 M	0.5 M	1 M	1.25 M	2.5 M
**1b**	26	38	43	51	62	73	78	84	82	93	93	20
**2b**	20	34	36	44	64	70	77	78	79	87	90	n.d.[b]
**3b**	17	27	29	39	45	57	65	65	71	73	78	n.d.[b]
**4b**	4	6	6	9	14	17	25	27	44	47	55	7

[a] Condition: KPi (200 mM, pH 8.0) containing substrate (50 mM in 1 mL reaction volume), 2% v/v DMSO, amine donors (0.5 M-2.5 M), PLP (1 mM), rehydrated lyophilized cells (40 mg/mL) were mixed in 2 mL eppis. The biotransformation samples were incubated overnight (450 rpm, horizontal shaking, 40 ºC). After basifying, the reactions were extracted with ethyl acetate (3 x 500 µL), dried with Na_2SO_4 and measured by GC-FID. Conversions were stated based on area percentages of the formed amines.
[b] No conversion was detected.

Amination of 1b

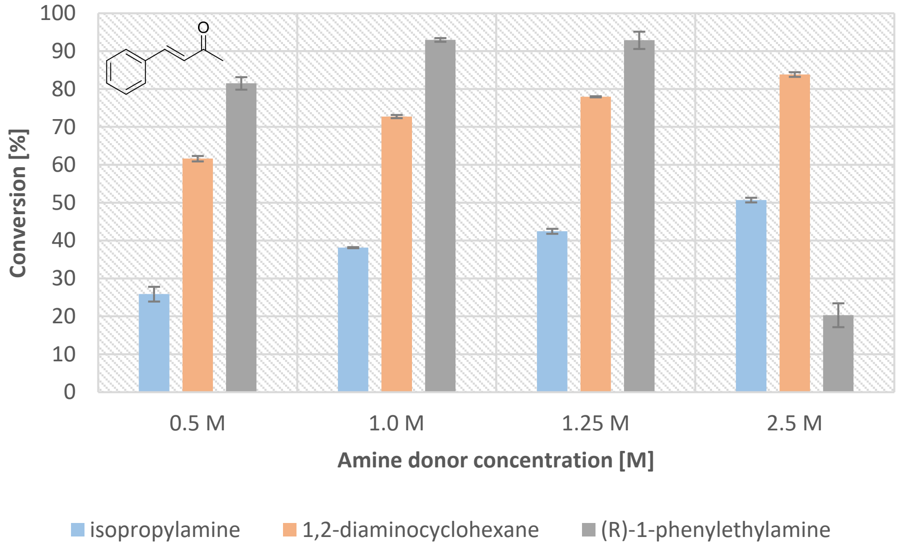

Figure S3. Amination of **1b** (50 mM, 10% v/v DMSO) in the buffer (KPi, 200 mM, pH 8.0) employing ArRmut11 and various amine donors with different concentrations at 40 ºC for 24 h

Results show that the highest conversion of **1b** (93%, Figure S3) to the corresponding amine **1c** was obtained by using (*R*)-1-phenylethylamine ((*R*)-**6c**, 1.25 M) as the amine donor. With increasing the concentration of the donor to 2.5 M, a drop in the conversion was observed to 20%. After (*R*)-1-phenylethylamine ((*R*)-**6c**), high conversions were obtained by using 1,2-diaminocyclohexane as the amine donor (84% conversion, 2.5 M amine donor). Lower conversions were obtained by using isopropylamine (**5c**) compared to the other amine donors. With increasing the concentration of the amine donor, higher conversions were obtained (51% conversion, 2.5 M donor).

Amination of 2b

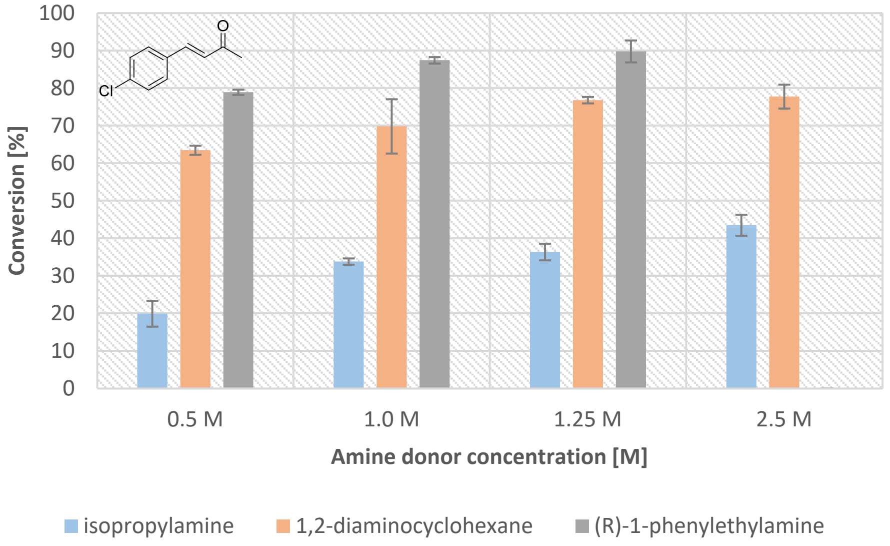

Figure S4. Amination of ketone **2b** (50 mM) in the buffer (KPi, 200 mM, pH 8.0) employing ArRmut11 and various amine donors with different concentrations at 40 ºC for 24 h

The results from the amination of **2b** to its corresponding amine **2c** are shown in Figure S4. The highest conversion (90%) was obtained using (*R*)-1-phenylethylamine ((*R*)-**6c**, 1.25 M) as the amine donor. By using 1,2-diaminocyclohexane (0.5 M) as an amine donor, 63% conversion was observed. Increasing the concentration of the donor led to higher conversions (78% conversion, 2.5 M donor). Lower conversions were obtained using isopropylamine (**5c**) compared to the other amine donors. With increasing the concentration of isopropylamine (**5c**), higher conversions were obtained (44% conversion, 2.5 M donor).

Amination of 3b

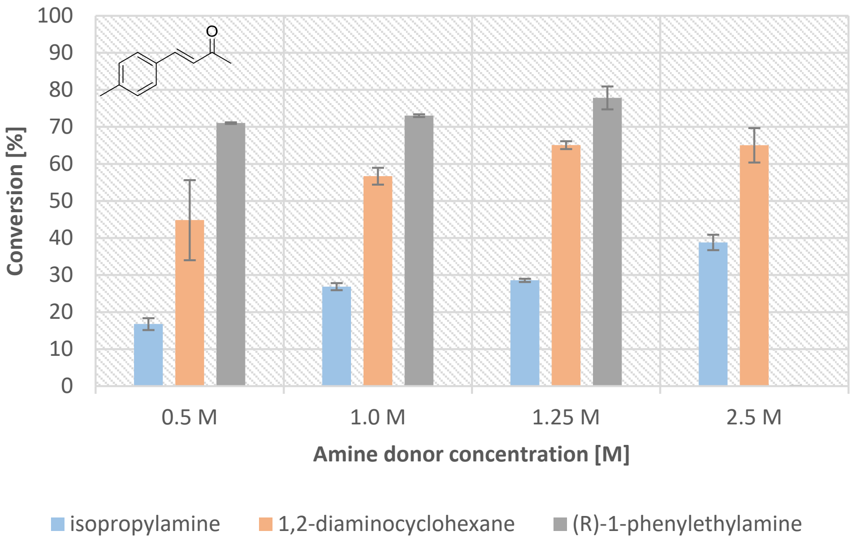

Figure S5. Amination of **3b** (50 mM, 10% v/v DMSO) in the buffer (KPi, 200 mM, pH 8.0) employing ArRmut11 and various amine donors with different concentrations at 40 ºC for 24 h

Figure S5 shows the results from the amination of **3b** to its corresponding amine **3c**. The highest conversion (78%) was obtained using (*R*)-1-phenylethylamine ((*R*)-**6c**, 1.25 M) as the amine donor. By increasing the concentration of the donor, the conversion increased (up to 1.25 M), but at 2.5 M no conversion was observed. After (*R*)-1-phenylethylamine ((*R*)-**6c**), the highest conversion (65%) was obtained by 1,2-diaminocyclohexane. Increasing the concentration of the donor led to higher conversions. Lower conversions were obtained by using isopropylamine (**5c**) compared to the other donors. With increasing the concentration of the amine donor, higher conversions were obtained (39% conversion, 2.5 M donor).

Amination of 4b

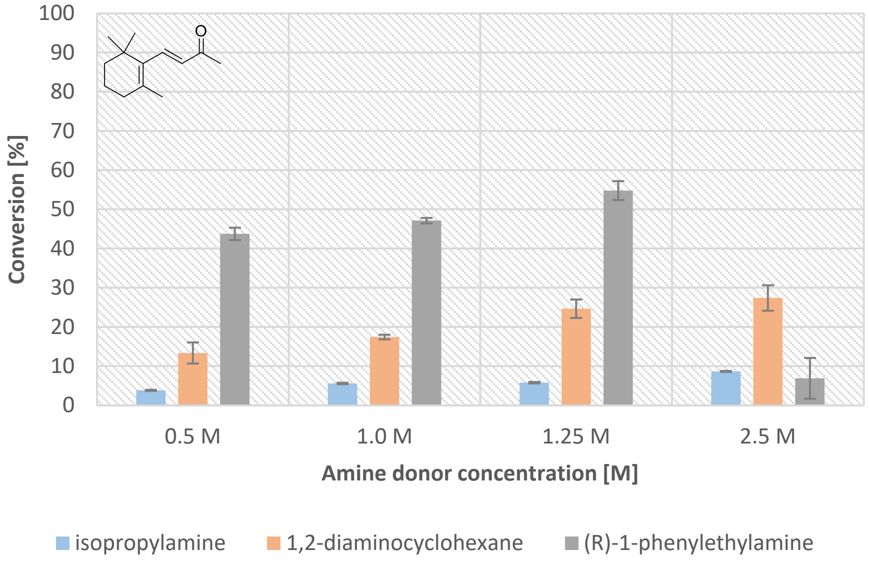

Figure S6. Amination of **4b** (50 mM, 10% v/v DMSO) in the buffer (KPi, 200 mM, pH 8.0) employing ArRmut11 and various amine donors with different concentrations at 40 ºC for 24 h

Figure S6 shows the results from the amination of **4b** to its corresponding amine **4c**. Lower conversions were obtained for this substrate compared to the other substrates. The highest conversion (55%) was obtained using (*R*)-1-phenylethylamine ((*R*)-**6c**), 1.25 M) as the amine donor. By increasing the concentration of the donor, the conversion increased but at 2.5 M no conversion was observed. After (*R*)-1-phenylethylamine ((*R*)-**6c**), the highest conversion (27%) was obtained by 1,2-diaminocyclohexane (2.5 M). Increasing the concentration of the donor led to higher conversions. Lower conversions were obtained by using isopropylamine (**5c**), compared to the other amine donors. With increasing the concentration of the amine donor, higher conversions were obtained (9% conversion by using 2.5 M isopropylamine (**5c**)).

2.2.2. Amination of 1b-4b using ArRmut11-ωTA with (R)-1-phenylethylamine as the amine donor at different pH levels

The best amine donor (1.25 M, (*R*)-1-phenylethylamine ((*R*)-**6c**)) was chosen to screen the effect of the pH on the amination of various substrates using ArRmut11-ωTA. Results showed that in all cases, the increment of the pH led to lower conversion levels (Figure S7). The highest conversion (93%) was obtained in the amination of **1b** at pH 8.0. The conversions 90% and 82% were obtained in the amination of substrate **2b** and **3b**, respectively. β-Ionone (**4b**) was less accepted by the enzyme since 46% conversion was the highest observed conversion in the tested conditions.

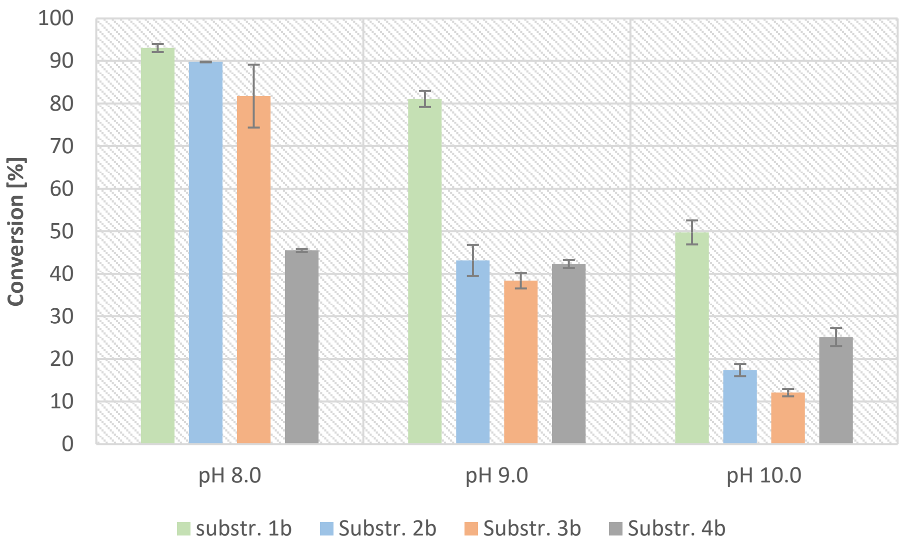

Figure S7. Amination of **1b**-**4b** (50 mM, 10% v/v DMSO) in the buffer (KPi, 200 mM, pH 8, 9 and 10) employing ArRmut11 and (*R*)-1-phenylethylamine ((*R*)-**6c**) with different concentrations at 40 ºC for 24 h

2.2.3. Amination of 1b using various ω-TAs

a) Isopropylamine (**5c**) *as the amine donor*

Various transaminases as it was mentioned before (in section 1.3.1) were screened in the amination of **1b** (10 mM) as the model substrate using isopropylamine (**5c**) as the amine donor (Table S10). Results show that the highest conversions were obtained when pEG 205 and 209 were employed using 2.5 M isopropylamine (**5c**) (77% and 71%, respectively).

Table S10. Amination of **1b** using various ω-TAs and isopropylamine (**5c**) with different concentrations [a]

Source of TA	pEG number (internal number)	Selectivity	Conversion [%]				
			300 mM	500 mM	1 M	2 M	2.5 M
Alcaligenes denitrifcans	19	(*S*)	n.d. [b]	n.a. [c]	n.a.	n.a.	n.a.
Paracoccus denitrificans	24	(*S*)	5	n.a.	n.a.	n.a.	n.a.
Pseudomonas putida	25	(*S*)	3	n.a.	n.a.	n.a.	n.a.
Alcaligenes denitrificans	30	(*S*)	4	n.a.	n.a.	n.a.	n.a.
Arthrobacter aurescens	70	(*R*)	n.d.	n.a.	n.a.	n.a.	n.a.
Giberella zeae	202	(*R*)	n.d.	n.a.	n.a.	n.a.	n.a.
Ochrobactrum anthropi	204	(*S*)	24	21	15	7	5
Silicibacter pomeroyi	205	(*S*)	30	40	56	74	77
Vibrio fluvialis	209	(*S*)	32	43	56	56	71
Arthrobacter sp.	23	(*R*)	19	27	36	11	11
Arthrobacter citreus	29	(*S*)	36	37	33	n.d.	n.d.
Bacillus megaterium	31	(*S*)	30	36	50	16	6
Aspergillus tereus	97	(*R*)	27	34	36	9	9
Hyphomonas neptunium	98	(*R*)	15	22	27	19	6
Pseudomonas fluorescens	148	(*S*)	8	n.a.	n.a.	n.a.	n.a.
Neosartorya fischeri	203	(*R*)	12	16	22	22	15

[a] Condition: KPi (200 mM, pH 8.0) containing substrate (10 mM in 1 mL reaction volume), 2% v/v DMSO, isopropylamine (300 mM-2.5 M), PLP (1 mM), rehydrated lyophilized cells (40 mg/mL) were mixed in 2 mL eppis. The biotransformation samples were incubated overnight (350 rpm, horizontal shaking, 30 ºC). Conversions were stated based on area percentages of the formed amines.
[b] No conversion was detected.
[c] Data are not available.

After finding the best conditions in terms of amine donor concentration (2.5 M for pEG 205 and 209 and 1 M for other tested transaminases), the amination of other substrates was tested using isopropylamine as the amine donor as well. Results show that the best conversions were obtained using pEG 205 and 209 in the case of other tested substrates as well (Table S11).

Table S11. Amination of different substrates using various ω-TAs and 1 M and 2.5 M isopropylamine (**5c**) [a]

pEG number	Source	Selectivity	Amine donor conc.	Conversion [%]			
				**1b**	**2b**	**3b**	**4b**
**pEG 204**	*Ochrobactrum anthropi*	(*S*)	1 M	15	2	4	n.d. [b]
**pEG 205**	*Silicibacter pomeroyi*	(*S*)	2.5 M	77	67	57	27
**pEG 209**	*Vibrio fluvialis*	(*S*)	2.5 M	71	46	50	<1
**pEG 23**	*Arthrobacter* sp.	(*R*)	1 M	36	18	24	5
**pEG 31**	*Bacillus megaterium*	(*S*)	1 M	50	18	18	4
**pEG 97**	*Aspergillus tereus*	(*R*)	1 M	36	11	6	1
**pEG 98**	*Hyphomonas neptunium*	(*R*)	1 M	27	14	13	2
**pEG 203**	*Neosartorya fischeri*	(*R*)	1 M	22	10	13	3

[a] Condition: KPi (200 mM, pH 8.0) containing substrate (10 mM in 1 mL reaction volume), 2% v/v DMSO, isopropylamine (1 M and 2.5 M), PLP (1 mM), rehydrated lyophilized cells (40 mg/mL) were mixed in 2 mL eppis. The biotransformation samples were incubated overnight (350 rpm, horizontal shaking, 30 ºC). Conversions were stated based on area percentages of the formed amines.
[b] No conversion was detected.

b) 1-Phenylethylamine as the amine donor ((*R*)-6c)

(*R*)-1-Phenylethylamine ((*R*)-**6c**) was tested in the amination of **1b**, using (*R*)-selective transaminases. By using 500 mM of amine donor and ω-TA pEG 23, 96% of conversion was obtained. After that, the highest conversion (77%) was obtained using pEG 203 and 300 mM isopropylamine (**5c**) (Table S12).

Table S12. Amination of **1b** using various ω-TAs and (*R*)-1-phenylethylamine ((*R*)-**6c**) with different concentrations [a]

pEG number	Source	Selectivity	Conversion [%]		
			300 mM	500 mM	1 M
**pEG 70**	*Arthrobacter aurescens*	(*R*)	4	n.a.	n.a.
**pEG 202**	*Giberella zeae*	(*R*)	3	n.a.	n.a.
**pEG 23**	*Arthrobacter* sp.	(*R*)	90	96	20
**pEG 97**	*Aspergillus tereus*	(*R*)	63	2	n.d. [b]
**pEG 98**	*Hyphomonas neptunium*	(*R*)	2	n.a. [c]	n.a.
**pEG 203**	*Neosartorya fischeri*	(*R*)	77	19	n.d.

[a] Condition: KPi (200 mM, pH 8.0) containing substrate (10 mM in 1 mL reaction volume), 2% v/v DMSO, isopropylamine (300 mM-1 M), PLP (1 mM), rehydrated lyophilized cells (40 mg/mL) were mixed in 2 mL eppis. The biotransformation samples were incubated overnight (350 rpm, horizontal shaking, 30 ºC). Conversions were stated based on area percentages of the formed amines.
[b] No conversion was detected.
[c] Data are not available.

(*S*)-Selective transaminases were screened in the amination of **1b** using (*S*)-1-phenylethylamine ((*S*)-**6c**) as the amine donor (Table S13).

Table S13. Amination of **1b** using various ω-TAs and (*S*)-1-phenylethylamine ((*S*)-**6c**) with different concentrations [a]

pEG number	Source	Selectivity	Conversion [%]			
			100 mM	300 mM	500 mM	1 M
**pEG 19**	*Alcaligenes denitrifcans*	(*S*)	n.d. [b]	n.d.	n.d.	n.d.
**pEG 24**	*Paracoccus denitrificans*	(*S*)	n.d.	n.d.	n.d.	n.d.
**pEG 25**	*Pseudomonas putida*	(*S*)	n.d.	n.d.	n.d.	n.d.
**pEG 30**	*Alcaligenes denitrificans*	(*S*)	n.d.	n.d.	n.d.	n.d.
**pEG 204**	*Ochrobactrum anthropi*	(*S*)	n.d.	n.d.	n.d.	n.d.
**pEG 29**	*Arthrobacter citreus*	(*S*)	n.d.	n.d.	n.d.	n.d.
**pEG 31**	*Bacillus megaterium*	(*S*)	33	n.d.	n.d.	n.d.
**pEG 148**	*Pseudomonas fluorescens*	(*S*)	35	10	n.d.	n.d.

[a] Condition: KPi (200 mM, pH 8.0) containing substrate (10 mM in 1 mL reaction volume), 2% v/v DMSO, (*S*)-1-phenylethylamine (300 mM-2.5 M), PLP (1 mM), rehydrated lyophilized cells (40 mg/mL) were mixed in 2 mL eppis. The biotransformation samples were incubated overnight (350 rpm, horizontal shaking, 30 ºC). Conversions were stated based on area percentages of the formed amines.
Note: Some of these data are shown in the main paper and for a better overview are repeated here.
[b] No conversion was detected.

2.2.4. Enantiomeric excess measurements

The samples were measured on HPLC equipped with OD-H and OJ columns. In all cases, the formation of enantiomerically pure amines was confirmed (Tables S14).

Table S14. Stereochemical outcome of amination of various substrates using different conditions

Sub.	Sub. conc.	ω-TA	Amine donor	Amine configuration	pH	Conv. [%] (*ee*$_p$ [%])
**1b** [a]	10 mM	pEG 26	(*R*)-1-phenylethylamine	(*R*)	8.0	90 (>98)
**1b** [a]	10 mM	pEG 29	(*R*)-1-phenylethylamine	(*R*)	8.0	63 (>98)
**1b** [a]	10 mM	pEG 32	(*R*)-1-phenylethylamine	(*R*)	8.0	77 (>98)
**1b** [a]	10 mM	pEG 205	isopropylamine	(*S*)	8.0	56 (>98)
**1b** [a]	10 mM	pEG 209	isopropylamine	(*S*)	8.0	56 (>98)
**1b** [a]	10 mM	pEG 31	isopropylamine	(*S*)	8.0	50 (>98)
**1b** [a]	10 mM	pEG 97	isopropylamine	(*R*)	8.0	36 (>98)
**1b** [a]	10 mM	pEG 23	isopropylamine	(*R*)	8.0	96 (>98)
**1b** [a]	10 mM	pEG 205	isopropylamine	(*S*)	8.0	77 (>98)
**1b** [a]	10 mM	pEG 209	isopropylamine	(*S*)	8.0	71 (>98)
**3b** [a]	10 mM	pEG 205	isopropylamine	(*S*)	8.0	57 (>98)
**3b** [a]	10 mM	pEG 209	isopropylamine	(*S*)	8.0	50 (>98)
**2b** [b]	10 mM	pEG 205	isopropylamine	(*S*)	8.0	67 (>98)
**2b** [b]	10 mM	pEG 209	isopropylamine	(*S*)	8.0	46 (>98)
**4b** [b]	10 mM	pEG 205	isopropylamine	(*S*)	8.0	27 (>98)
**1b** [a]	10 mM	pEG 148	(*S*)-1-phenylethylamine	(*S*)	8.0	10 (>98)
**1b** [a]	10 mM	pEG 209	(*S*)-1-phenylethylamine	(*S*)	8.0	12 (>98)
**1b** [a]	10 mM	pEG 205	(*S*)-1-phenylethylamine	(*S*)	8.0	87 (>98)
**1b** [a]	50 mM	pEG 90	(*R*)-1-phenylethylamine	(*R*)	8.0	94 (>98)
**1b** [a]	50 mM	pEG 90	(*R*)-1-phenylethylamine	(*R*)	9.0	83 (>98)
**1b** [a]	50 mM	pEG 90	(*R*)-1-phenylethylamine	(*R*)	10.0	53 (>98)
**3b** [a]	50 mM	pEG 90	(*R*)-1-phenylethylamine	(*R*)	8.0	90 (>98)
**3b** [a]	50 mM	pEG 90	(*R*)-1-phenylethylamine	(*R*)	9.0	43 (>98)
**3b** [a]	50 mM	pEG 90	(*R*)-1-phenylethylamine	(*R*)	10.0	13 (>98)
**2b** [b]	50 mM	pEG 90	(*R*)-1-phenylethylamine	(*R*)	8.0	91 (>98)
**2b** [b]	50 mM	pEG 90	(*R*)-1-phenylethylamine	(*R*)	9.0	46 (>98)
**2b** [b]	50 mM	pEG 90	(*R*)-1-phenylethylamine	(*R*)	10.0	19 (>98)
**4b** [b]	50 mM	pEG 90	(*R*)-1-phenylethylamine	(*R*)	8.0	46 (>98)
**4b** [b]	50 mM	pEG 90	(*R*)-1-phenylethylamine	(*R*)	9.0	43 (>98)
**4b** [b]	50 mM	pEG 90	(*R*)-1-phenylethylamine	(*R*)	10.0	28 (>98)

[a] Condition: OD-H column (Method for **1b** and **3b**: *n*-Hep: *i*PrOH 90:10, Flow: 1.0 mL/min, 245 nm, Temperature: 25 ºC).
[b] Condition: OJ column (Method for **2b**: *n*-Hep: *i*PrOH 90:10, Flow: 0.7 mL/min, 254 nm, Temperature: 25 ºC, Method for **4b**: *n*-Hep: *i*PrOH 99:1, Flow: 0.7 mL/min, 230 nm, Temperature: 25 ºC). Conversions were stated based on area percentages of the formed amines.
Note: Some of these data are shown in the main paper and for a better overview are repeated here.

2.3. One-pot two-step cascade

All substrates (*rac*-**1a**-**4a**) were subjected to the cascade by using 20 mg/mL of PQQ-DH lyophilized cells for the oxidation step and 40 mg/mL of ArRmut11 for the amination step. Control reactions including samples containing only PQQ-DH lyophilized cells (for measuring the conversion to ketone), or ω-TA ArRmut11 lyophilized cells (for measuring the conversion to amine) were run in parallel to blank reactions without any cells.

When ArRmut11 (pEG 90) and (*R*)-1-phenylethylamine (*R*)-**6c** were used in the cascade, in the case of *rac*-**1a**-**3a**, full conversions were obtained, in contrast to using isopropylamine **5c** which led to lower conversions (Table S15). In the case of (*S*)-selective ω-TA in the combination with (*S*)-1-phenylethylamine (*S*)-**6c**, ω-TA from *Silicibacter pomeroyi* (pEG 205) led to higher conversions (Table S16) compared to when ω-TA from *Vibrio fluvialis* (pEG 209) was used (Table S17).

Table S15. Asymmetric amination of alcohols *rac*-**1a**-**4a** using PQQ-DH and ArRmut11 TA with (*R*)-1-phenylethylamine (*R*)-**6c** or isopropylamine **5c** as amine donors[a]

Sub.	Amine donor conc.	Amine donor	Single step experiment alcohol to ketone [%] after 48 h[c]	Single step experiment ketone to amine [%] [d]	Cascade alc. to amine [%]	*ee*$_P$ [%]
***rac*-1a**	1.25 M	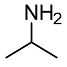	>99	51	43	>98 (*R*)
	250 mM	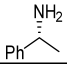		>99	>99	>98 (*R*)
***rac*-2a**	1.25 M	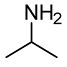	>99	50	47	>98 (*R*)
	250 mM	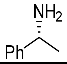		>99	>99	>98 (*R*)
***rac*-3a**	1.25 M	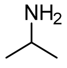	>99	41	42	>98 (*R*)
	250 mM	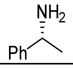		>99	>99	>98 (*R*)
***rac*-4a**	1.25 M	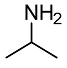	>99	23	18	>98 (*R*)
	250 mM	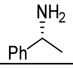		62	57	>98 (*R*)

[a] Condition: KPi (200 mM, pH 7.0 for oxidation step, 8.0 for amination step) containing substrate (10 mM), DMSO (2% v/v), PQQ-DH (20 mg/mL), PQQ (100 μM), potassium ferricyanide (PFC, 20 mM) for the oxidation step, and ω-TA (40 mg/mL), (*R*)-1-phenylethylamine (250 mM) or isopropylamine (1.25 mM) and PLP (1 mM) for amination step were mixed in 4 mL glass vial (final reaction volume: 1.5 mL). The oxidation step was run 48 h (170 rpm, horizontal shaking, 21 °C), after addition of ω-TA and the amine donor, samples were incubated another 24 h (350 rpm, horizontal shaking, 40 °C). Conversion was measured by GC-MS based on the area percentages of the amines formed.
Note: Some of these data are shown in the main paper and for a better overview are repeated here.
[b] Conversion for the oxidation of the alcohol to ketone, performed in a separate experiment.
[c] Conversion for the amination of ketone to the amine, performed in a separate experiment.

Table S16. Asymmetric amination of alcohols **1a**-**4a** using PQQ-DH and TA from *Silicibacter pomeroyi* with (*S*)-1-phenylethylamine ((*S*)-**6c**) or isopropylamine (**5c**) as amine donors[a]

Sub.	Amine donor conc.	Amine donor	Single step experiment alcohol to ketone [%] after 48 h[c]	Single step experiment ketone to amine [%] [d]	Cascade alc. to amine [%]	*ee*$_P$ [%]
rac-**1a**	1.25 M	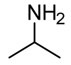	>99	48	38	>98 (*S*)
	300 mM	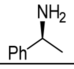		95	99	>98 (*S*)
rac-**2a**	1.25 M	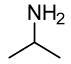	>99	53	43	>98 (*S*)
	300 mM	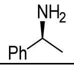		89	96	>98 (*S*)
rac-**3a**	1.25 M	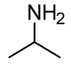	>99	35	35	>98 (*S*)
	300 mM	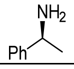		76	92	>98 (*S*)
rac-**4a**	1.25 M	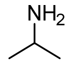	>99	22	18	>98 (*S*)
	300 mM	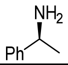		24	66	>98 (*S*)

[a] Condition: KPi (200 mM, pH 7.0 for oxidation step, 8.0 for amination step) containing substrate (10 mM), DMSO (2% v/v), PQQ-DH (20 mg/mL), PQQ (100 µM), potassium ferricyanide (PFC, 20 mM) for the oxidation step, and ω-TA (40 mg/mL), (*S*)-1-phenylethylamine (300 mM) or isopropylamine (1.25 mM) and PLP (1 mM) for amination step were mixed in 4 mL glass vial (final reaction volume: 1.5 mL). The oxidation step was run 48 h (170 rpm, horizontal shaking, 21 ºC), after addition of ω-TA and the amine donor, the samples were incubated another 24 h (350 rpm, horizontal shaking, 30 ºC). Conversion was measured by GC-MS based on the area percentages of the amines formed. Note: Some of these data are shown in the main paper and for a better overview are repeated here.
[b] Conversion for the oxidation of the alcohol to ketone, performed in a separate experiment.
[c] Conversion for the amination of ketone to the amine, performed in a separate experiment.

Table S17. Asymmetric amination of alcohols **1a**-**4a** using PQQ-DH and the TA from *Vibrio fluvialis* with (*S*)-1-phenylethylamine ((*S*)-**6c**) or isopropylamine (**5c**) as amine donors[a]

Sub.	Amine donor conc.	Amine donor	Single step experiment alcohol to ketone [%] after 48 h[c]	Single step experiment ketone to amine [%] [d]	Cascade Alc. to amine [%]	*ee*$_P$ [%]
rac-**1a**	1.25 M	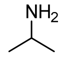	>99	42	31	>98 (*S*)
	300 mM	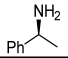	>99	66	75	>98 (*S*)
rac-**2a**	1.25 M	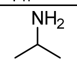	>99	47	40	>98 (*S*)
	300 mM	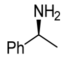	>99	70	77	>98 (*S*)
rac-**3a**	1.25 M	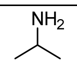	>99	40	26	>98 (*S*)
	300 mM	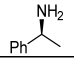	>99	58	59	>98 (*S*)
rac-**4a**	1.25 M	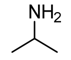	>99	n.d. [b]	n.d. [b]	>98 (*S*)
	300 mM	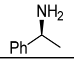	>99	n.d. [b]	n.d. [b]	>98 (*S*)

[a] Condition: Condition: KPi (200 mM, pH 7.0 for oxidation step, 8.0 for amination step) containing substrate (10 mM), DMSO (2% v/v), PQQ-DH (20 mg/mL), PQQ (100 µM), potassium ferricyanide (PFC, 20 mM) for the oxidation step, and ω-TA (40 mg/mL), (*S*)-1-phenylethylamine (300 mM) or isopropylamine (1.25 M) and PLP (1 mM) for the amination step were mixed in 4 mL glass vial (final reaction volume: 1.5 mL).

The oxidation step was run for 48 h (170 rpm, horizontal shaking, 21 ºC), then after addition of ω-TA and amine donor, samples were incubated another 24 h (350 rpm, horizontal shaking, 30 ºC). Conversion was measured by GC-MS based on the area percentages of the amines formed.
[b] Data are not available.
[b] Conversion for the oxidation of the alcohol to ketone, performed in a separate experiment.
[c] Conversion for the amination of ketone to the amine, performed in a separate experiment.

2.4. Amination cascade on 0.2 mmol scale

For obtaining semi-preparative amounts of optically pure (*R*)-amines **1c**-**3c**, experiments were performed with 0.2 mmol at 10 mM substrate concentration employing 20 mg/mL of PQQ-DH lyophilized cells for the oxidation step and 40 mg/mL of ArRmut11 for the amination step (Table S18). After purification, the isolated yields were determined, and NMR analysis proved the structure and the purity of the isolated (*R*)-amines **1c**-**3c**.

Table S18. Amination cascade for *rac*-**1a**-**3a** (10 mM) on 0.2 mmol scale using PQQ-DH and ArRmut11 with **5c** as the amine donor [a]

**Sub.**	**Composition [%]**	***eeamine* [%]**	**Isolated yield [b]**	**Optical rotation** $[\alpha]^{20}_{D}$
***rac*-1a**	55% (amine), 45% (ketone)	97 (*R*)	30% (4.9 mg)	+22.5<br>c = 0.4 g/100 mL in $CHCl_3$<br><br>Lit: +21.5<br>c = 1.0 g/100 mL in $CHCl_3$ (98% e.e.) [23]<br><br>Lit: -11.0 for (*S*)-**1c**<br>c = 1.0 g/100 mL, $CHCl_3$, (80.4% e.e.)[24]
***rac*-2a**	46% (amine), 8% (alcohol), 46% (ketone)	97 (*R*)	25% (4.1 mg)	+18.0<br>c = 0.1 g/100 mL in $CHCl_3$
***rac*-3a**	34% (amine), 3% (alcohol), 63% (ketone)	97 (*R*)	41% (4.6 mg)	+36.1<br>c = 0.5 g/100 mL in $CHCl_3$

[a] Condition: KPi (200 mM, pH 7.0 for oxidation step, 8.0 for amination step) containing the substrate (10 mM), DMSO (2% v/v), PQQ-DH (20 mg/mL) for the oxidation step, and ω-TA (40 mg/mL), 2-propylamine (**5c**, 1.25 M) and PLP (1 mM) for the amination step were mixed in 50 mL falcon tubes (final reaction volume: 20 mL). The oxidation step was run 48 h (170 rpm, horizontal shaking, 21 ºC), and after the addition of ω-TA and the amine donor, the samples were incubated another 24 h (350 rpm, horizontal shaking, 40 ºC).
[b] Composition were measured based on area ratio obtained from GC-MS. The percentage of isolated yield refers to the conversion achieved.

3. Analytics

All the obtained substrates and the reference compounds were analyzed by ^{1}H NMR and ^{13}C NMR. ^{1}H and ^{13}C NMR spectra were recorded using a 300 and 75 MHz instrument, respectively. Chemical shifts (δ) are given in parts per million (ppm) relative to TMS (δ = 0 ppm) or to the residual solvent signal and coupling constants (*J*) are reported in Hertz (Hz). Thin-layer chromatography was carried out on silica gel 60 F254 plates and compounds were visualized by UV. Optical rotation was measured at 589 nm (Na line) on an Anton Paar MCP5100 unit. Sample concentrations are given in g per 100 mL.

GC and GC-MS:

The reference materials were used for co-injection on GC and GC-MS. GC-MS measurements were carried out on a 7890A GC System (Agilent Technologies, Santa Clara, CA, USA), equipped with a 5975C mass selective detector and an HP-5MS column (5% phenylmethylsiloxane, 30 m x 0.20 mm x 0.25 µm, J&W Scientific, Agilent Technologies) using He as the carrier gas. Injector temperature: 250 °C; split ratio: 90:1; Injection volume: 5 µL; Flow rate: 0.7 mL/min; Temperature program (low boilers method): 40 °C, hold time 2.0 min, 10 °C/min to 180 °C, hold time 1.0 min; Temperature program (standard method): 100 °C, hold time 0.5 min, 10 °C/min to 300 °C, hold time 0 min; EI mode, energy 70 eV, MS Source: 230 °C, MS Quadrupole: 150 °C. For GC, HP-5 column (30 m x 0.32 mm x 0.25 µm, J&W Scientific, Agilent Technologies) was used with He as the carrier gas. Injector temperature: 250 °C; Injection volume: 5 µL; Flow rate: 0.7 mL/min; Temperature program (Standard Method): 100 °C, hold time 0.5 min, 10 °C/min to 300 °C, hold time 0 min. The conversion of the allylic alcohols to their corresponding ketones in the oxidation step was measured using the low boiler method with GC/GC-MS and the conversion of the allylic ketones to their corresponding amines was measured using the standard method with GC/GC-MS. Retention times of allylic alcohols and their corresponding ketones and amines with two methods are shown in Table S19 (low boilers) and S20 (standard method) for GC and in Table S21 (low boiler) and S22 (standard method) for GC-MS.

Table S19. Retention times of allylic alcohols and corresponding ketones using HP-5 column (30 m x 0.32 mm x 0.25 µm, J&W Scientific, Agilent Technologies) using He as carrier gas [a]

Structure	X= OH alcohols	X= =O ketones
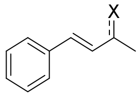	12.7	13.2
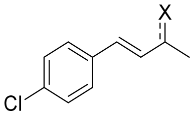	15.4	15.8
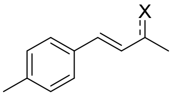	14.2	14.8
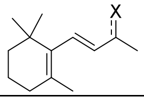	14.1	15.1

[a] Injector temperature: 250 °C; Injection volume: 5 µL; Flow rate: 0.7 mL/min; Temperature program (low boilers method): 40 °C, hold time 2.0 min, 10 °C/min to 180 °C, hold time 1.0 min.

Table S20. Retention times of allylic alcohols, ketones, amines, derivatized alcohols and amines by using acetic anhydride method, using HP-5 column (30 m x 0.32 mm x 0.25 µm, J&W Scientific, Agilent Technologies) using He as carrier gas [a]

Structure	Retention time [min]				
	**X= OH alcohols**	**X= =O ketones**	**X= OAc derivatized alcohols**	**X= NH_2 amines**	**X= NHOAc derivatized amines**
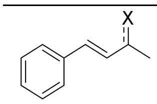	7.9	8.5	9.7	7.9	13.3
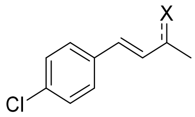	10.6	11.1	12.5	10.5	15.7
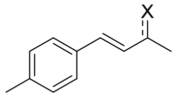	9.4	10.1	11.2	9.3	14.7
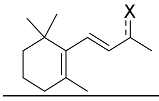	9.2	10.3	10.7	9.1	13.9

[a] Injector temperature: 250 °C; Injection volume: 5 µL; Flow rate: 0.7 mL/min; Temperature program (Standard Method): 100 °C, hold time 0.5 min, 10 °C/min to 300 °C, hold time 0 min.

Table S21. Retention times of allylic alcohols and corresponding ketones using HP-5MS column (5% phenylmethylsiloxane, 30 m x 0.20 mm x 0.25 µm, J&W Scientific, Agilent Technologies) using He as carrier gas [a]

Structure	X= OH alcohols	X= =O ketones
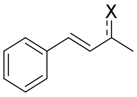	13.72	14.20
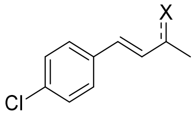	16.36	16.67
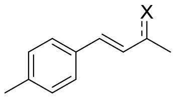	15.14	15.75
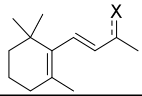	14.96	15.84

[a] Injector temperature: 250 °C; Injection volume: 1 µL; Flow rate: 0.7 mL/min; Temperature program (low boilers method): 40 °C, hold time 2.0 min, 10 °C/min to 180 °C, hold time 1.0 min; EI mode, energy 70 eV, MS Source: 230 °C, MS Quadrupole: 150 °C.

Table S22. Retention times of allylic alcohols, ketones, amines, derivatized alcohols and amines by using acetic anhydride method, using HP-5MS column (5% phenylmethylsiloxane, 30 m x 0.20 mm x 0.25 µm, J&W Scientific, Agilent Technologies) using He as carrier gas [a]

Structure	Retention time [min]				
	**X= OH alcohols**	**X= =O ketones**	**X= OAc derivatized alcohols**	**X= NH_2 amines**	**X= NHOAc derivatized amines**
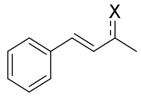	6.6	7.0	8.0	6.4	11.2
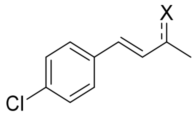	9	9.3	10.3	8.6	13.3
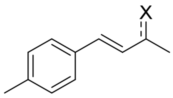	7.8	8.4	9.4	7.6	12.5
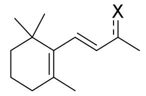	7.6	8.4	9.0	7.4	11.7

[a] Injector temperature: 250 °C; Injection volume: 1 µL; Flow rate: 0.7 mL/min; Temperature program (Standard Method): 100 °C, hold time 0.5 min, 10 °C/min to 300 °C, hold time 0 min; EI mode, energy 70 eV, MS Source: 230 °C, MS Quadrupole: 150 °C.

HPLC:

Chiral HPLC analysis was performed on a Shimadzu HPLC system using *n*-heptane/*i*PrOH as the eluents and the Daicel columns (indicated below) as the chiral stationary phase. The enantiomeric excess of the remaining alcohols, as well as produced amines, were determined by HPLC equipped with chiral columns (OD-H, AS-H, AD-H, and OJ) with different methods, which are mentioned in Tables S23-S26.

Table S23. Retention times of allylic alcohol (**1a**) and its corresponding ketone (**1b**) and amine (**1c**) measured with Shimadzu HPLC equipped with chiral columns [a]

	Retention time [min]		
	**X= OH** [a] **alcohol**	**X= =O** [a] **ketone**	**X= NHOAc derivatized amines**
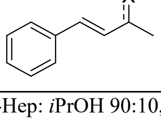	8.1 (*R*), 11.7 (*S*) [25]	7.4	10.4 (*R*), 15.1 (*S*) [a]<br>11.5 (*R*), 14.3 (*S*) [26] [b]

[a] Method: OD-H, *n*-Hep: *i*PrOH 90:10, Flow: 1.0 mL/min, 230 nm, Temperature: 25 ºC, 254 nm for derivatized amine.
[b] Method: AD-H, *n*-Hep: *i*PrOH 96:4, Flow: 0.7 mL/min, 254 nm, Temperature: 25 ºC.

Table S24. Retention times of allylic alcohol (**3a**) and its corresponding ketone (**3b**) and amine (**3c**) measured with Shimadzu HPLC equipped with OD-H column [a]

	**Retention time [min]** [a]		
	**X= OH alcohol**	**X= =O ketone**	**X= NHOAc derivatized amines**
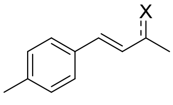	7.3 (*R*), 8.1 (*S*) [25]	6.1	7.8 (*R*), 8.7 (*S*) [a]<br>25.5 (*R*), 28.5 (*S*) [26] [b]

[a] Method: OD-H, *n*-Hep: *i*PrOH 90:10, Flow: 1.0 mL/min, 230 nm, Temperature: 25 ºC, 254 nm for derivatized amine.
[b] Method: AD-H, *n*-Hep: *i*PrOH 98:2, Flow: 0.7 mL/min, 254 nm, Temperature: 25 ºC.

Table S25. Retention times of allylic alcohol (**2a**) and its corresponding ketone (**2b**) and amine (**2c**) measured with Shimadzu HPLC equipped with chiral columns [a]

	**Retention time [min]**		
	**X= OH** [a] **alcohol**	**X= =O** [a] **ketone**	**X= NHOAc** [b] **derivatized amines**
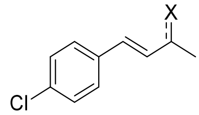	32.3 (*S*), 34.3 (*R*) [27]	40.6	65.5 (*R*), 72.4 (*S*) [b]<br>13.1 (*R*), 17.1 (*S*) [26] [c]<br>20.6 (*R*), 29.1 (*S*) [d]

[a] Method: AS-H, *n*-Hep: *i*PrOH 97:3, Flow: 0.5 mL/min, 230 nm, Temperature: 25 ºC.
[b] Method: AS-H, *n*-Hep: *i*PrOH 92:8, Flow: 0.7 mL/min, 230 nm, Temperature: 25 ºC.
[c] Method: AD-H, *n*-Hep: *i*PrOH 96:4, Flow: 0.7 mL/min, 254 nm, Temperature: 25 ºC.
[d] Method: OJ, *n*-Hep: *i*PrOH 90:10, Flow: 0.7 mL/min, 254 nm, Temperature: 25 ºC.

Table S26. Retention times of allylic alcohol (**4a**) and its corresponding ketone (**4b**) and amine (**4c**) measured with Shimadzu HPLC equipped with AS-H column [a]

	**Retention time [min]** [a]		
	**X= OH alcohol**	**X= =O ketone**	**X= NHOAc** [b] **derivatized amines**
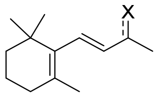	9.4 (*S*), 10.4 (*R*) [28]	14.7	18.5 (*R*), 21.9 (*S*) [b]

[a] Method: AS-H, *n*-Hep: *i*PrOH 97:3, Flow: 0.5 mL/min, 230 nm, Temperature: 25 ºC.
[b] Method: OJ, *n*-Hep: *i*PrOH 99:1, Flow: 0.7 mL/min, 230 nm, Temperature: 25 ºC.

4. Supplementary

4.1. NMRs

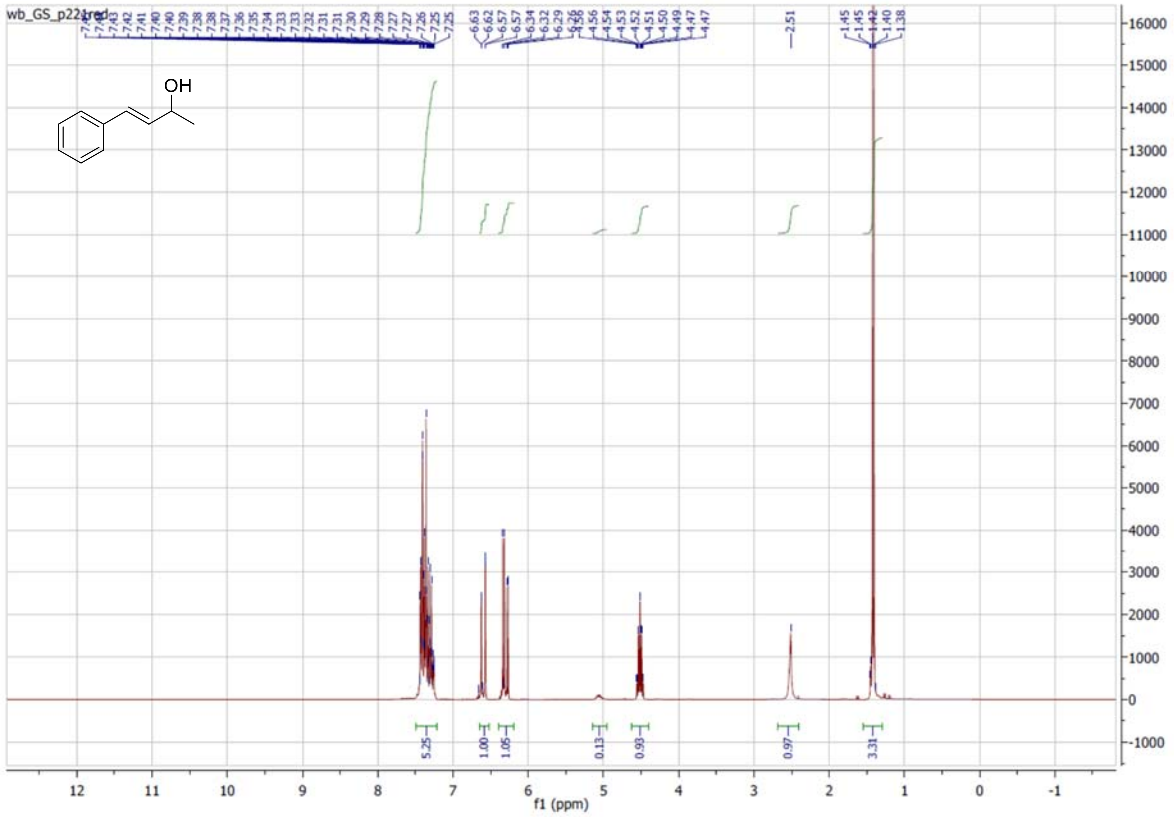

Figure S8. ^{1}H NMR of 4-phenylbut-3-en-2-ol (**1a**) in $CDCl_3$

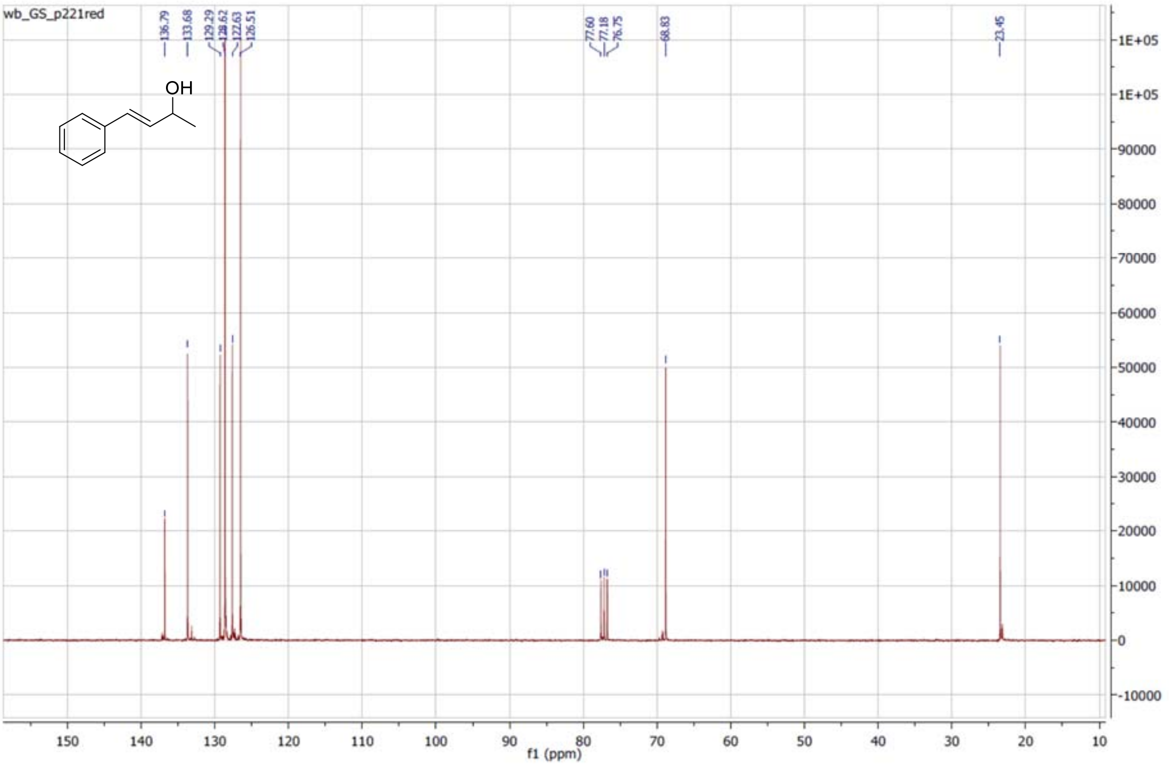

Figure S9. ^{13}C NMR of 4-phenylbut-3-en-2-ol (**1a**) in $CDCl_3$

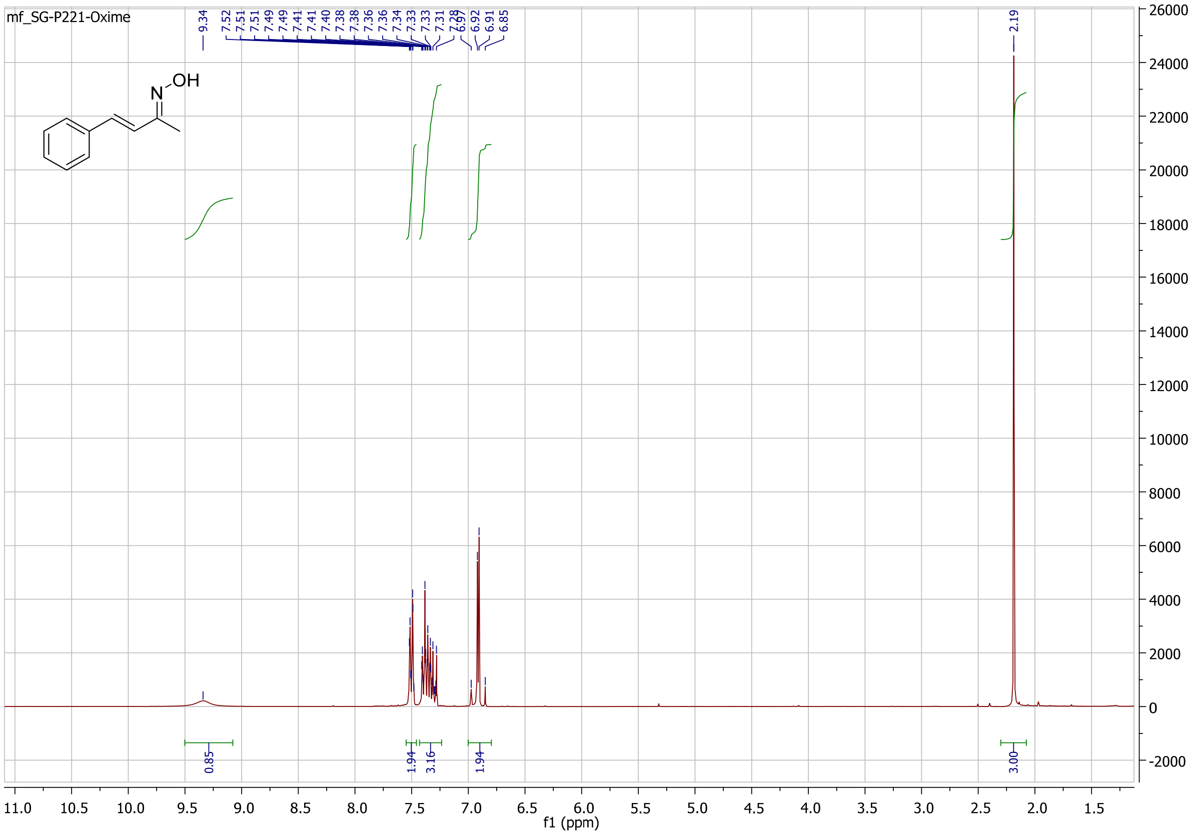

Figure S10. ^{1}H NMR of (*E*)-4-phenylbut-3-en-2-one oxime in $CDCl_3$

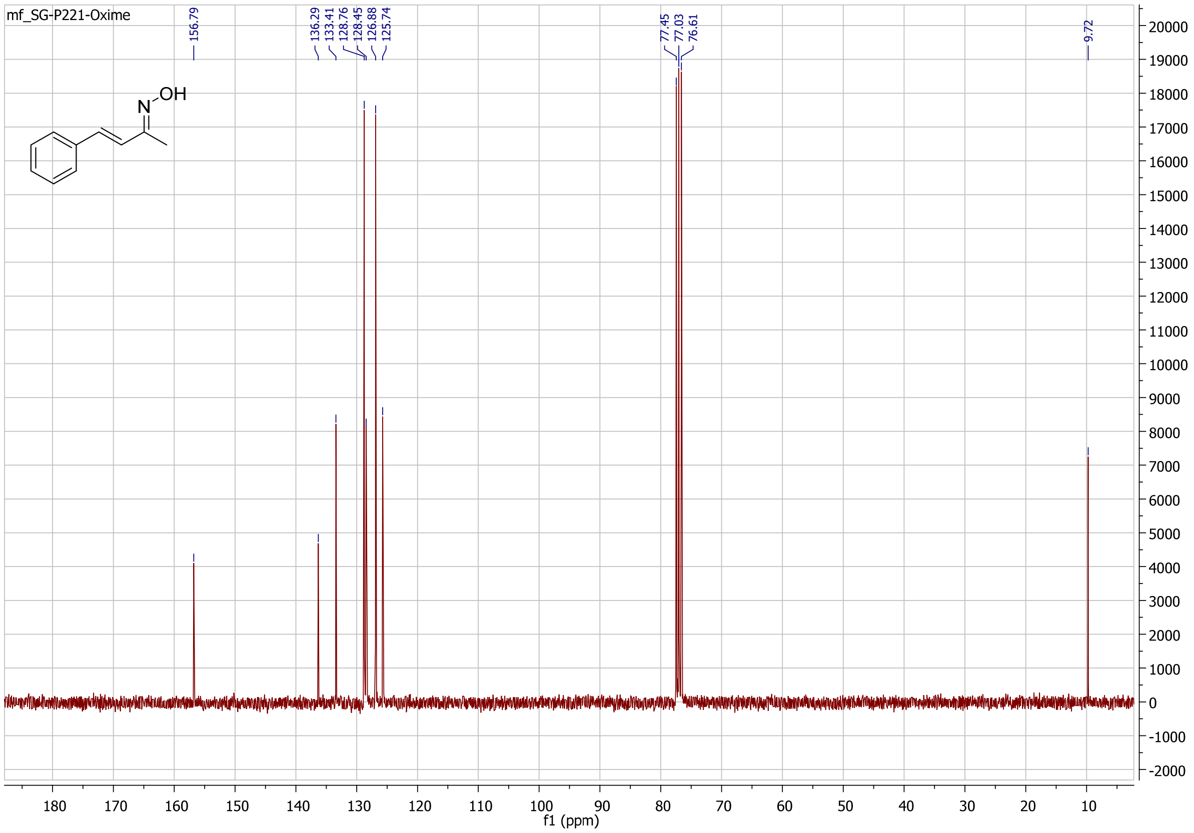

Figure S11. ^{13}C NMR of (*E*)-4-phenylbut-3-en-2-one oxime in $CDCl_3$

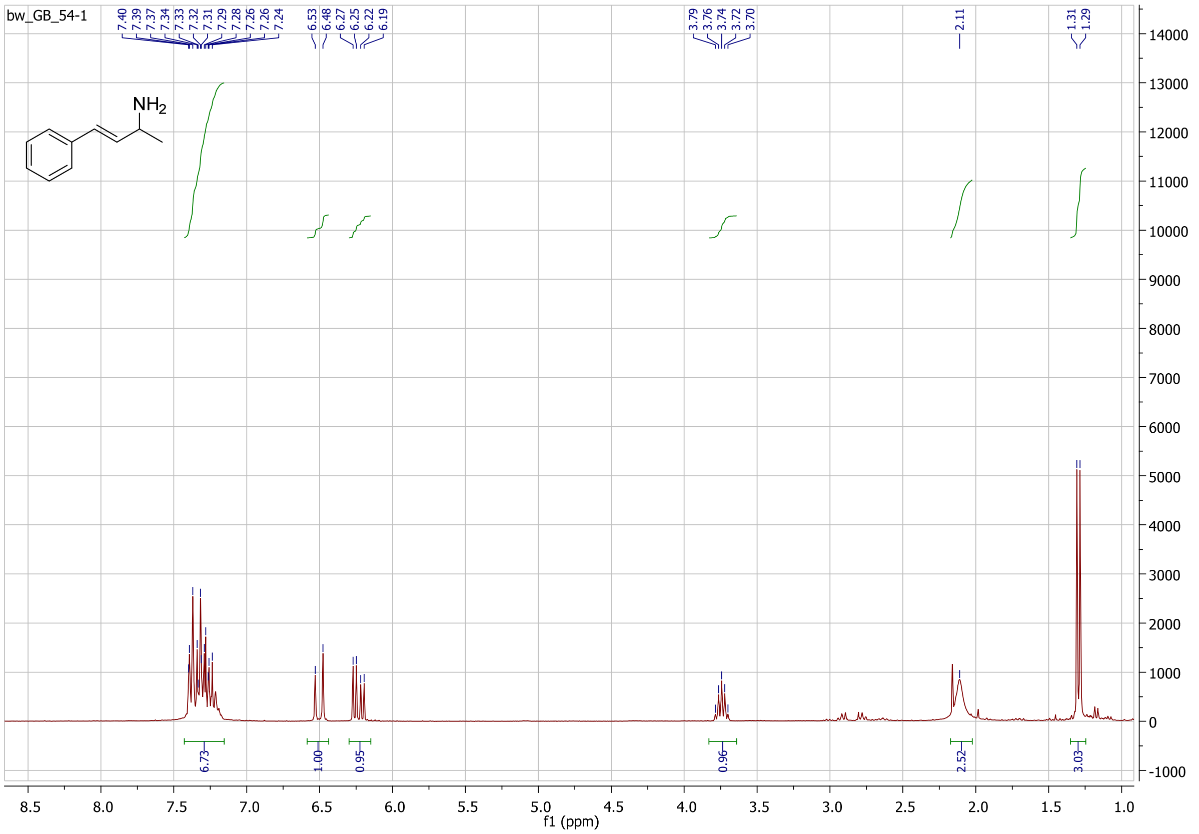

Figure S12. ^{1}H NMR of (*E*)-4-phenylbut-3-en-2-amine (**1c**) in $CDCl_3$

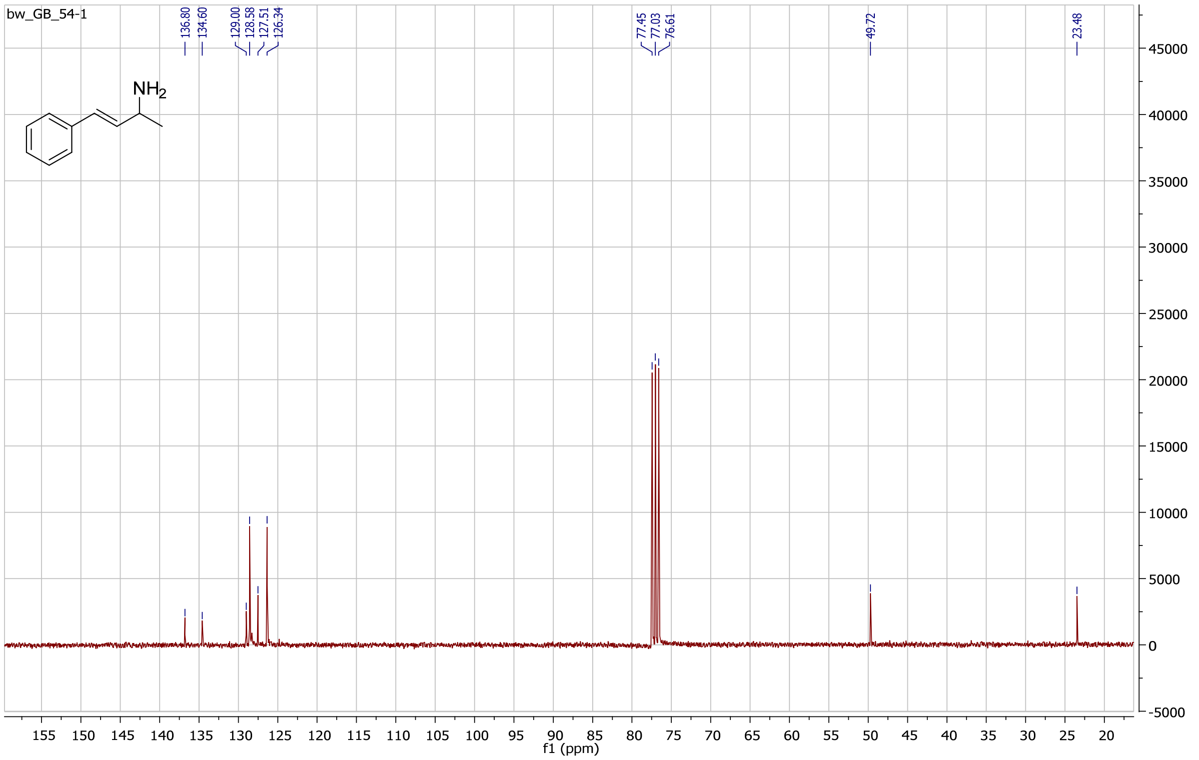

Figure S13. ^{13}C NMR of (*E*)-4-phenylbut-3-en-2-amine (**1c**) in $CDCl_3$

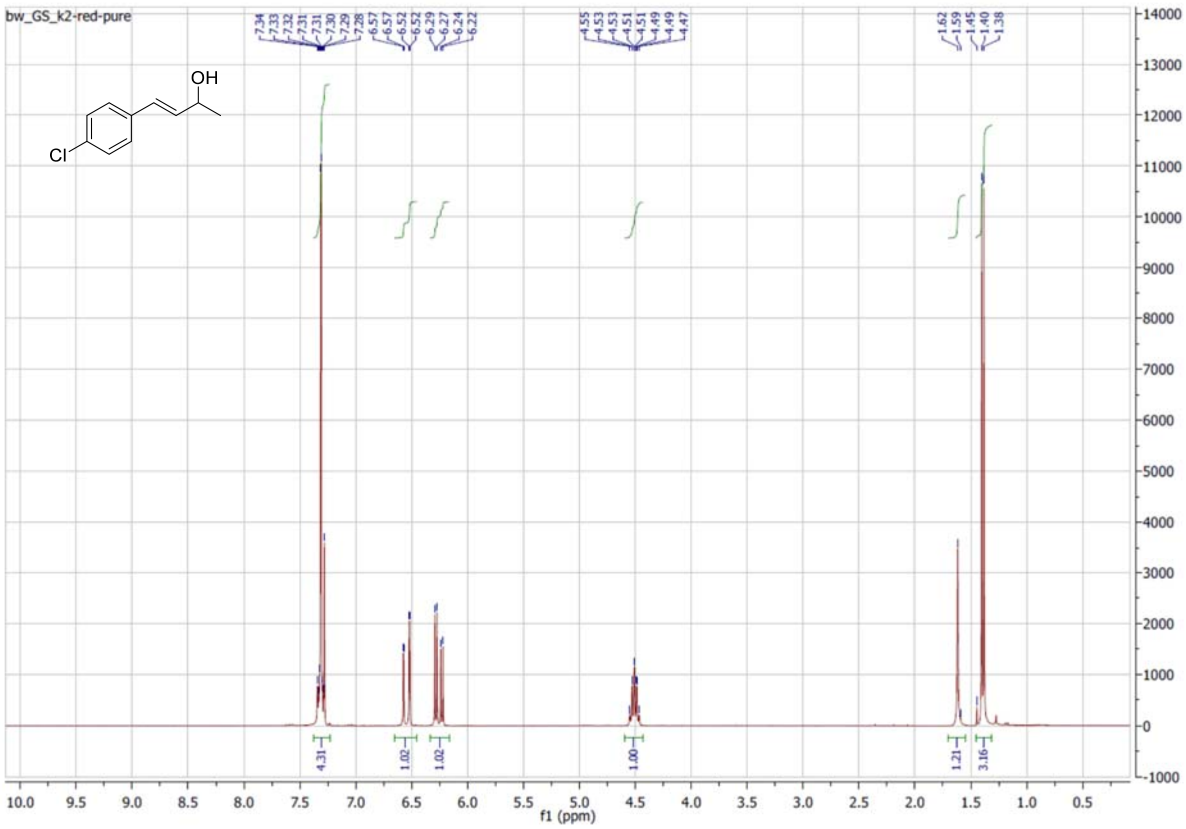

Figure S14. ^{1}H NMR of (*E*)-4-(4-chlorophenyl)but-3-en-2-ol (**2a**) in $CDCl_3$

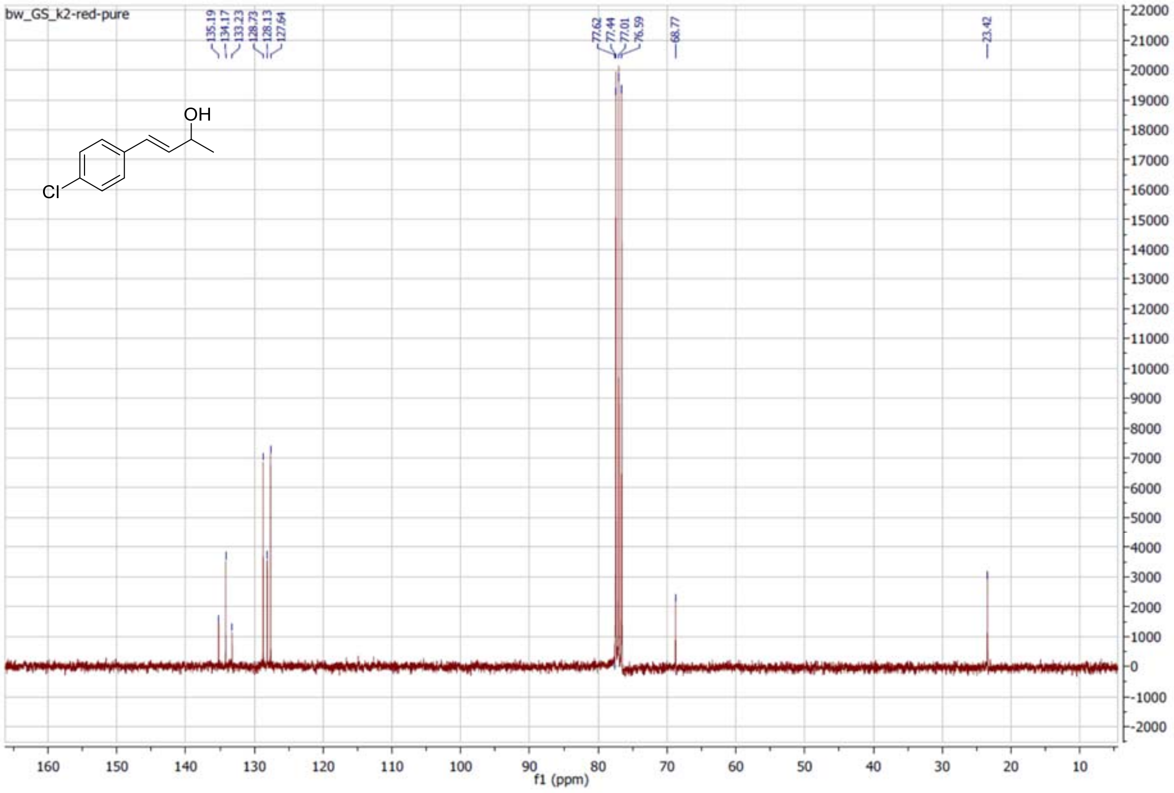

Figure S15. ^{13}C NMR of (*E*)-4-(4-chlorophenyl)but-3-en-2-ol (**2a**) in $CDCl_3$

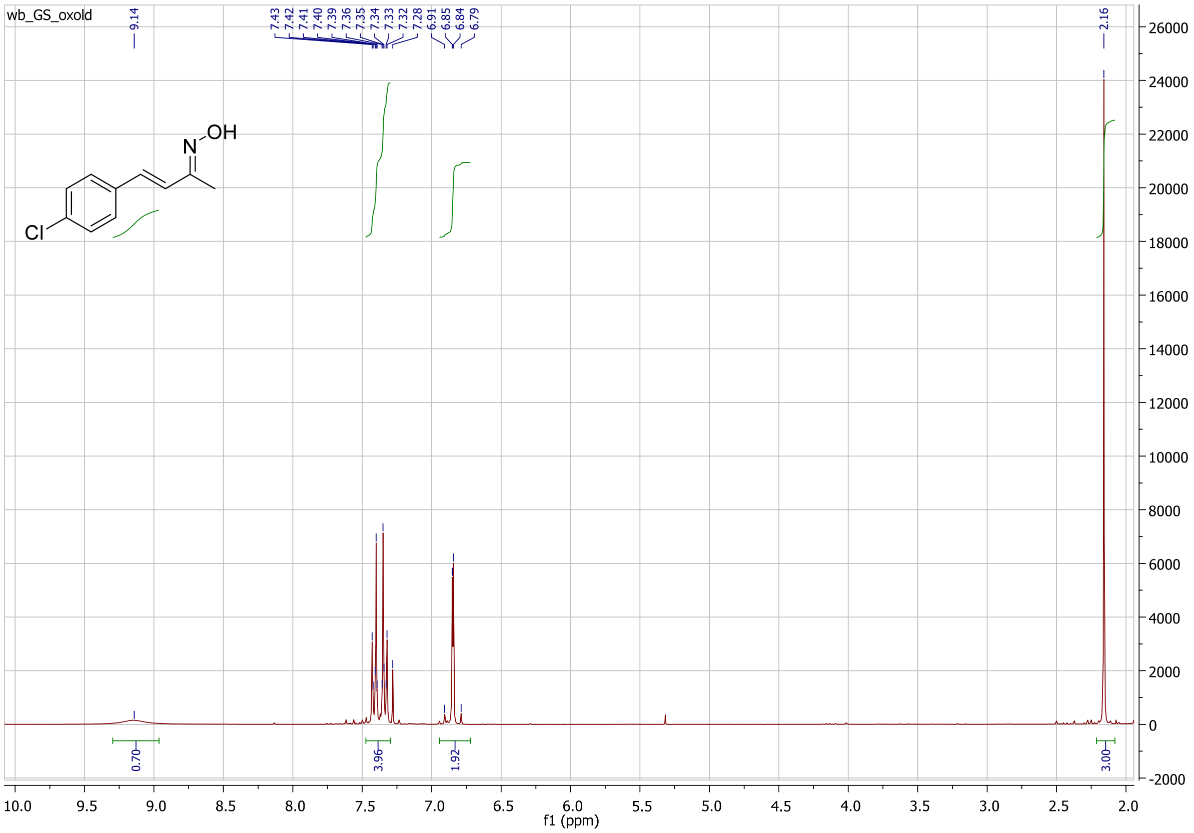

Figure S16. ^{1}H NMR of (*E*)-4-(4-chlorophenyl)but-3-en-2-one oxime in $CDCl_3$

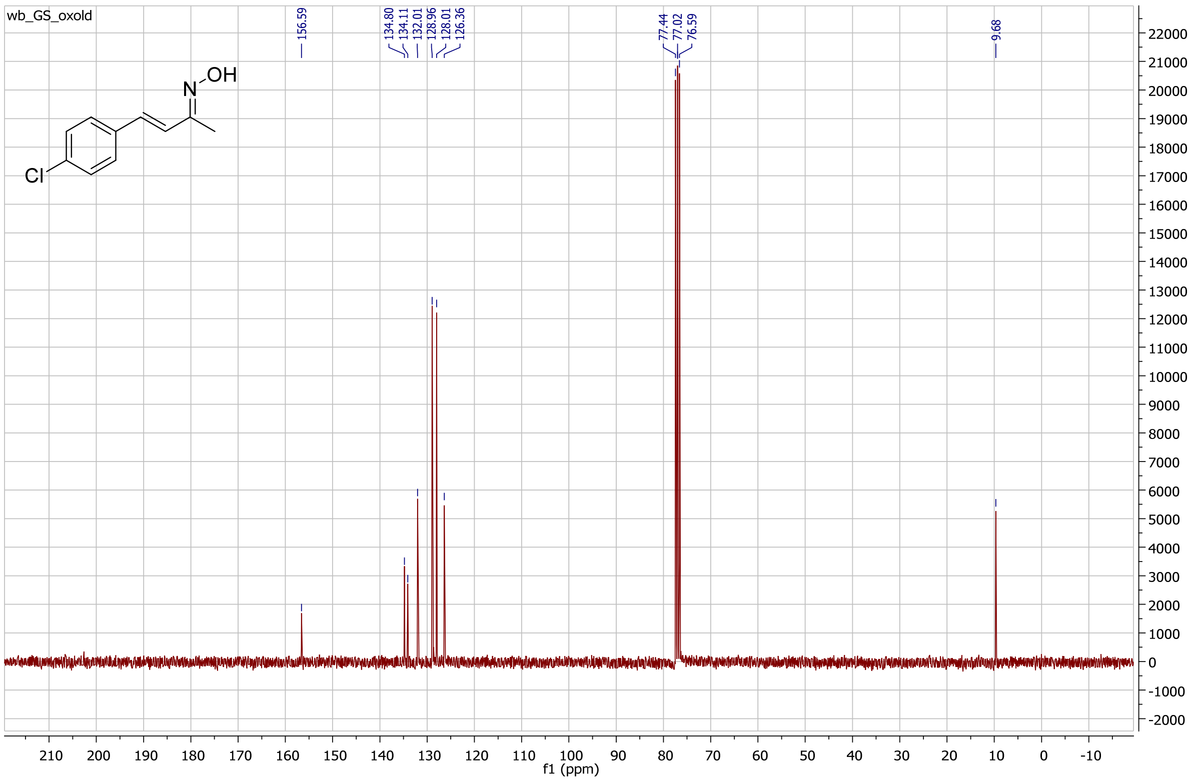

Figure S17. ^{13}C NMR of (*E*)-4-(4-chlorophenyl)but-3-en-2-one oxime in $CDCl_3$

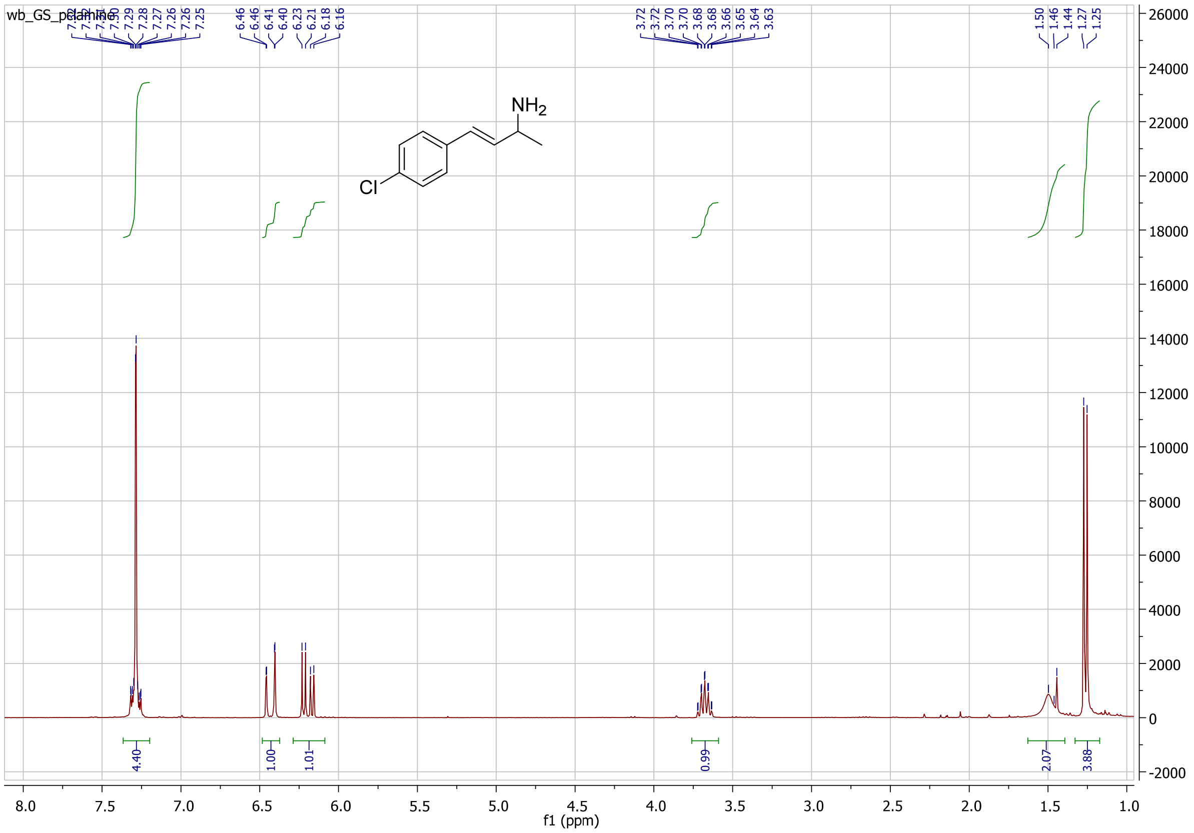

Figure S18. ^{1}H NMR of (*E*)-4-(4-chlorophenyl)but-3-en-2-amine (**2c**) in $CDCl_3$

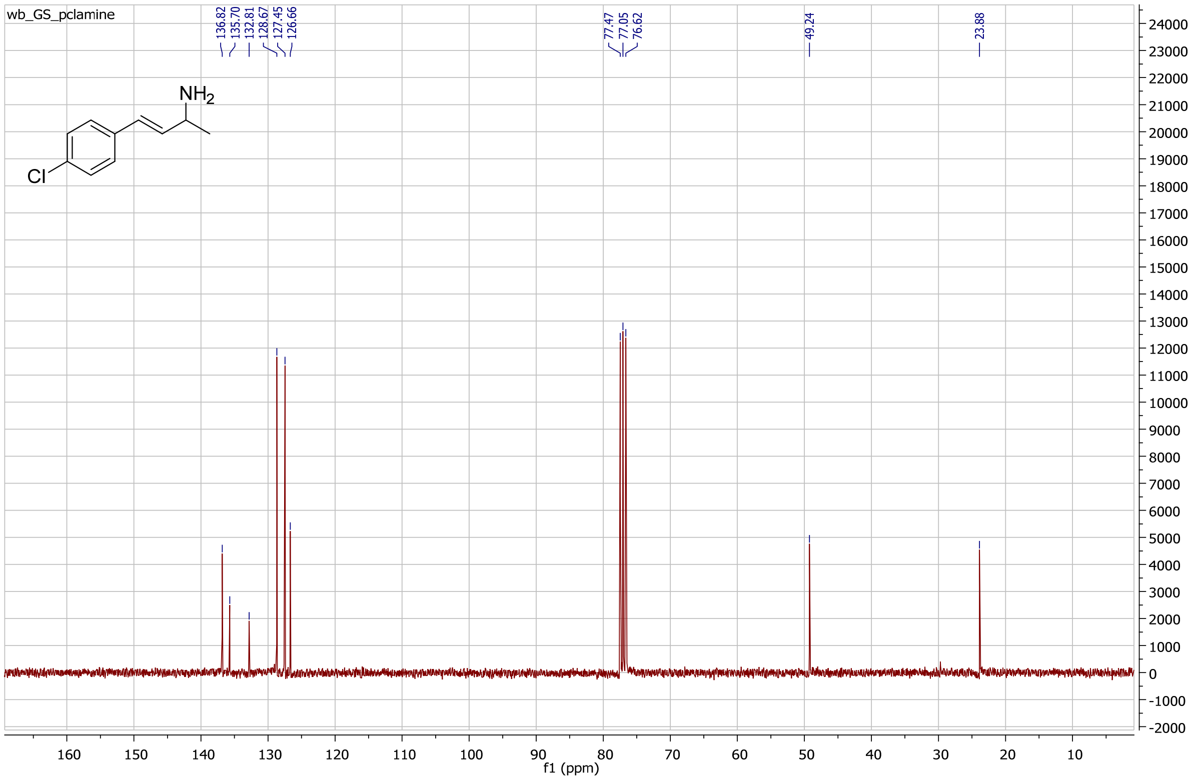

Figure S19. ^{13}C NMR of (*E*)-4-(4-chlorophenyl)but-3-en-2-amine (**2c**) in $CDCl_3$

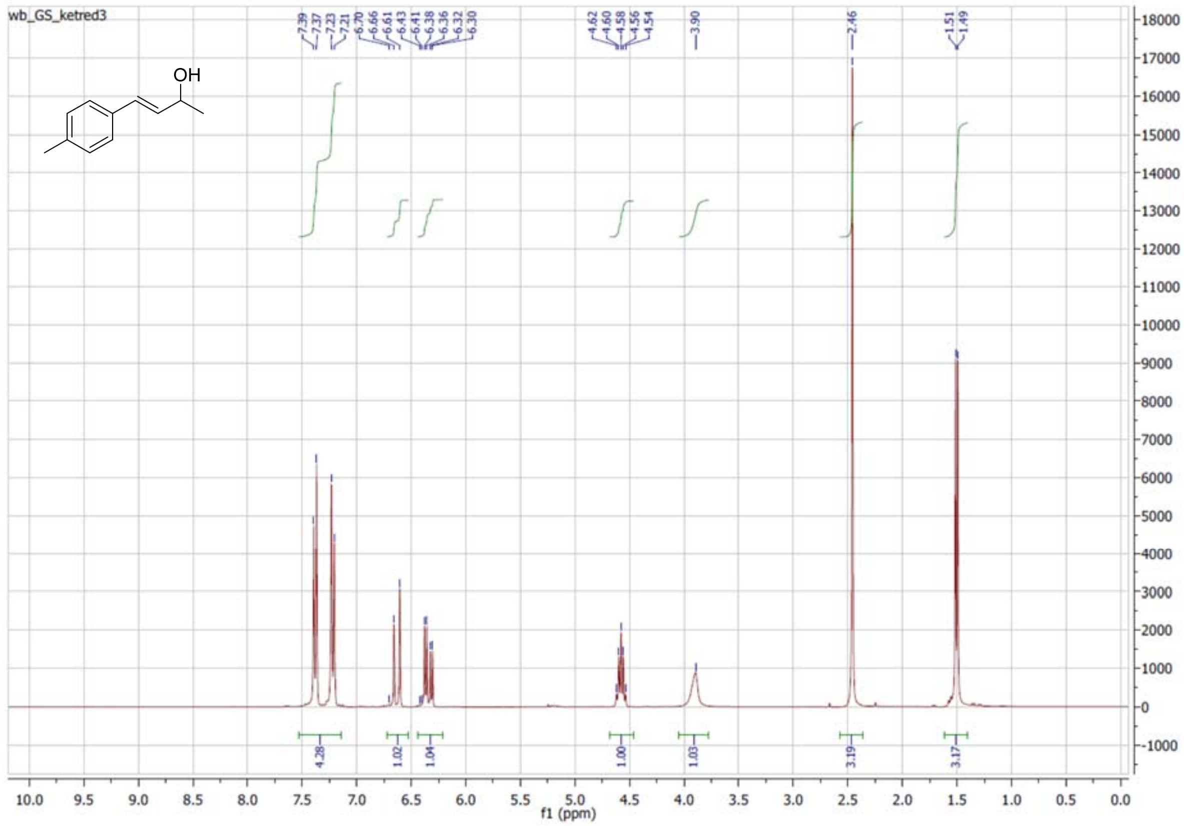

Figure S20. ^{1}H NMR of (*E*)-4-(*p*-tolyl)but-3-en-2-ol (**3a**) in $CDCl_3$

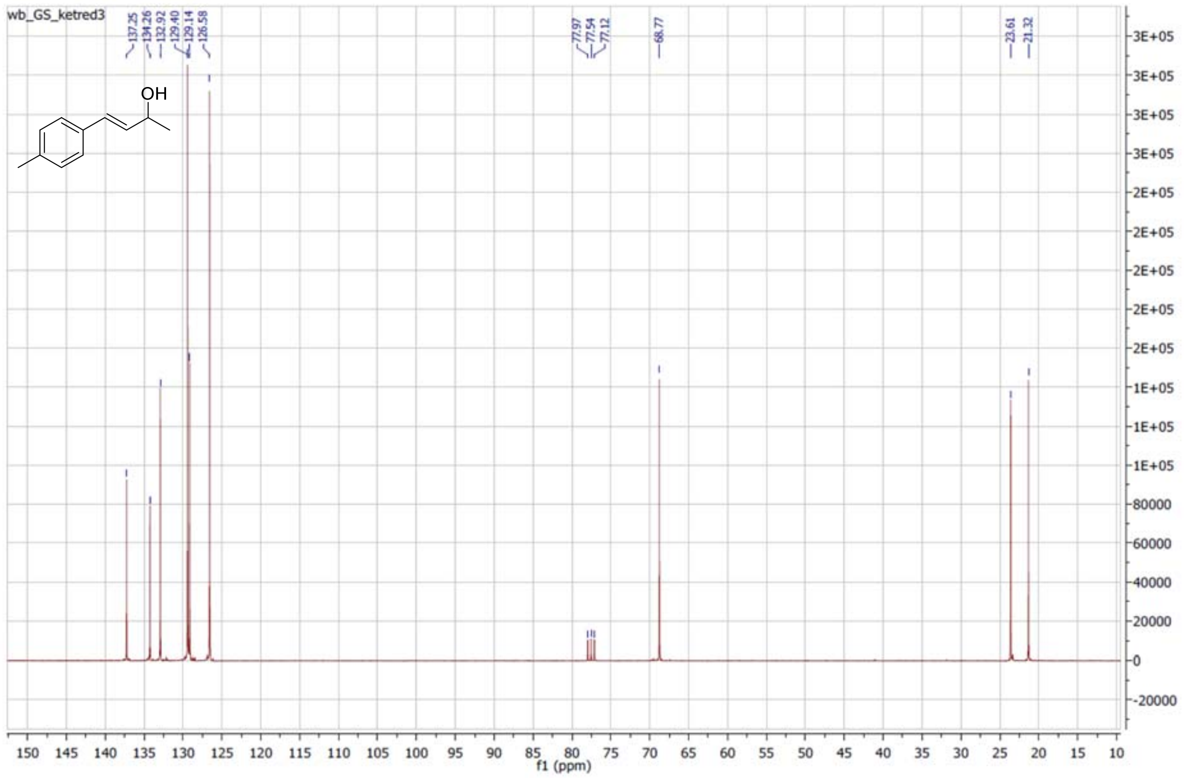

Figure S21. ^{13}C NMR of (*E*)-4-(*p*-tolyl)but-3-en-2-ol (**3a**) in $CDCl_3$

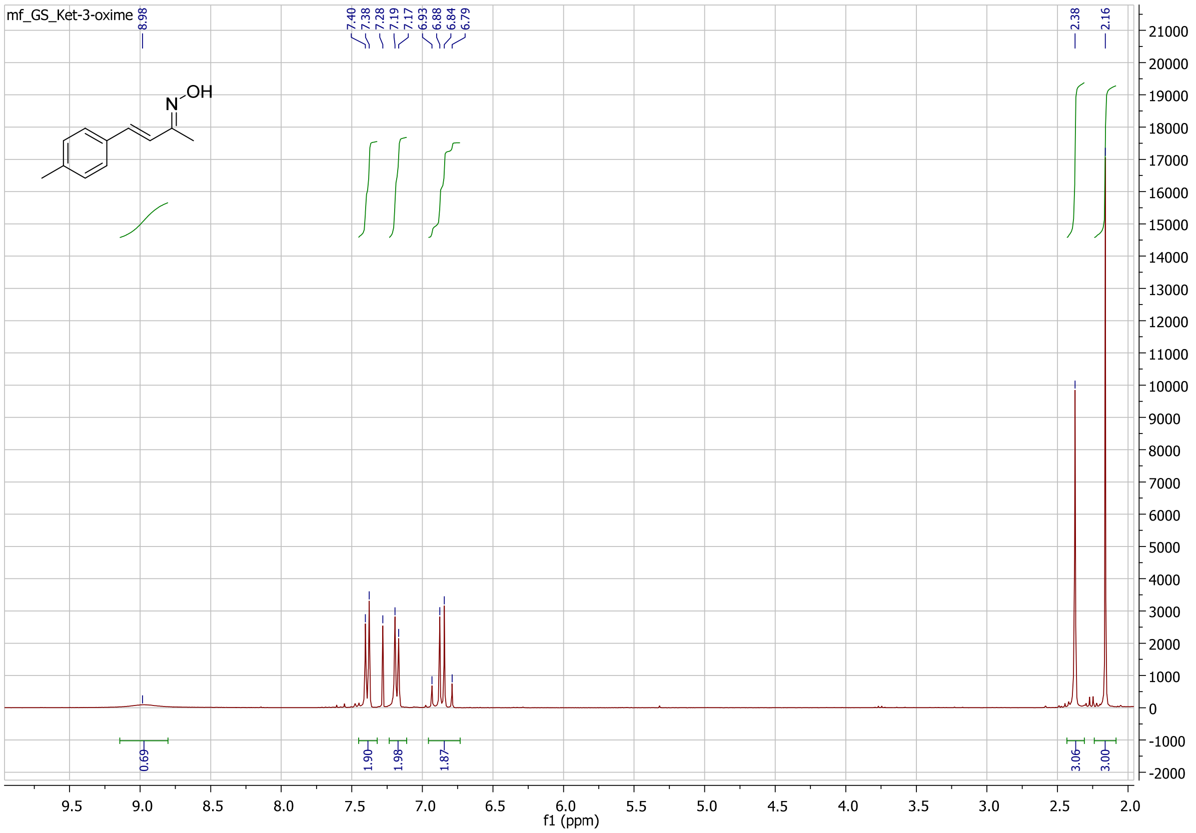

Figure S22. ^{1}H NMR of (*E*)-4-(*p*-tolyl)but-3-en-2-one oxime in $CDCl_3$

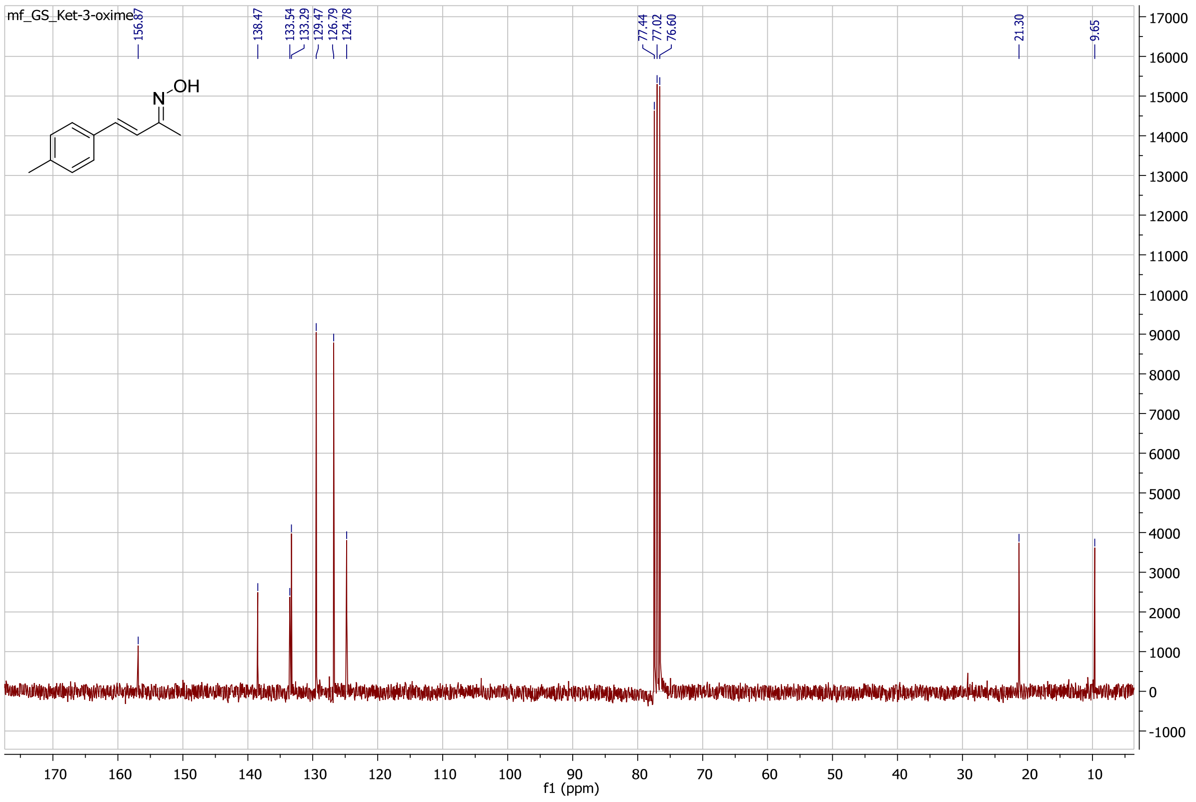

Figure S23. ^{13}C NMR of (*E*)-4-(*p*-tolyl)but-3-en-2-one oxime in $CDCl_3$

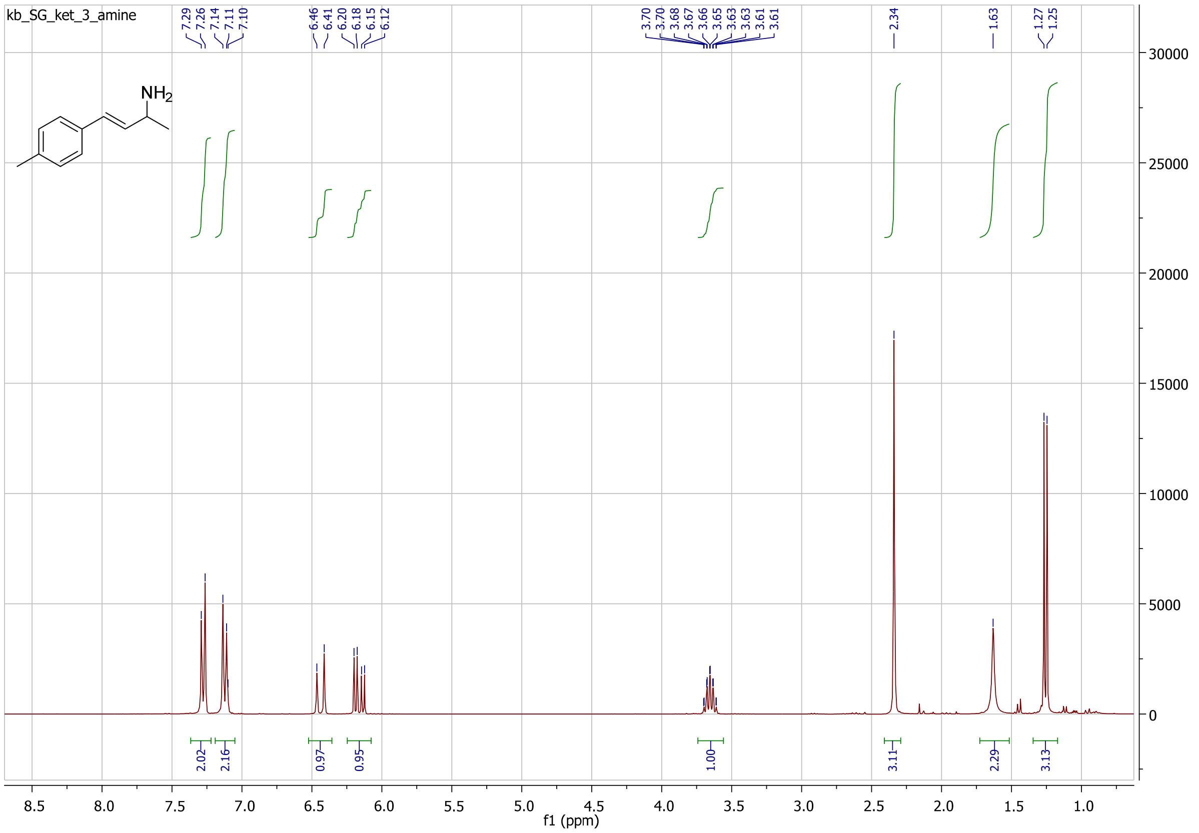

Figure S24. ^{1}H NMR of (*E*)-4-(*p*-tolyl)but-3-en-2-amine (**3c**) in $CDCl_3$

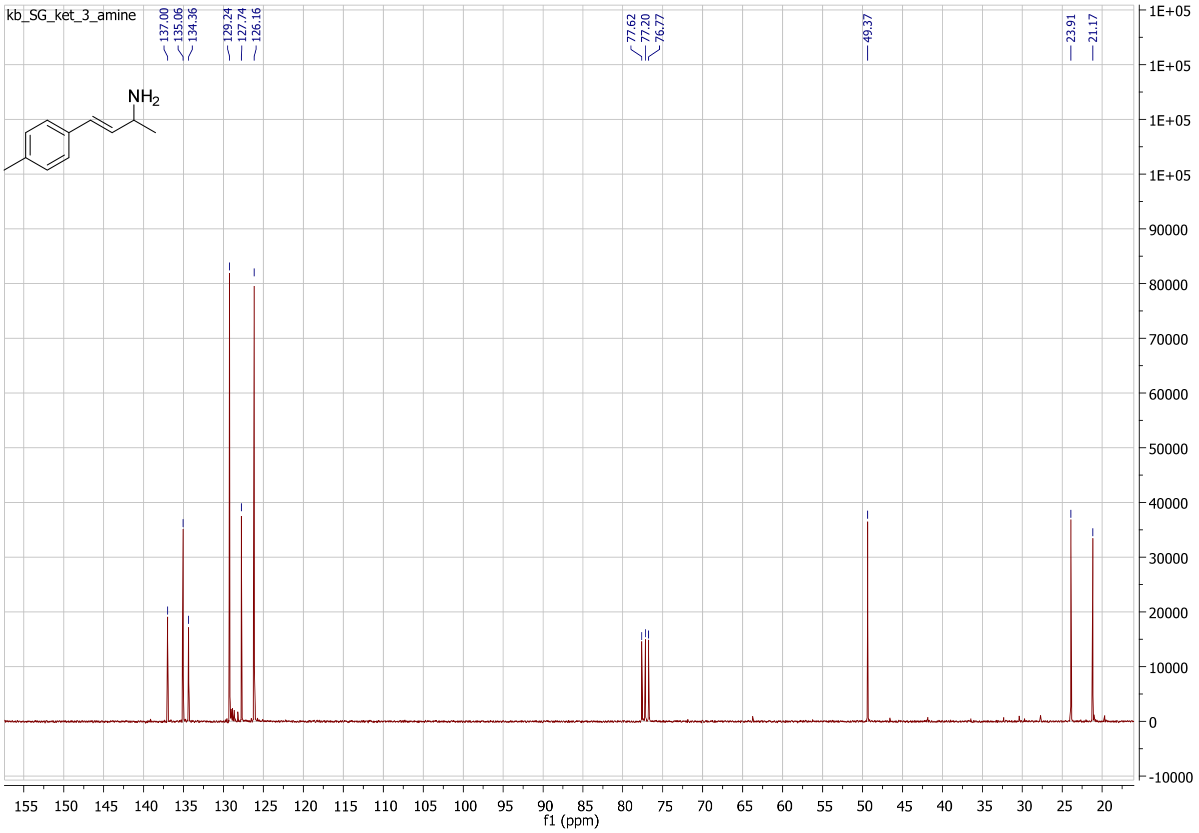

Figure S25. ^{13}C NMR of (*E*)-4-(*p*-tolyl)but-3-en-2-amine (**3c**) in $CDCl_3$

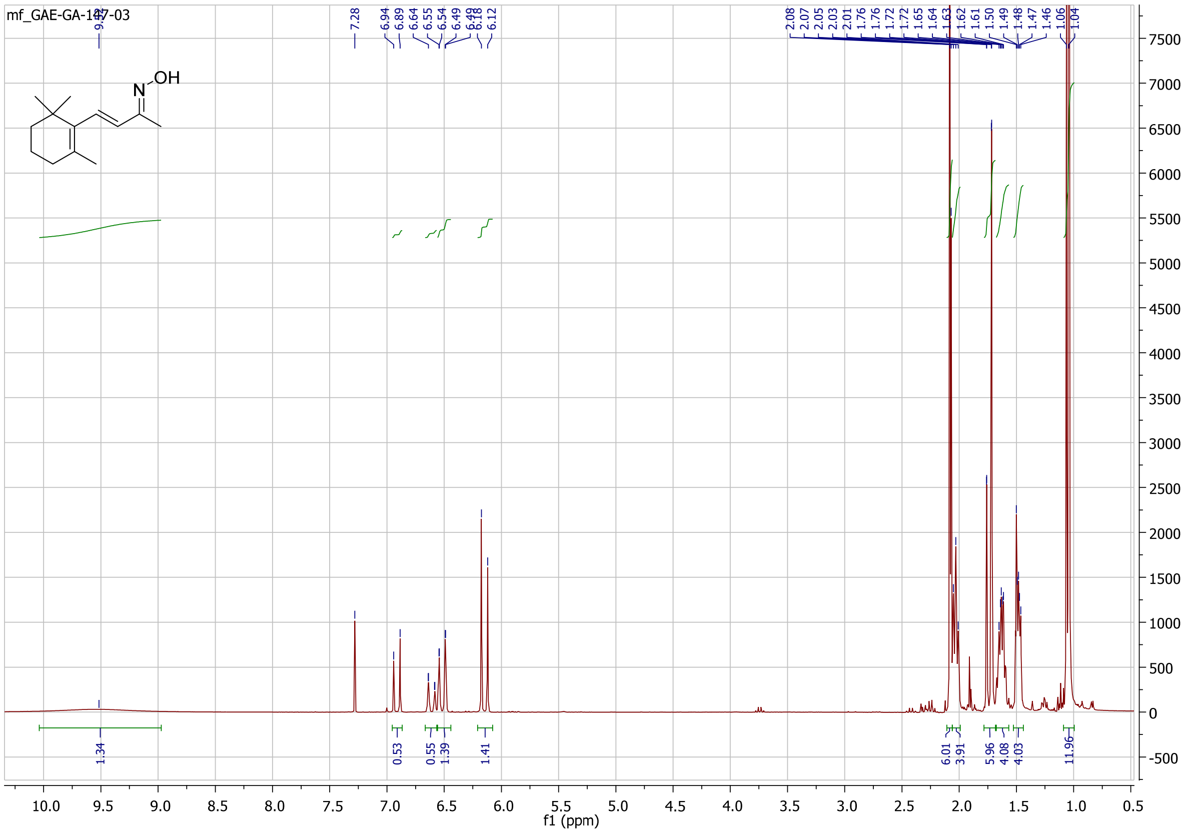

Figure S26. ^{1}H NMR of (*E*)-4-(2,6,6-trimethylcyclohex-1-en-1-yl)but-3-en-2-one oxime in $CDCl_3$

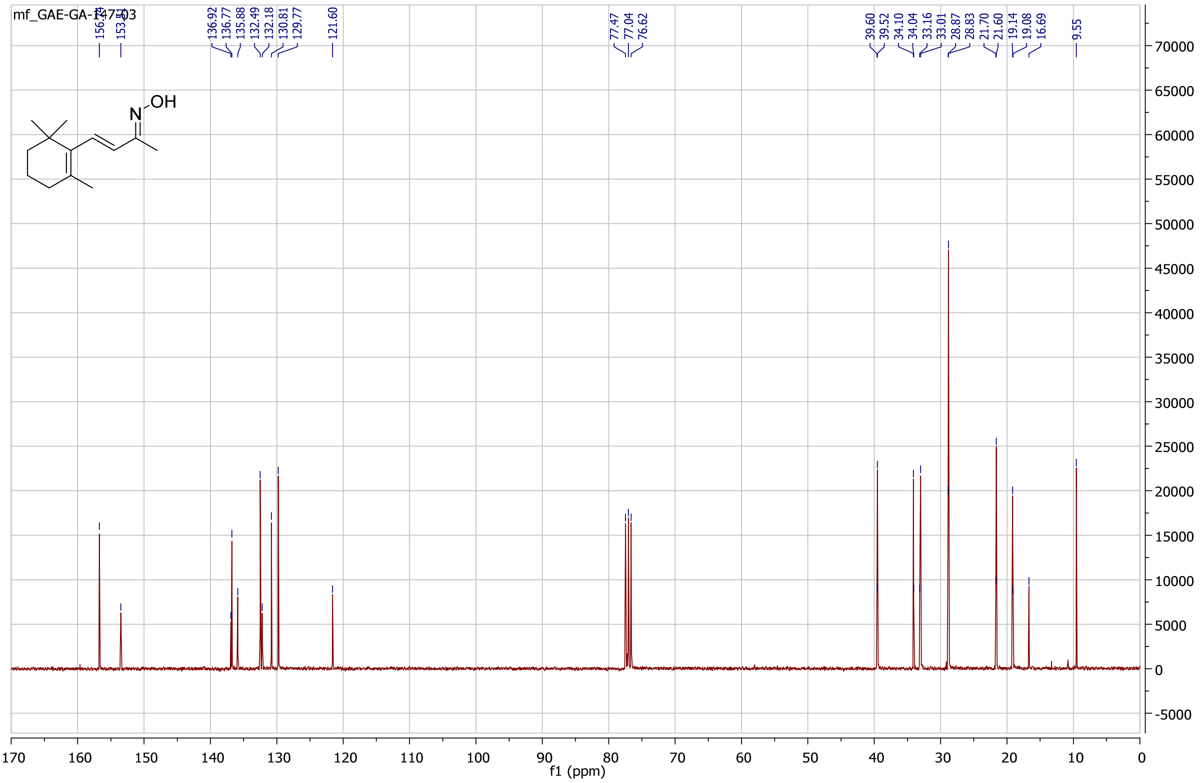

Figure S27. ^{13}C NMR of (*E*)-4-(2,6,6-trimethylcyclohex-1-en-1-yl)but-3-en-2-one oxime in $CDCl_3$

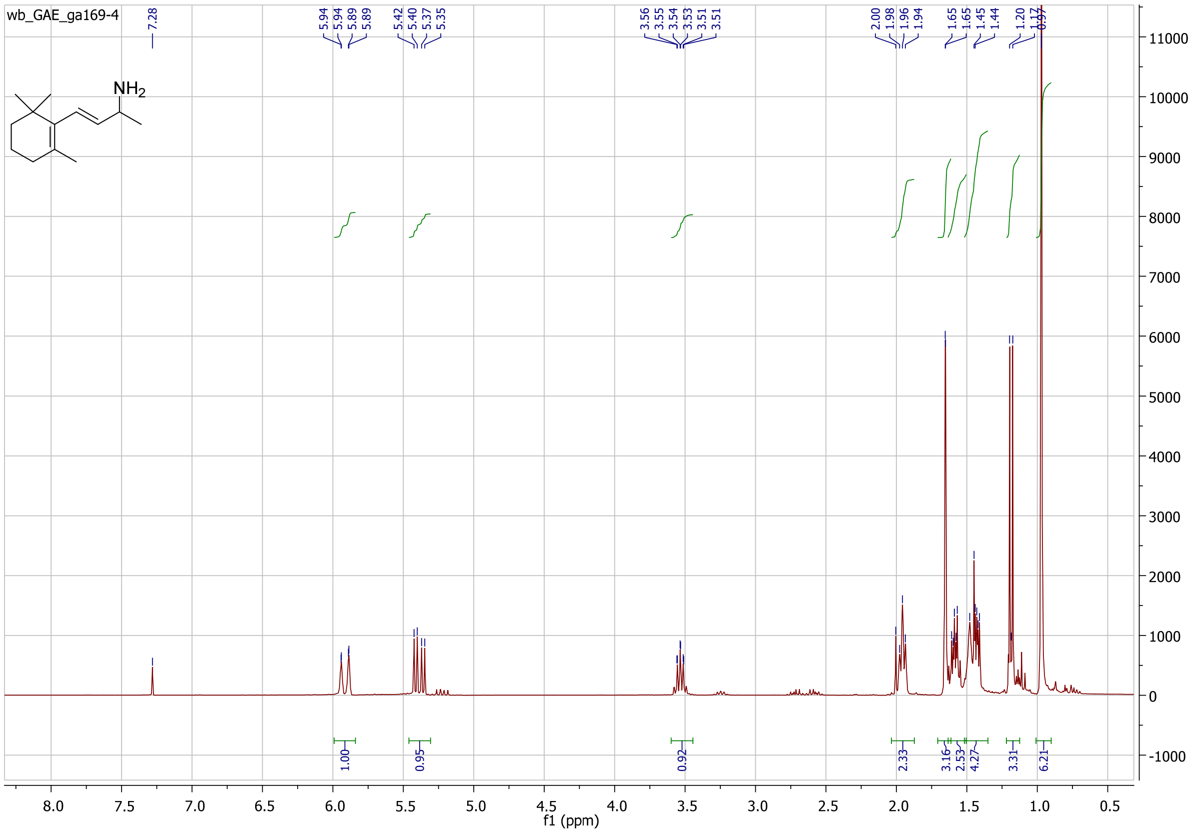

Figure S28. ^{1}H NMR of (*E*)-4-(2,6,6-trimethylcyclohex-1-en-1-yl)but-3-en-2-amine (**4c**) in $CDCl_3$

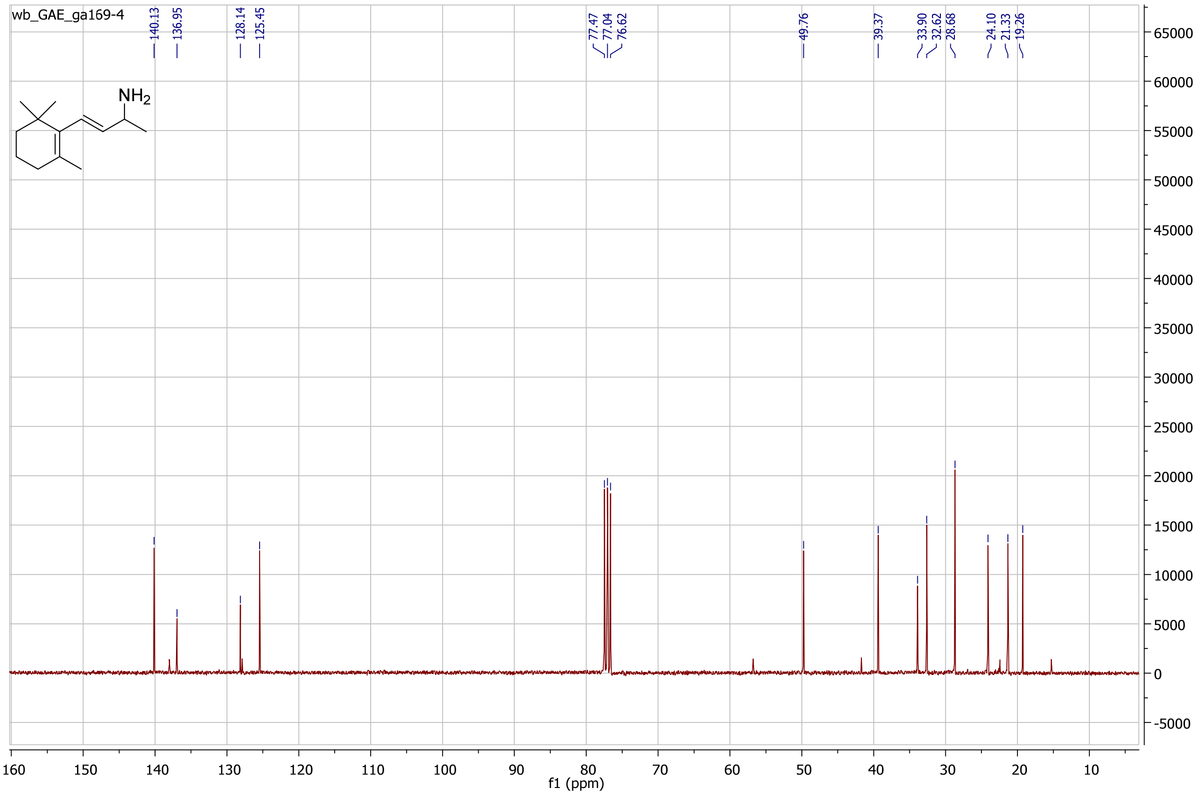

Figure S29. ^{13}C NMR of (*E*)-4-(2,6,6-trimethylcyclohex-1-en-1-yl)but-3-en-2-amine (**4c**) in $CDCl_3$

4.2. NMRs of purified amines from 0.2 mmol scale

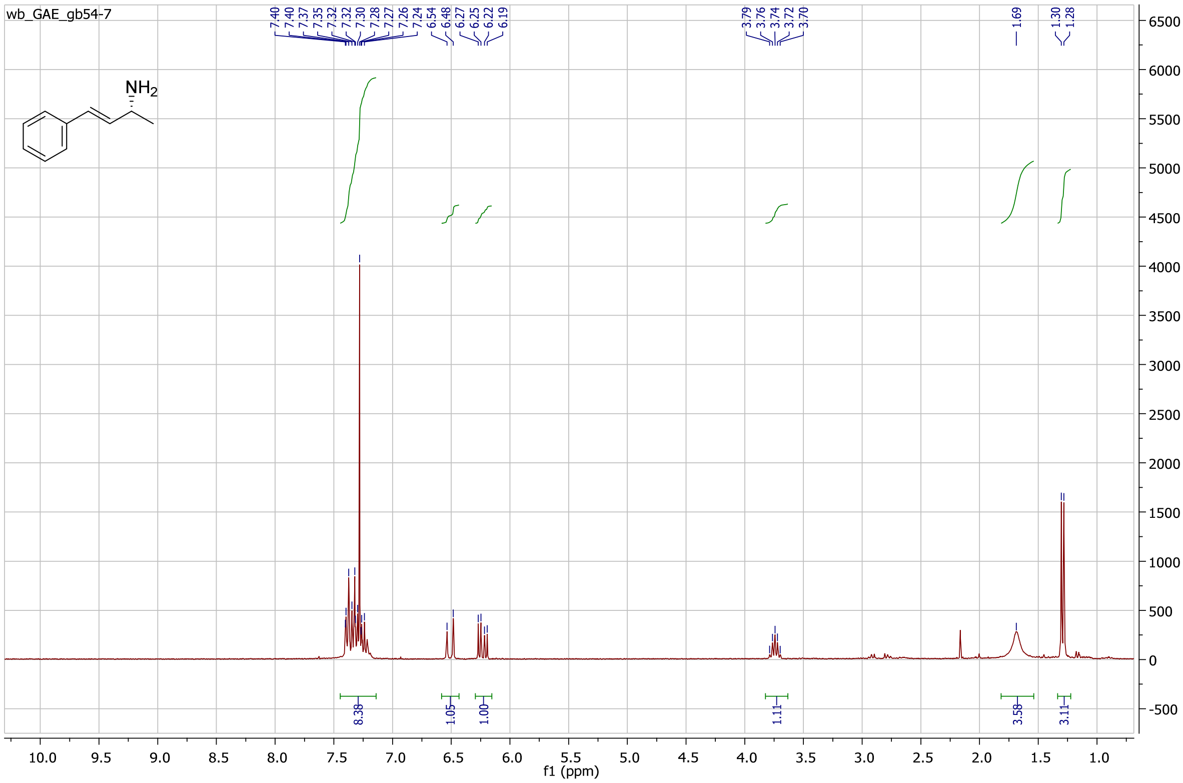

Figure S30. ^{1}H NMR of (*R*)-(*E*)-4-phenylbut-3-en-2-amine (**1c**) in $CDCl_3$

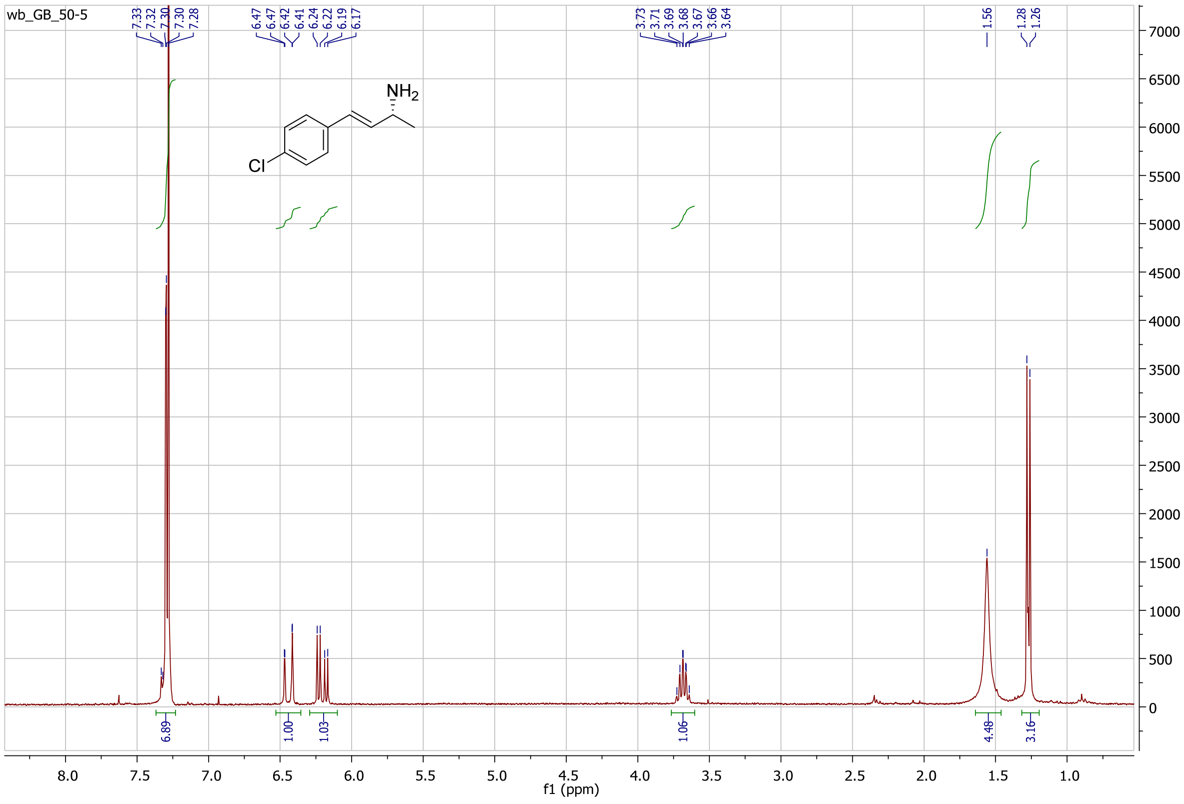

Figure S31. ^{1}H NMR of (*R*)-(*E*)-4-(4-chlorophenyl)but-3-en-2-amine (**2c**) in $CDCl_3$

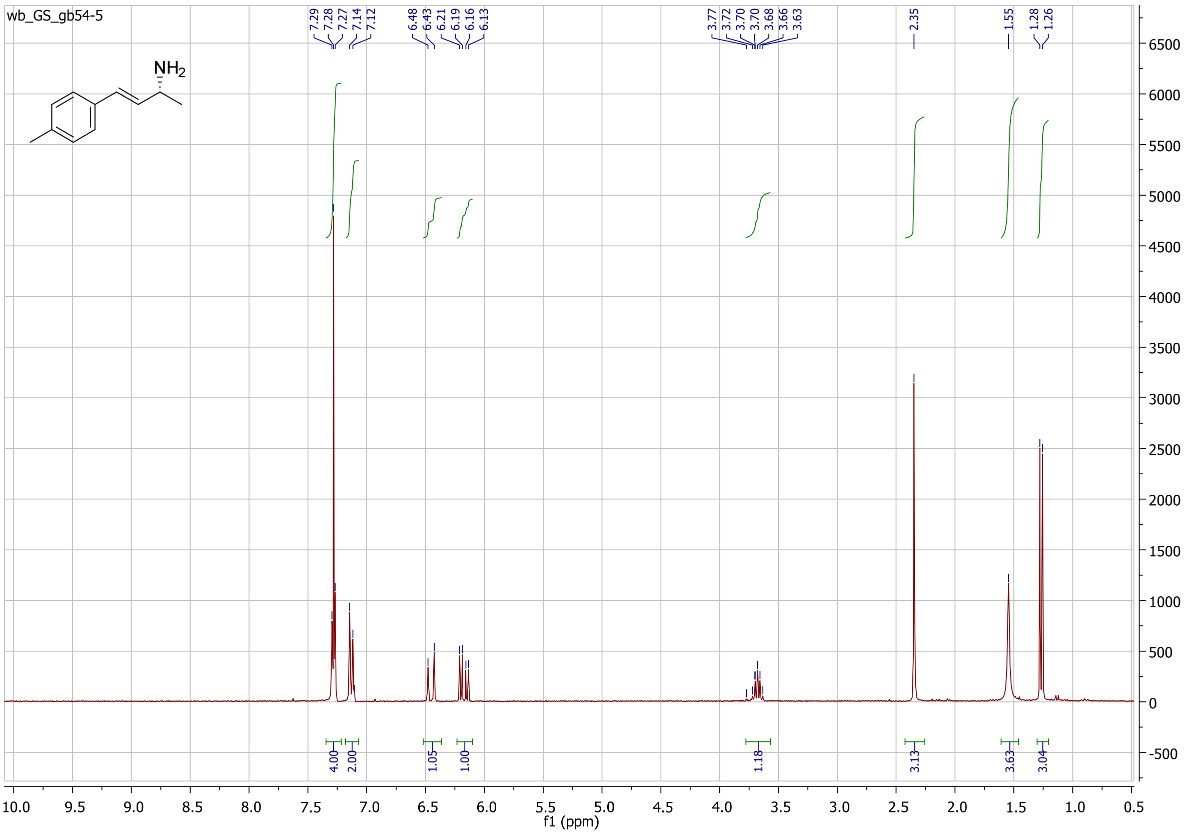

Figure S32. ^{1}H NMR of (*R*)-(*E*)-4-(*p*-tolyl)but-3-en-2-amine (**3c**) in $CDCl_3$

4.3. HPLC chromatograms

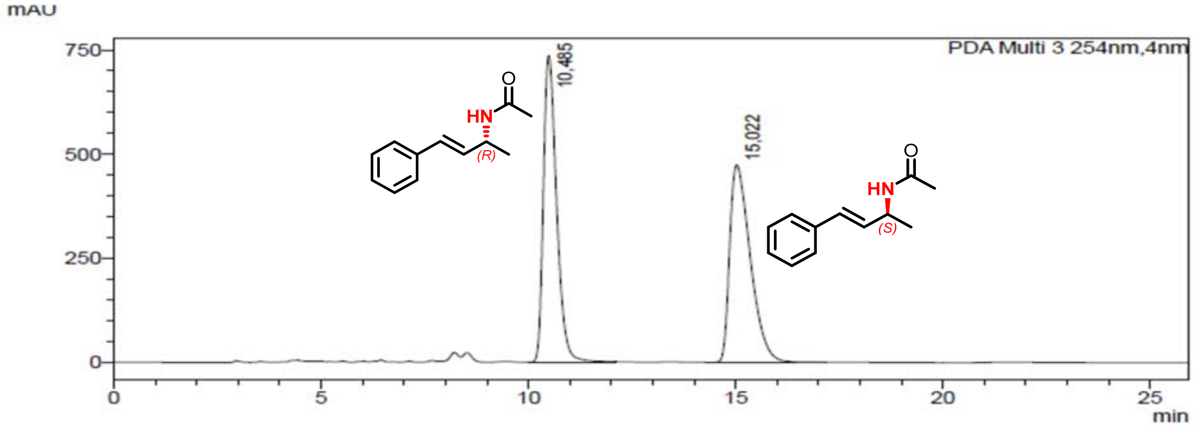

<Peak Table>

PDA Ch3 254nm

Peak#	Ret. Time	Name	Area%
1	10,485		49,948
2	15,022		50,052
Total			100,000

Figure S33. HPLC chromatogram of derivatized *rac*-(*E*)-4-phenylbut-3-en-2-amine using OD-H column (method: *n*-hept:*i*PrOH, 90:10, flow 1.0 mL/min, 25 ºC)

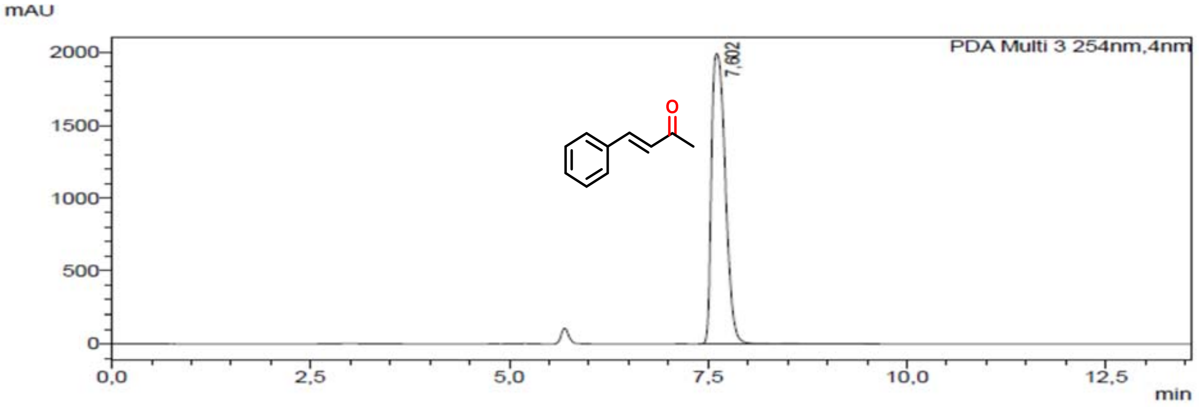

Figure S34. HPLC chromatogram of (*E*)-4-phenylbut-3-en-2-one using OD-H column (method: *n*-hept:*i*PrOH, 90:10, flow 1.0 mL/min, 25 ºC)

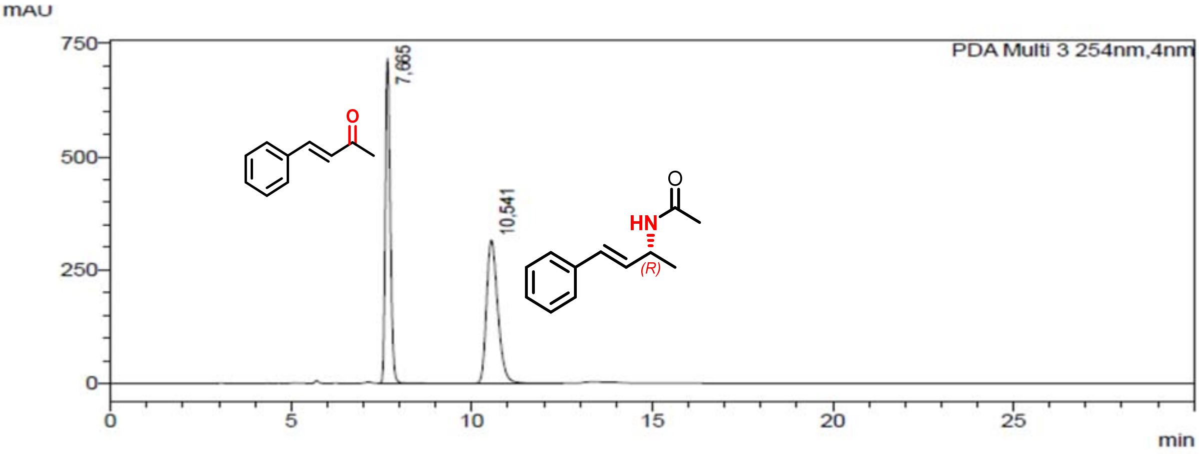

Figure S35. HPLC chromatogram of derivatized reaction mixture from ArRmut11 transaminase ((*R*)-selective transaminase) and isopropylamine as the amine donor using OD-H column (method: *n*-hept:*i*PrOH, 90:10, flow 1.0 mL/min, 25 ºC)

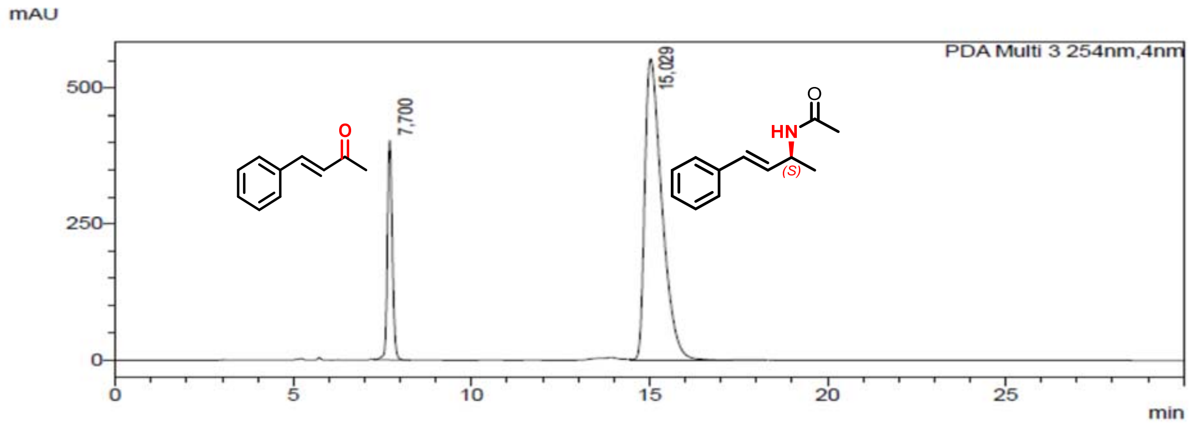

Figure S36. HPLC chromatogram of derivatized reaction mixture from pEG 205 transaminase ((*S*)-selective transaminase) and isopropylamine as the amine donor using OD-H column (method: *n*-hept:*i*PrOH, 90:10, flow 1.0 mL/min, 25 ºC)

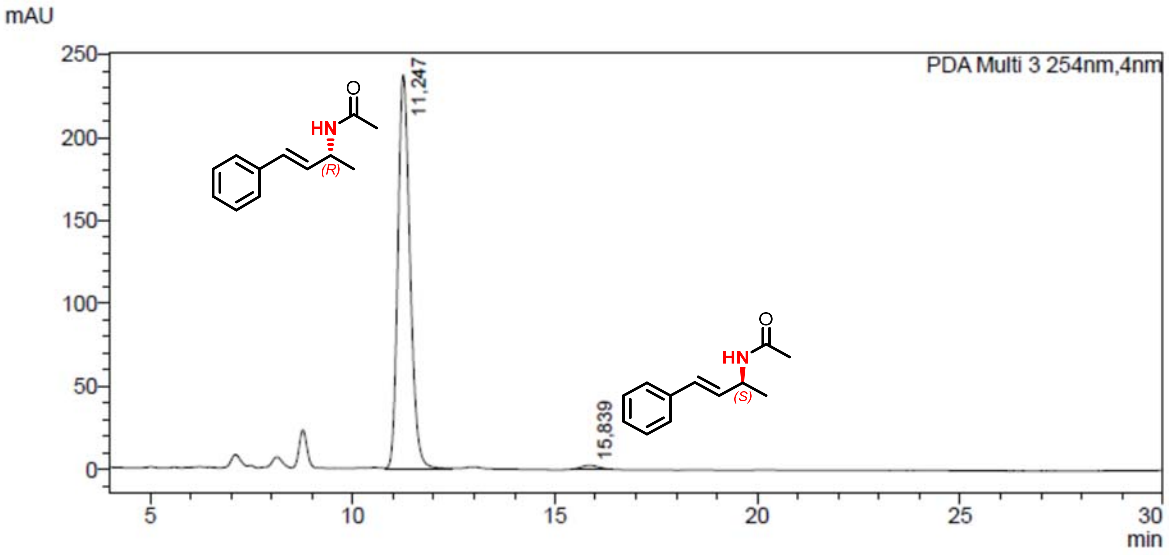

<Peak Table>

PDA Ch3 254nm

Peak#	Ret. Time	Area	Height	Area%
1	11,247	4980444	237300	98,698
2	15,839	65689	2286	1,302
Total		5046134	239586	100,000

Figure S37. HPLC chromatogram of derivatized (*R*)-**1c** from 0.2 mmol scale cascade with *rac*-**1a**, PQQ-DH, ArRmut11 transaminase and isopropylamine as the amine donor using OD-H column (method: *n*-hept:*i*PrOH, 90:10, flow 1.0 mL/min, 25 ºC)

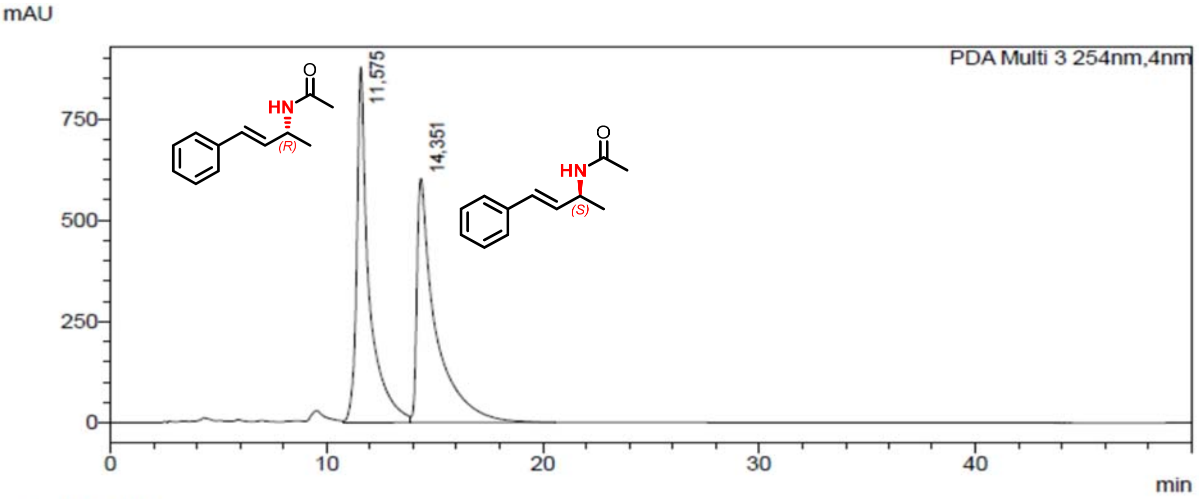

<Peak Table>

PDA Ch3 254nm

Peak#	Ret. Time	Area	Height	Area%
1	11,575	34897103	877772	49,295
2	14,351	35895705	602694	50,705
Total		70792808	1480466	100,000

Figure S38. HPLC chromatogram of derivatized *rac*-(*E*)-4-phenylbut-3-en-2-amine using AD-H column (method: *n*-hept:*i*PrOH, 96:4, flow 0.7 mL/min, 25 ºC)

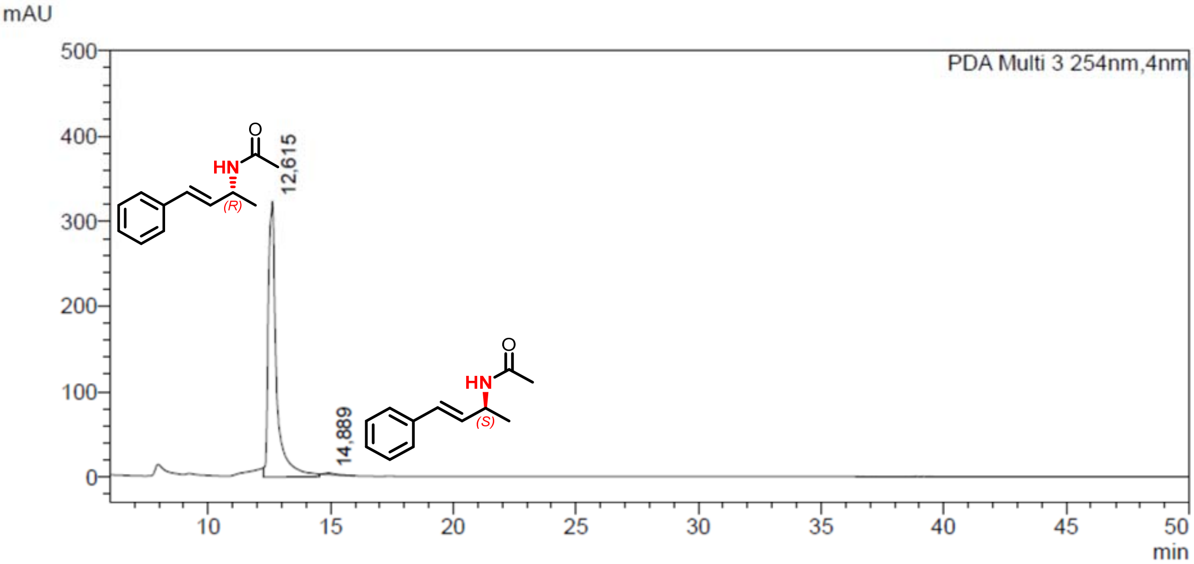

<Peak Table>

PDA Ch3 254nm

Peak#	Ret. Time	Area	Height	Area%
1	12,615	7542004	324476	99,349
2	14,889	49383	1798	0,651
Total		7591387	326274	100,000

Figure S39. HPLC chromatogram of derivatized (*R*)-**1c** from 0.2 mmol scale cascade with *rac*-**1a**, PQQ-DH, ArRmut11 transaminase and isopropylamine as the amine donor using AD-H column (method: *n*-hept:*i*PrOH, 96:4, flow 0.7 mL/min, 25 ºC)

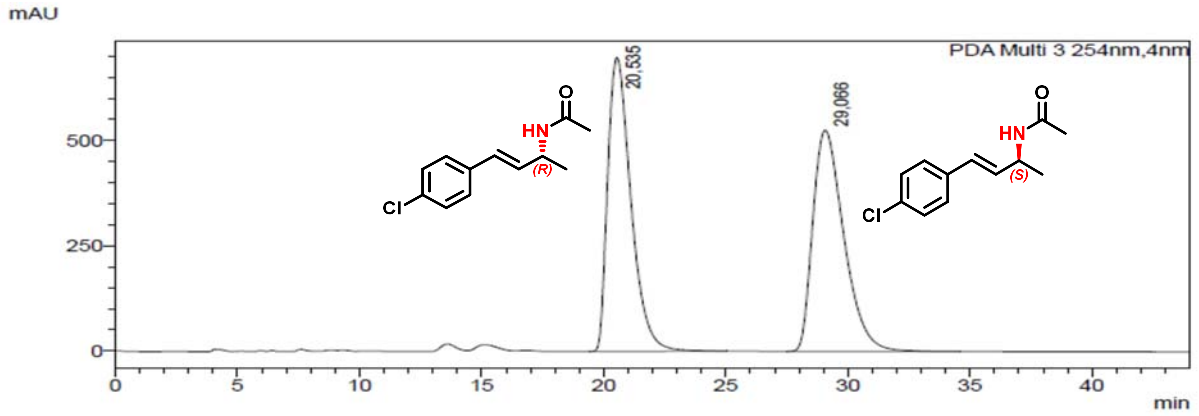

<Peak Table>

PDA Ch3 254nm

Peak#	Ret. Time	Area	Name	Area%
1	20,535	46243301		49,833
2	29,066	46553484		50,167
Total		92796785		100,000

Figure S40. HPLC chromatogram of derivatized *rac*-(*E*)-4-(4-chlorophenyl)but-3-en-2-amine using OJ column (method: *n*-hept:*i*PrOH, 90:10, flow 0.7 mL/min, 25 ºC)

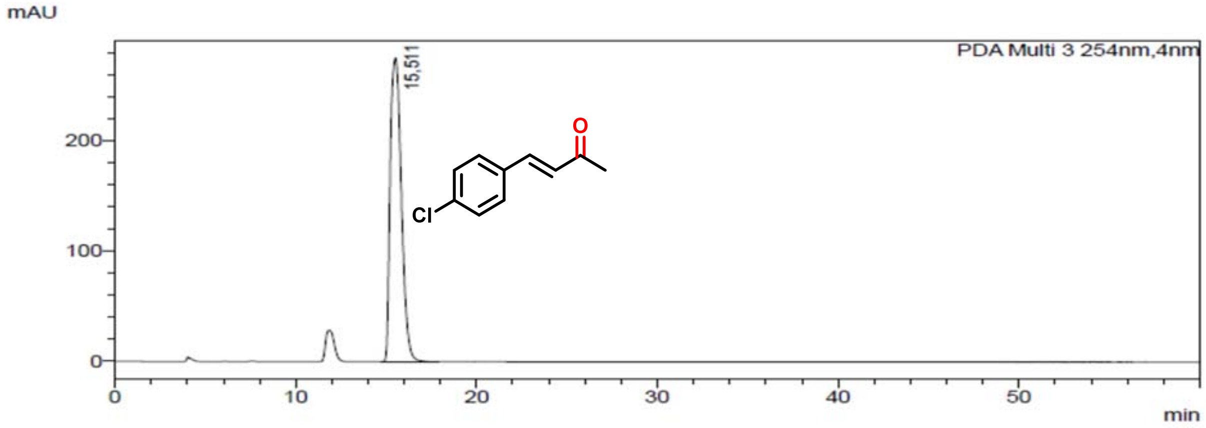

Figure S41. HPLC chromatogram of (*E*)-4-(4-chlorophenyl)but-3-en-2-one using OJ column (method: *n*-hept:*i*PrOH, 90:10, flow 0.7 mL/min, 25 ºC)

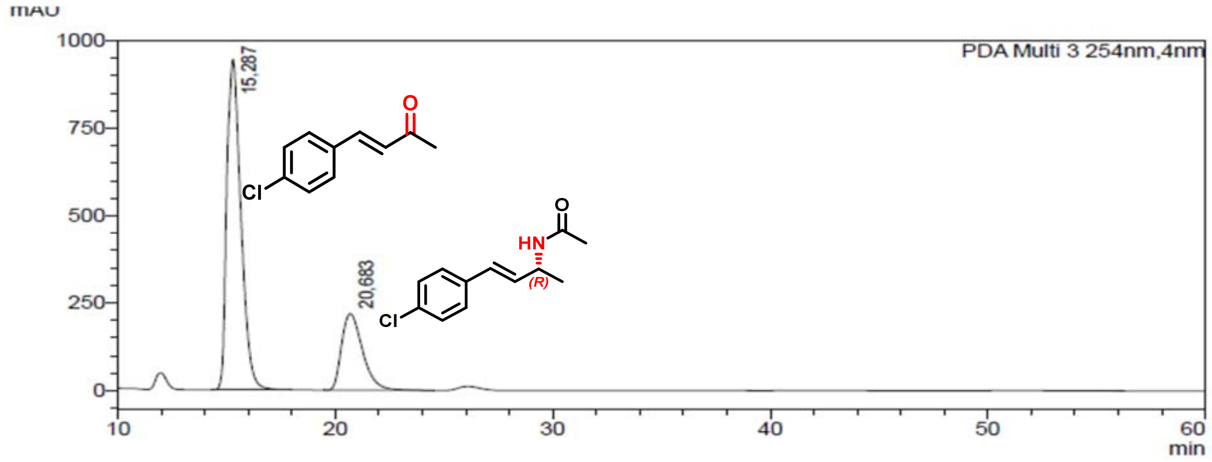

Figure S42. HPLC chromatogram of derivatized reaction mixture from ArRmut11 transaminase ((*R*)-selective transaminase) and isopropylamine as the amine donor using OJ column (method: *n*-hept:*i*PrOH, 90:10, flow 0.7 mL/min, 25 ºC)

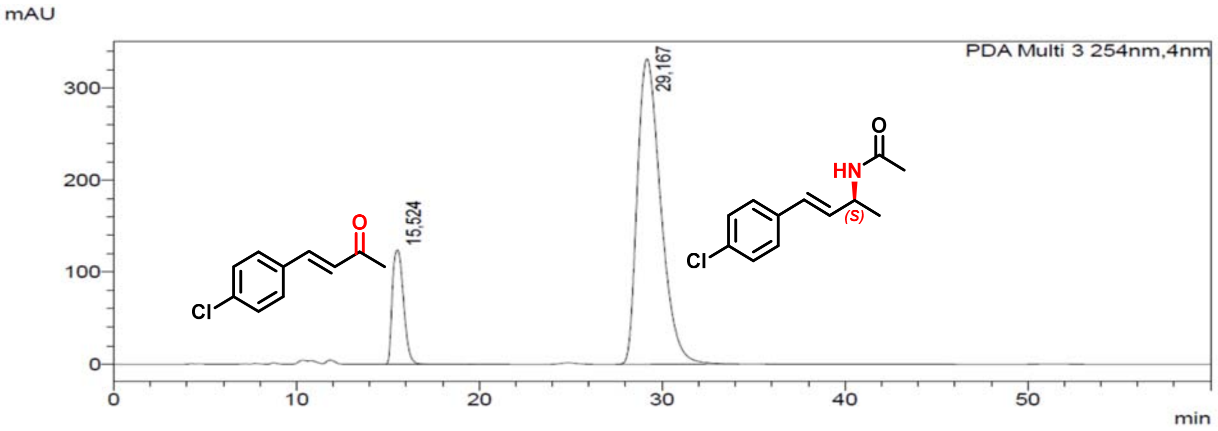

Figure S43. HPLC chromatogram of derivatized reaction mixture from pEG 205 transaminase ((*S*)-selective transaminase) and isopropylamine as the amine donor using OJ column (method: *n*-hept:*i*PrOH, 90:10, flow 0.7 mL/min, 25 ºC)

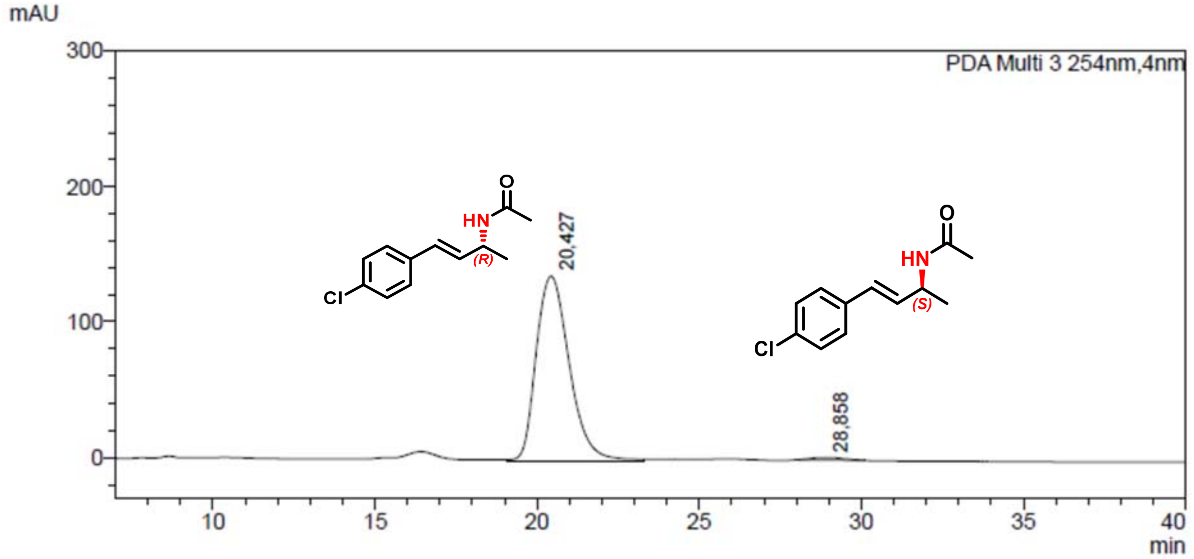

<Peak Table>

PDA Ch3 254nm

Peak#	Ret. Time	Area	Height	Area%
1	20,427	9760818	136299	98,632
2	28,858	135355	1865	1,368
Total		9896173	138164	100,000

Figure S44. HPLC chromatogram of derivatized (*R*)-**2c** from 0.2 mmol scale cascade with *rac*-**2a**, PQQ-DH, ArRmut11 transaminase and isopropylamine as the amine donor using OJ column (method: *n*-hept:*i*PrOH, 90:10, flow 0.7 mL/min, 25 ºC)

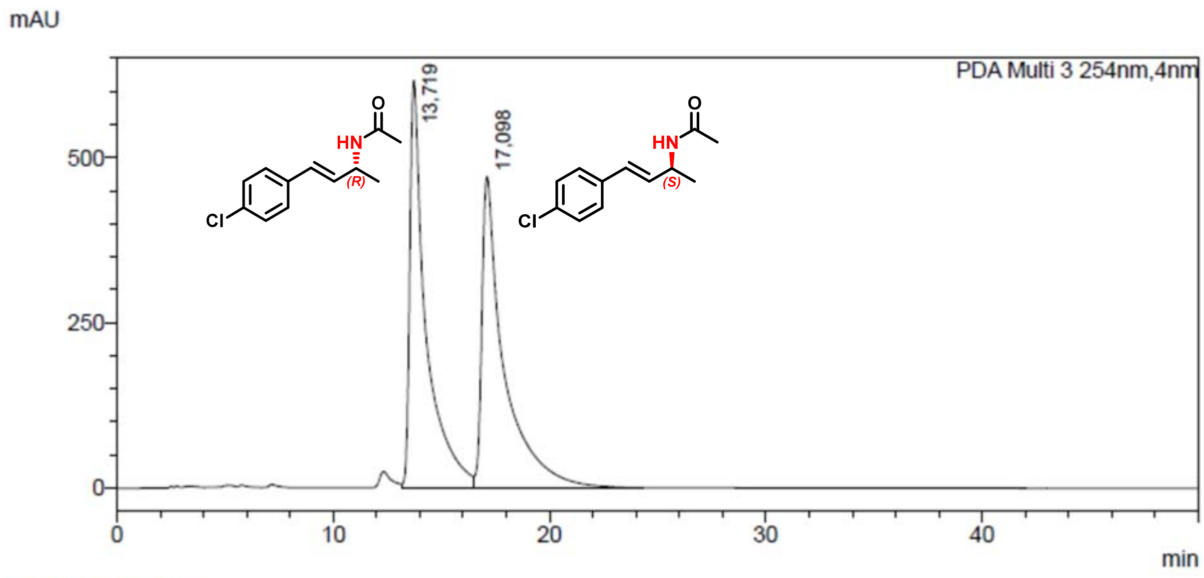

<Peak Table>

PDA Ch3 254nm

Peak#	Ret. Time	Area	Height	Area%
1	13,719	31937581	615378	49,031
2	17,098	33199733	470034	50,969
Total		65137315	1085412	100,000

Figure S45. HPLC chromatogram of derivatized *rac*-(*E*)-4-(4-chlorophenyl)but-3-en-2-amine using AD-H column (method: *n*-hept:*i*PrOH, 96:4, flow 0.7 mL/min, 25 ºC)

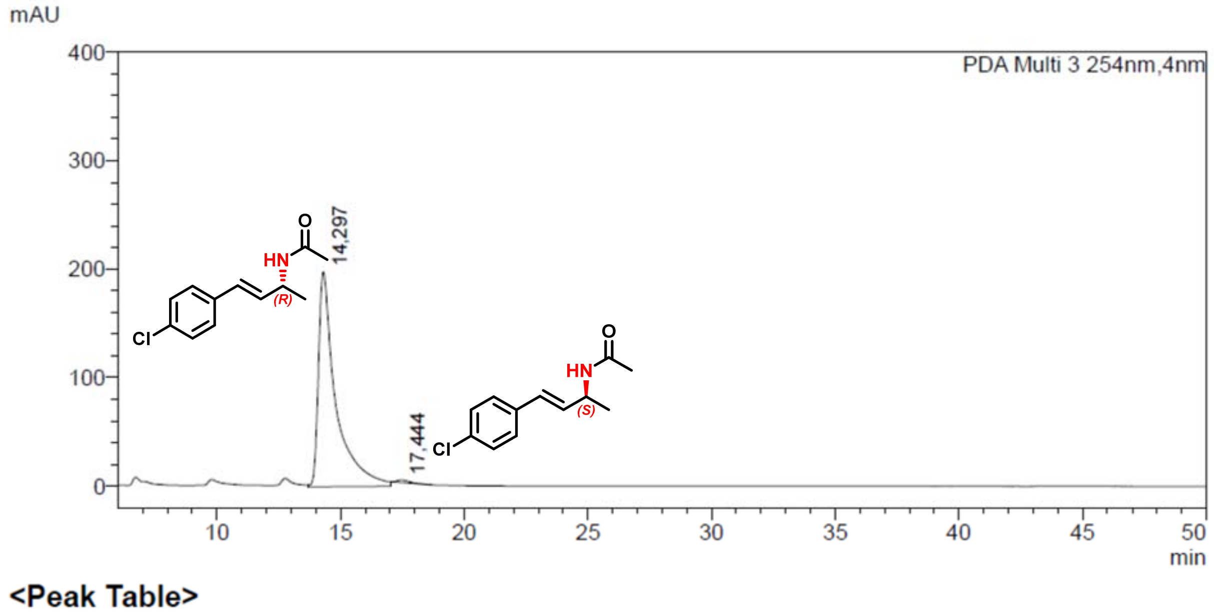

<Peak Table>

PDA Ch3 254nm

Peak#	Ret. Time	Area	Height	Area%
1	14,297	9633161	197944	99,139
2	17,444	83697	2429	0,861
Total		9716858	200374	100,000

Figure S46. HPLC chromatogram of derivatized (*R*)-**2c** from 0.2 mmol scale cascade with *rac*-**2a**, PQQ-DH, ArRmut11 transaminase and isopropylamine as the amine donor using AD-H column (method: *n*-hept:*i*PrOH, 96:4, flow 0.7 mL/min, 25 ºC)

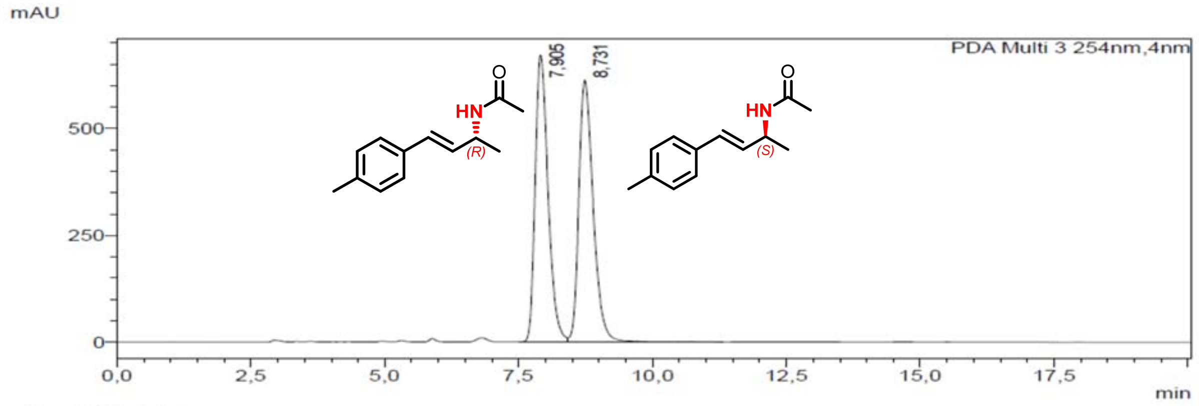

<Peak Table>

PDA Ch3 254nm

Peak#	Ret. Time	Area	Name	Area%
1	7,905	11237325		49,398
2	8,731	11511430		50,602
Total		22748755		100,000

Figure S47. HPLC chromatogram of derivatized *rac*-(*E*)-4-(*p*-tolyl)but-3-en-2-amine using OD-H column (method: *n*-hept:*i*PrOH, 90:10, flow 1.0 mL/min, 25 ºC)

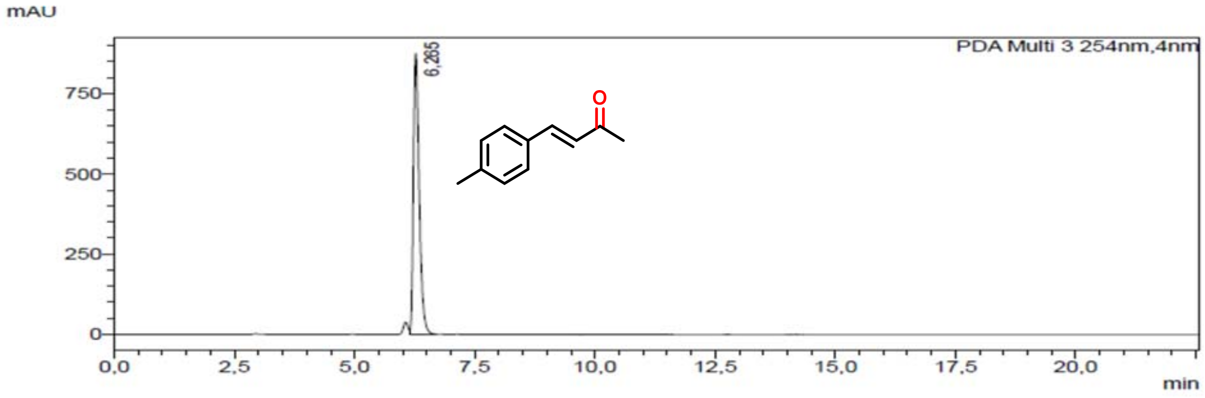

Figure S48. HPLC chromatogram of (*E*)-4-phenylbut-3-en-2-one using OD-H column (method: *n*-hept:*i*PrOH, 90:10, flow 1.0 mL/min, 25 ºC)

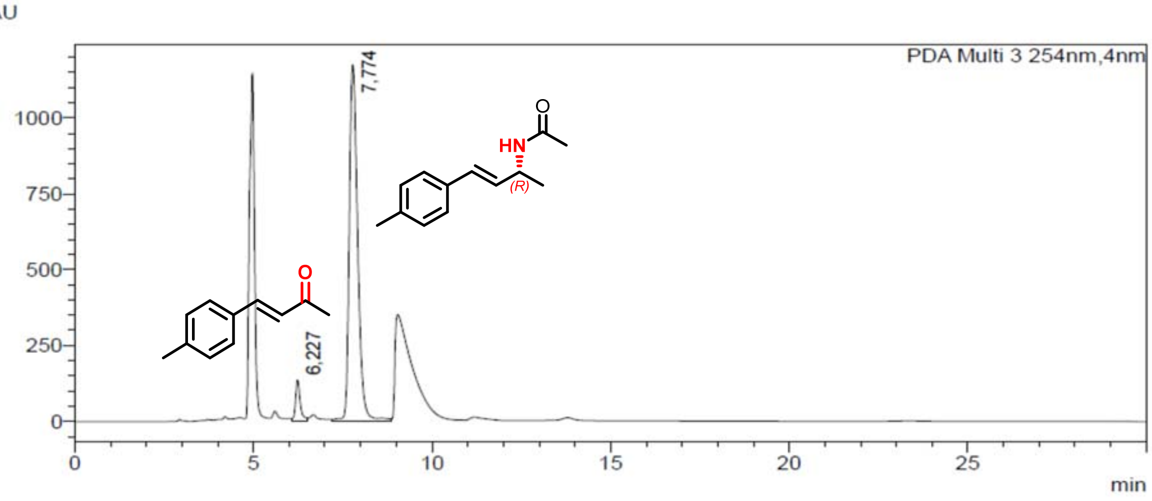

Figure S49. HPLC chromatogram of derivatized reaction mixture from ArRmut11 transaminase ((*R*)-selective transaminase) and (*R*)-phenylethylamine as the amine donor using OD-H column (method: *n*-hept:*i*PrOH, 90:10, flow 1.0 mL/min, 25 ºC), peak at 9.5 min is related to derivatized amine donor.

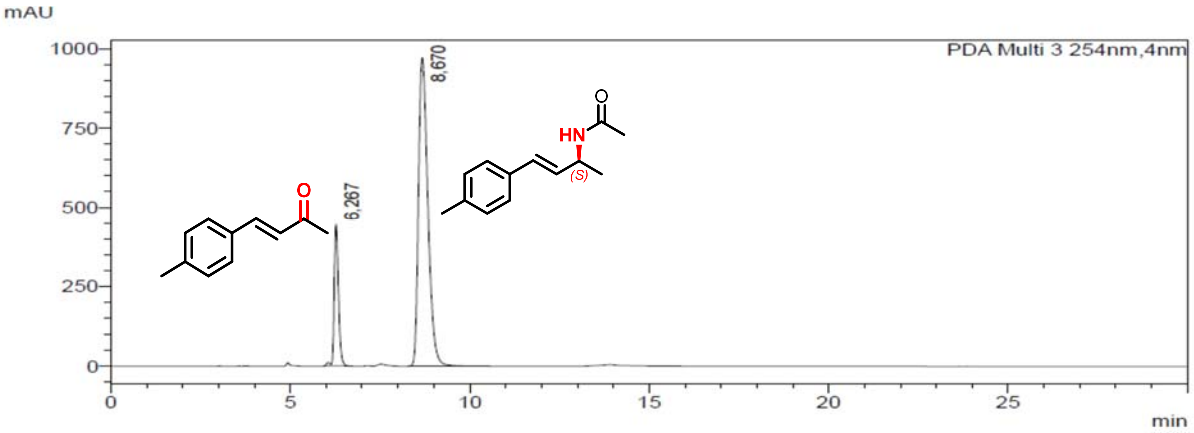

Figure S50. HPLC chromatogram of derivatized reaction mixture from pEG 205 transaminase ((*S*)-selective transaminase) and isopropylamine as the amine donor using OD-H column (method: *n*-hept:*i*PrOH, 90:10, flow 1.0 mL/min, 25 ºC)

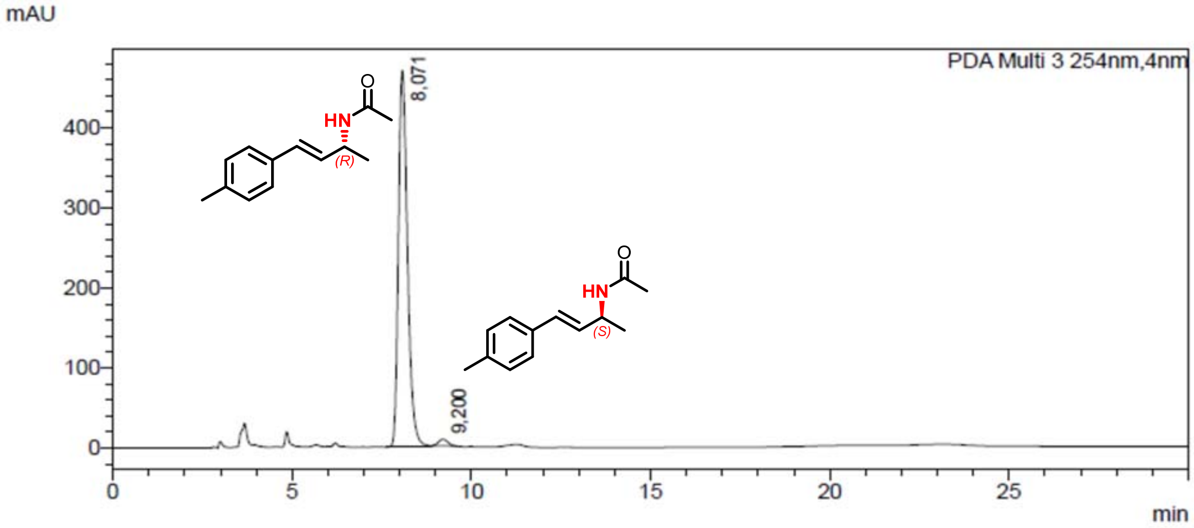

<Peak Table>

PDA Ch3 254nm

Peak#	Ret. Time	Area	Height	Area%
1	8,071	8293658	470955	98,297
2	9,200	143656	8087	1,703
Total		8437314	479042	100,000

Figure S51. HPLC chromatogram of derivatized (*R*)-**3c** from 0.2 mmol scale cascade with *rac*-**3a**, PQQ-DH, ArRmut11 transaminase and isopropylamine as the amine donor using OD-H column (method: *n*-hept:*i*PrOH, 90:10, flow 1.0 mL/min, 25 ºC)

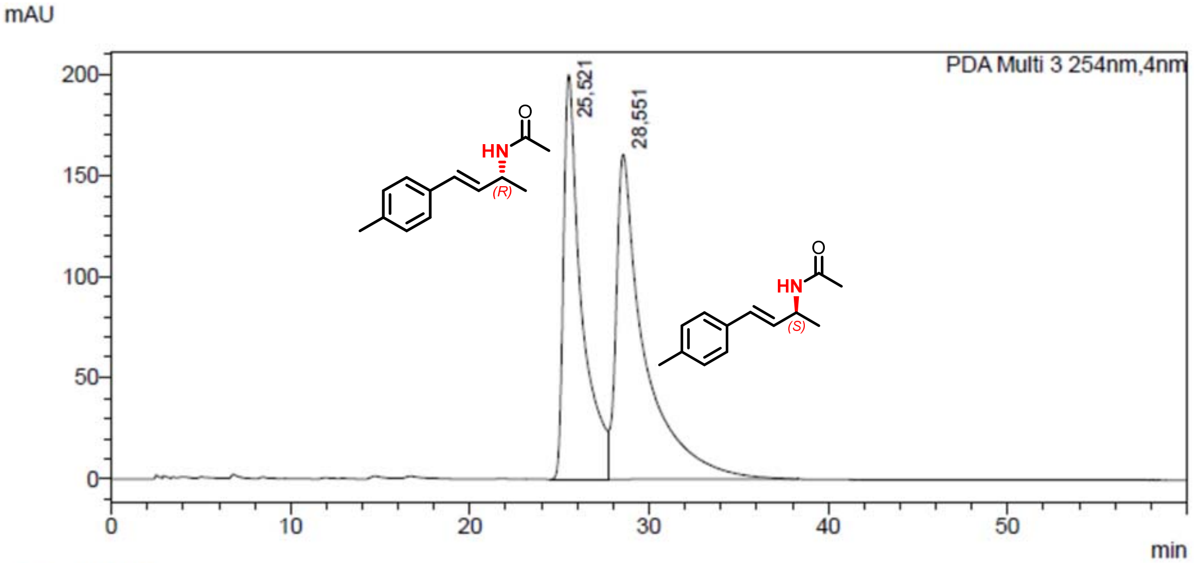

<Peak Table>

PDA Ch3 254nm

Peak#	Ret. Time	Area	Height	Area%
1	25,521	13899291	199790	44,019
2	28,551	17676691	160638	55,981
Total		31575982	360429	100,000

Figure S52. HPLC chromatogram of derivatized *rac*-(*E*)-4-(*p*-tolyl)but-3-en-2-amine using AD-H column (method: *n*-hept:*i*PrOH, 98:2, flow 0.7 mL/min, 25 ºC)

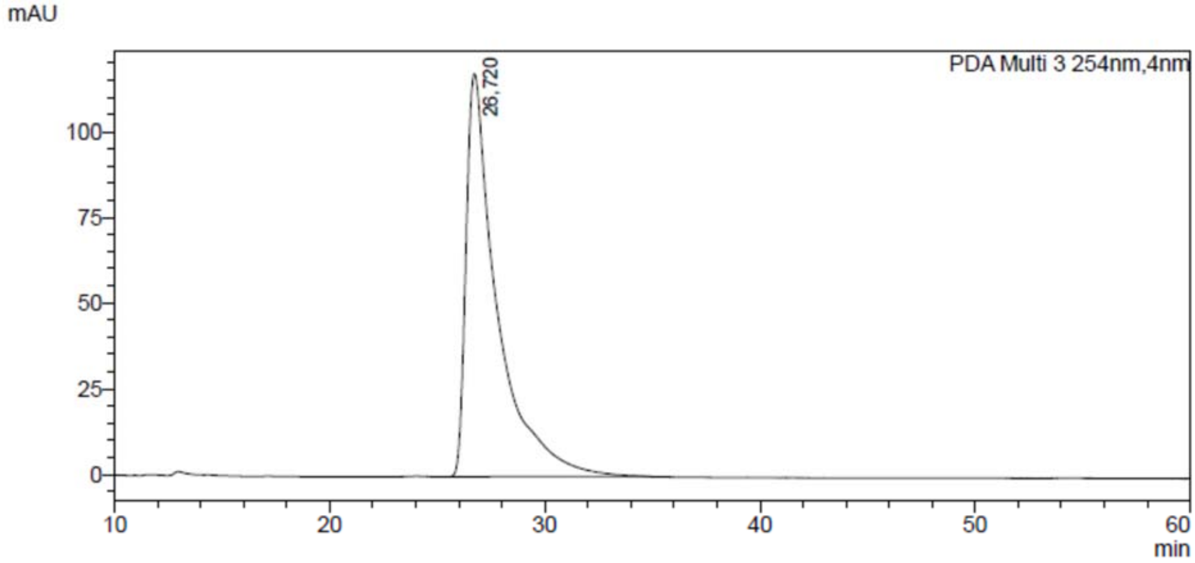

<Peak Table>

PDA Ch3 254nm

Peak#	Ret. Time	Area	Height	Area%
1	26,720	11794373	117513	100,000
Total		11794373	117513	100,000

Figure S53. HPLC chromatogram of derivatized (*R*)-**3c** from 0.2 mmol scale cascade with *rac*-**3a**, PQQ-DH, ArRmut11 transaminase and isopropylamine as the amine donor using AD-H column (method: *n*-hept:*i*PrOH, 98:2, flow 0.7 mL/min, 25 ºC)

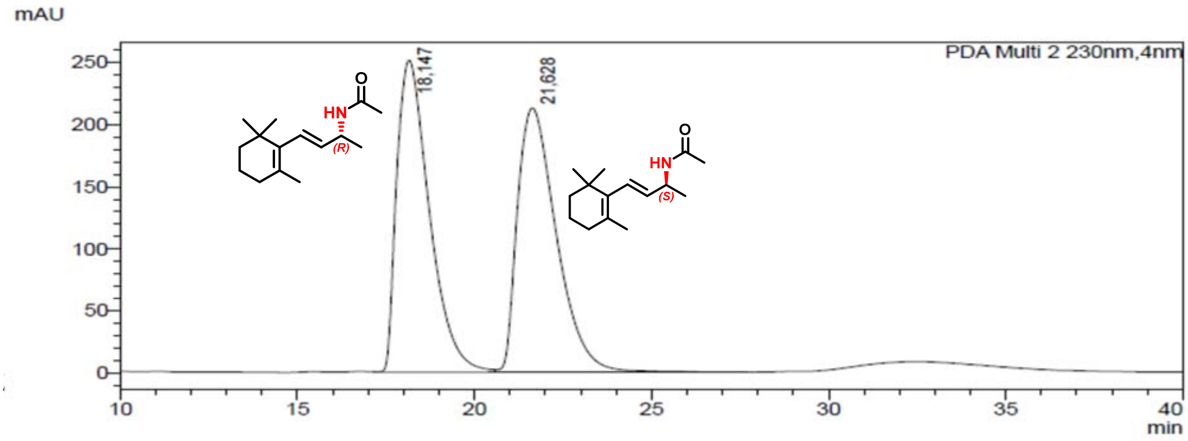

<Peak Table>

PDA Ch2 230nm

Peak#	Ret. Time	Area	Name	Area%
1	18,147	16138576		49,727
2	21,628	16315870		50,273
Total		32454446		100,000

Figure S54. HPLC chromatogram of derivatized *rac*-(*E*)-4-(2,6,6-trimethylcyclohex-1-en-1-yl)but-3-en-2-amine using OJ column (method: *n*-hept:*i*PrOH, 99:1, flow 0.7 mL/min, 25 ºC)

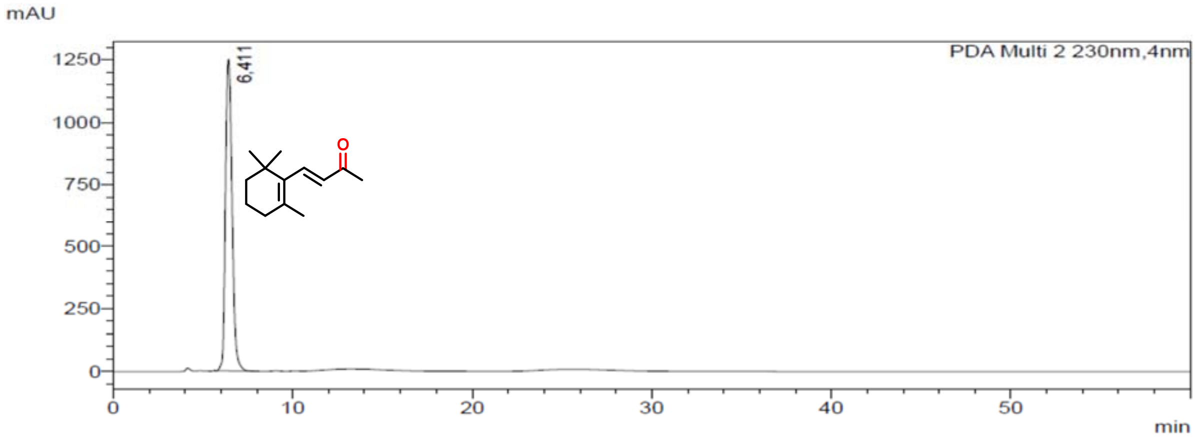

Figure S55. HPLC chromatogram of *β*-ionone using OJ column (method: *n*-hept:*i*PrOH, 99:1, flow 0.7 mL/min, 25 ºC)

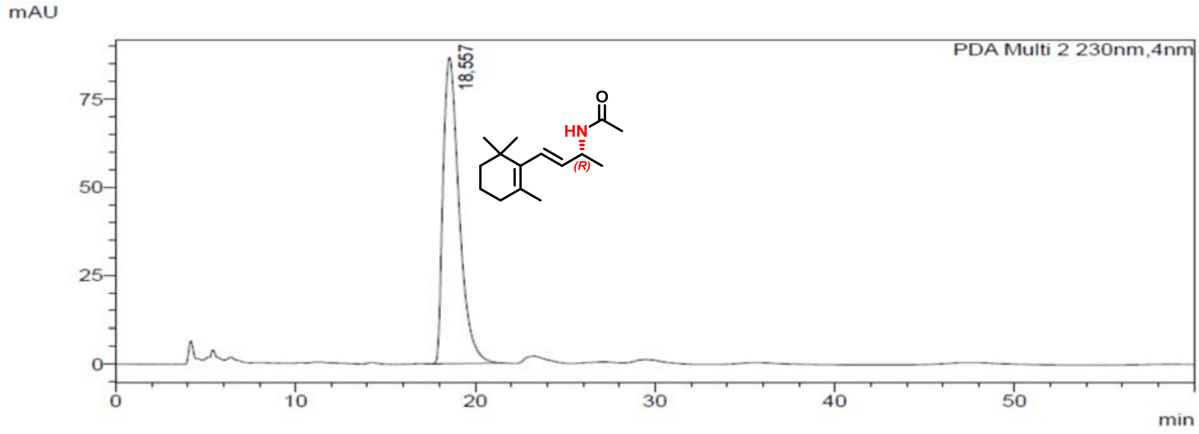

Figure S56. HPLC chromatogram of derivatized (*R*)-(*E*)-4-(2,6,6-trimethylcyclohex-1-en-1-yl)but-3-en-2-amine from ATA-117 upscaling using OJ column (method: *n*-hept:*i*PrOH, 99:1, flow 0.7 mL/min, 25 ºC)

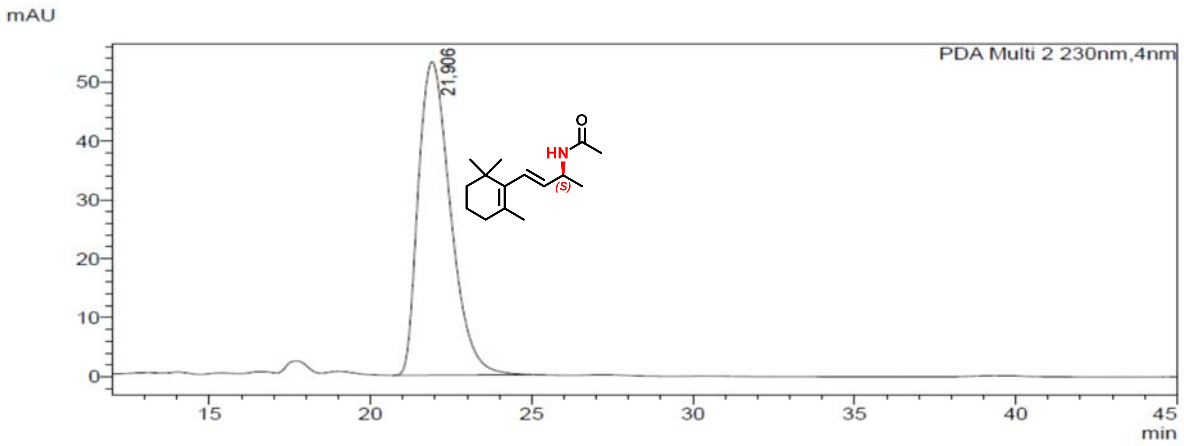

Figure S57. HPLC chromatogram of derivatized (*S*)-(*E*)-4-(2,6,6-trimethylcyclohex-1-en-1-yl)but-3-en-2-amine from pEG 209 transaminase using OJ column (method: *n*-hept:*i*PrOH, 99:1, flow 0.7 mL/min, 25 ºC)

4.4. GC chromatograms

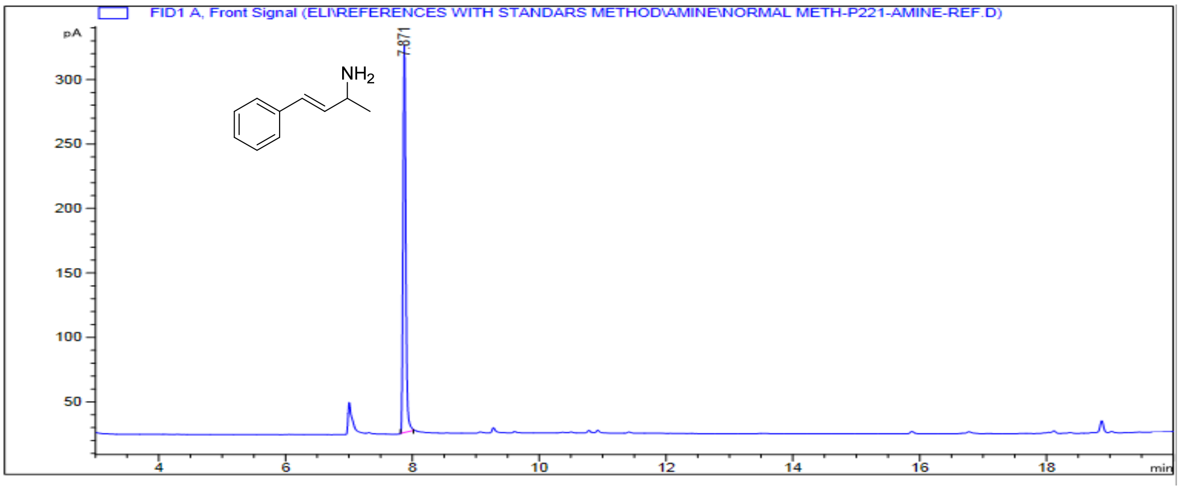

Figure S58. GC chromatogram of **1c** measured HP-5 column (30 m x 0.32 mm x 0.25 µm, J&W Scientific, Agilent Technologies) using He as carrier gas. Injector temperature: 250 °C; Injection volume: 5 µL; Flow rate: 0.7 mL/min; Temperature program (Standard Method): 100 °C, hold time 0.5 min, 10 °C/min to 300 °C, hold time 0 min.

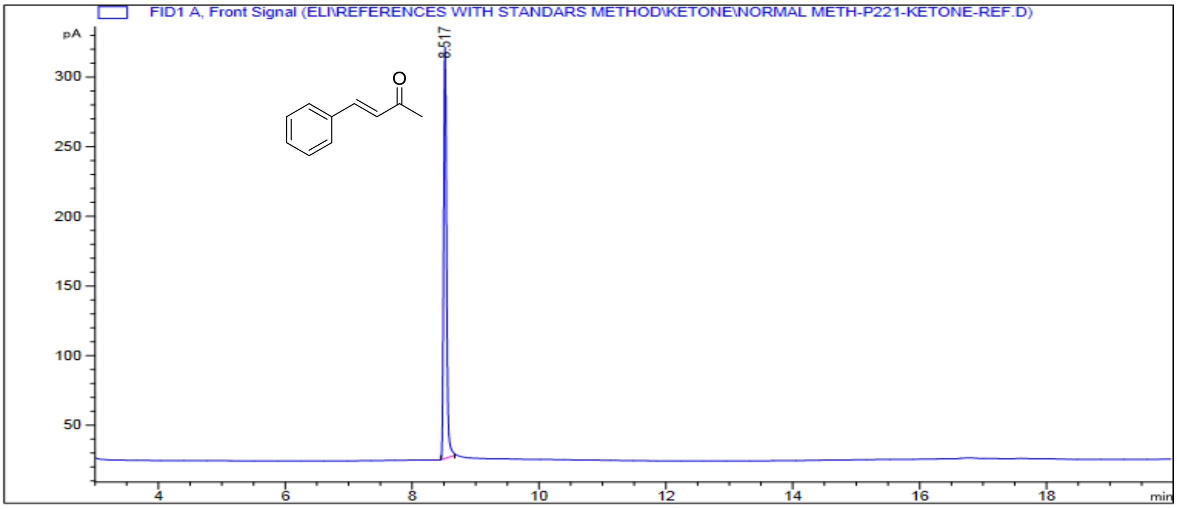

Figure S59. GC chromatogram of **1b** measured HP-5 column (30 m x 0.32 mm x 0.25 µm, J&W Scientific, Agilent Technologies) using He as carrier gas. Injector temperature: 250 °C; Injection volume: 5 µL; Flow rate: 0.7 mL/min; Temperature program (Standard Method): 100 °C, hold time 0.5 min, 10 °C/min to 300 °C, hold time 0 min.

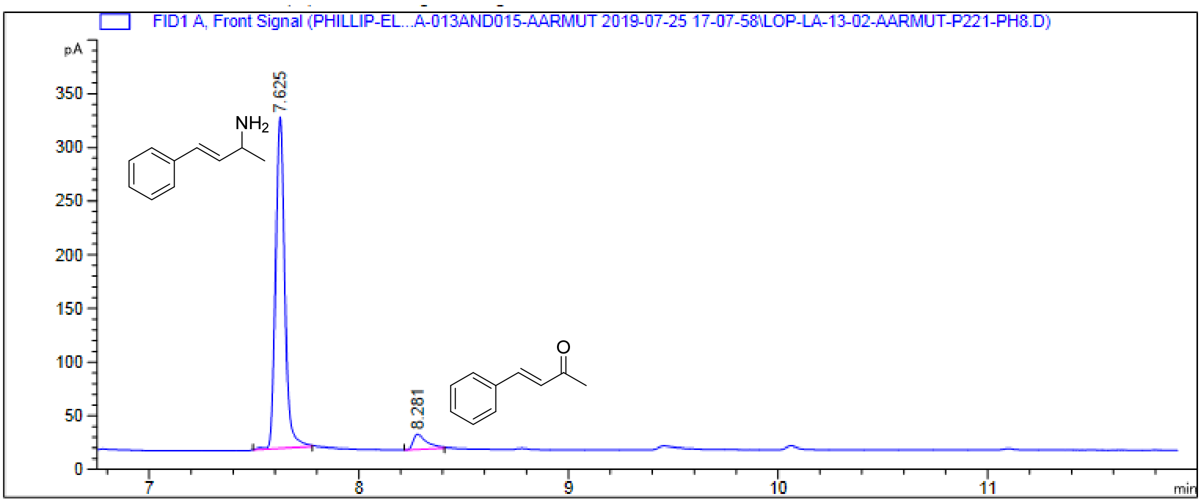


```
======================================================================
                         Area Percent Report
======================================================================

Sorted By             :      Signal
Multiplier            :      1.0000
Dilution              :      1.0000
Use Multiplier & Dilution Factor with ISTDs


Signal 1: FID1 A, Front Signal

Peak RetTime Type  Width     Area      Height     Area
  #   [min]        [min]   [pA*s]      [pA]        %
----|-------|----|-------|----------|----------|--------|
   1   7.625 BB    0.0477  942.78125  308.07324 93.82219
   2   8.281 BB    0.0660   62.07836   13.81876  6.17781

Totals :                  1004.85961  321.89200
```

Figure S60. GC chromatogram of amination of **1b** (50 mM) employing ArRmut11-ω-TA and (*R*)-1-phenylethyl amine (1.25 M) as donor measured on HP-5 column (30 m x 0.32 mm x 0.25 µm, J&W Scientific, Agilent Technologies) using He as carrier gas. Injector temperature: 250 °C; Injection volume: 5 µL; Flow rate: 0.7 mL/min; Temperature program (Standard Method): 100 °C, hold time 0.5 min, 10 °C/min to 300 °C, hold time 0 min.

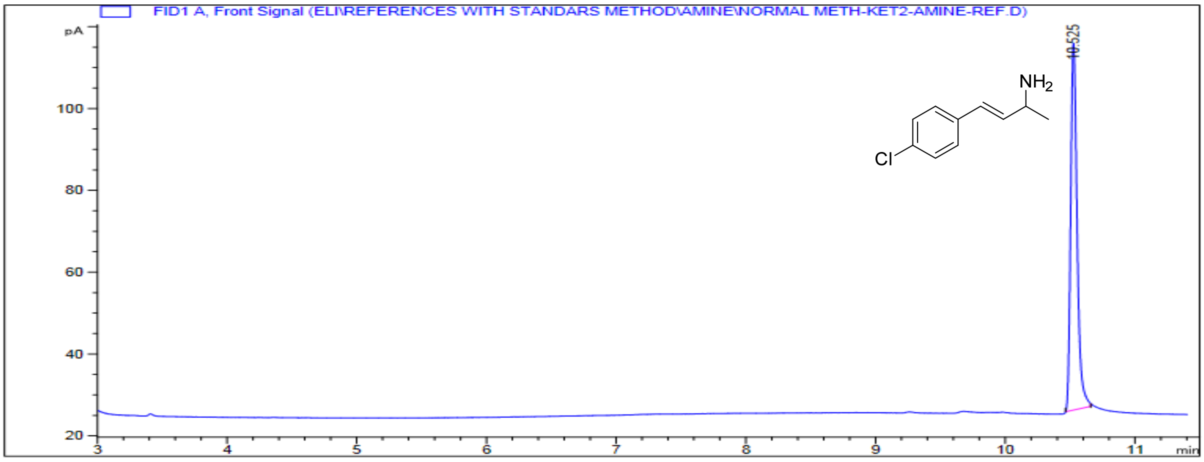

Figure S61. GC chromatogram of **2c** measured HP-5 column (30 m x 0.32 mm x 0.25 µm, J&W Scientific, Agilent Technologies) using He as carrier gas. Injector temperature: 250 °C; Injection volume: 5 µL; Flow rate: 0.7 mL/min; Temperature program (Standard Method): 100 °C, hold time 0.5 min, 10 °C/min to 300 °C, hold time 0 min.

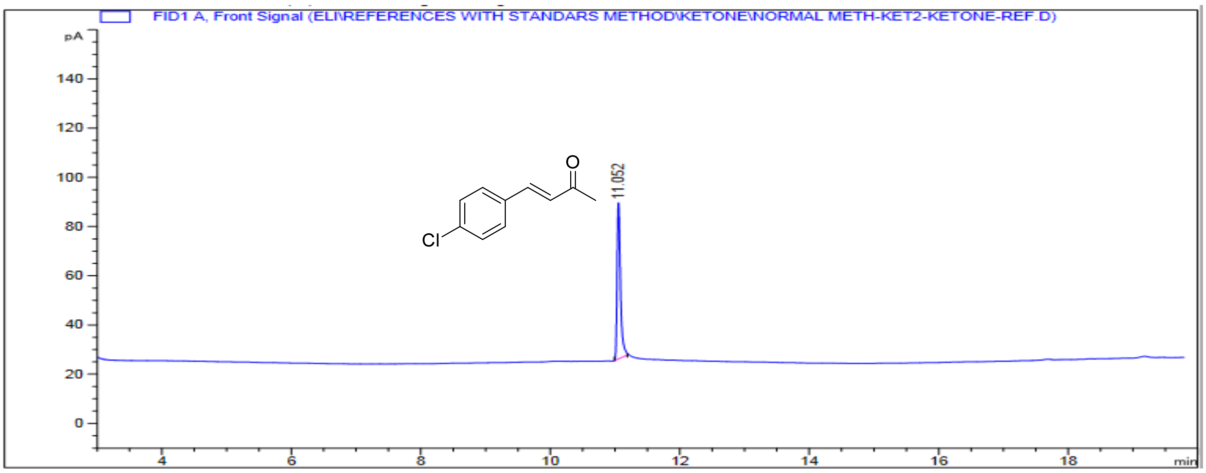

Figure S62. GC chromatogram of **2b** measured HP-5 column (30 m x 0.32 mm x 0.25 µm, J&W Scientific, Agilent Technologies) using He as carrier gas. Injector temperature: 250 °C; Injection volume: 5 µL; Flow rate: 0.7 mL/min; Temperature program (Standard Method): 100 °C, hold time 0.5 min, 10 °C/min to 300 °C, hold time 0 min.

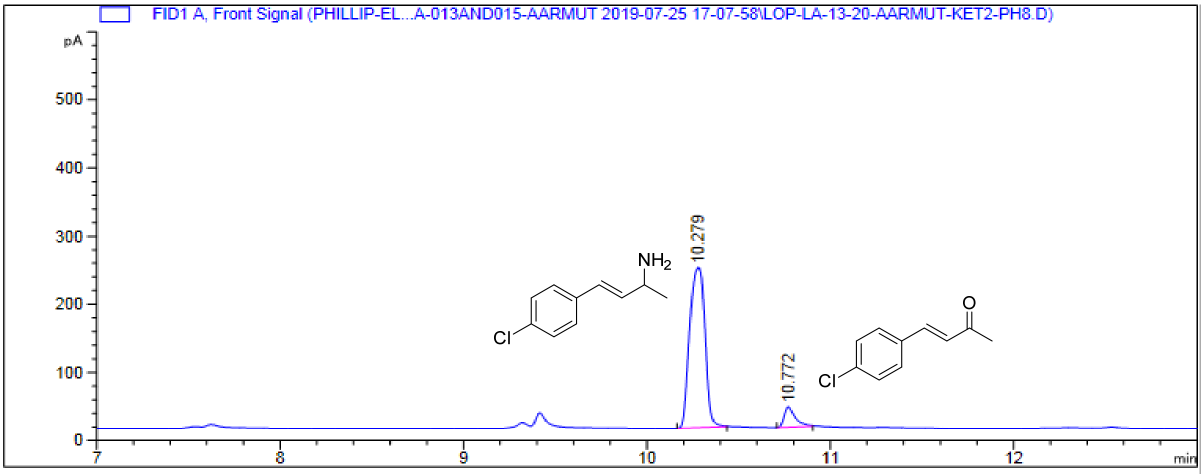


```
=====================================================================
                         Area Percent Report
=====================================================================

Sorted By             :      Signal
Multiplier            :      1.0000
Dilution              :      1.0000
Use Multiplier & Dilution Factor with ISTDs


Signal 1: FID1 A, Front Signal

Peak RetTime Type  Width     Area      Height     Area
  #   [min]        [min]   [pA*s]      [pA]        %
----|-------|----|-------|----------|----------|--------|
   1  10.279 BB    0.0700 1358.44836  234.49658 91.46778
   2  10.772 BB    0.0620  126.71758   30.01801  8.53222

Totals :                  1485.16595  264.51459


=====================================================================
```

Figure S63. GC chromatogram of amination of **2b** (50 mM) employing ArRmut11-ω-TA and (*R*)-1-phenylethyl amine (1.25 M) as donor measured on HP-5 column (30 m x 0.32 mm x 0.25 µm, J&W Scientific, Agilent Technologies) using He as carrier gas. Injector temperature: 250 °C; Injection volume: 5 µL; Flow rate: 0.7 mL/min; Temperature program (Standard Method): 100 °C, hold time 0.5 min, 10 °C/min to 300 °C, hold time 0 min.

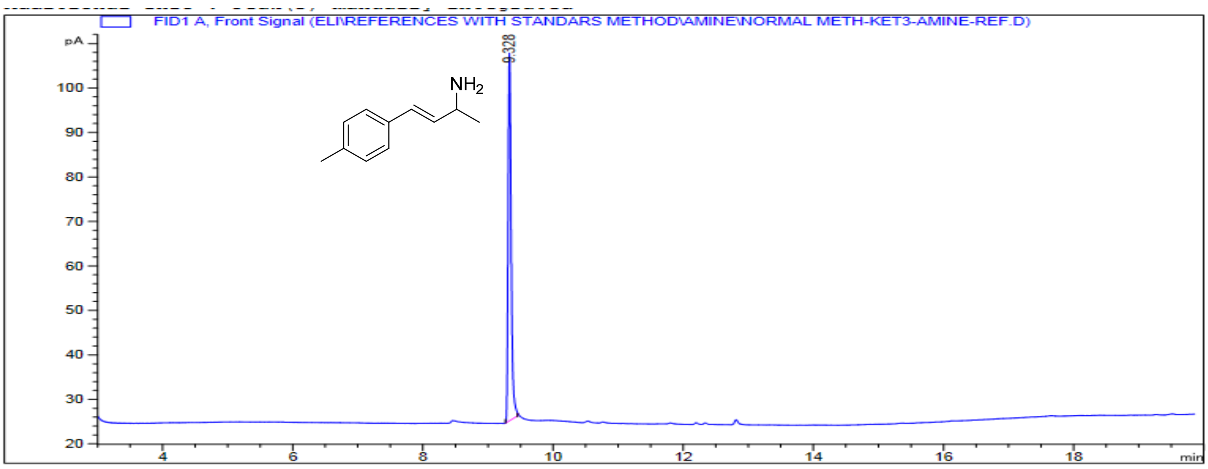

Figure S64. GC chromatogram of **3c** measured HP-5 column (30 m x 0.32 mm x 0.25 µm, J&W Scientific, Agilent Technologies) using He as carrier gas. Injector temperature: 250 °C; Injection volume: 5 µL; Flow rate: 0.7 mL/min; Temperature program (Standard Method): 100 °C, hold time 0.5 min, 10 °C/min to 300 °C, hold time 0 min.

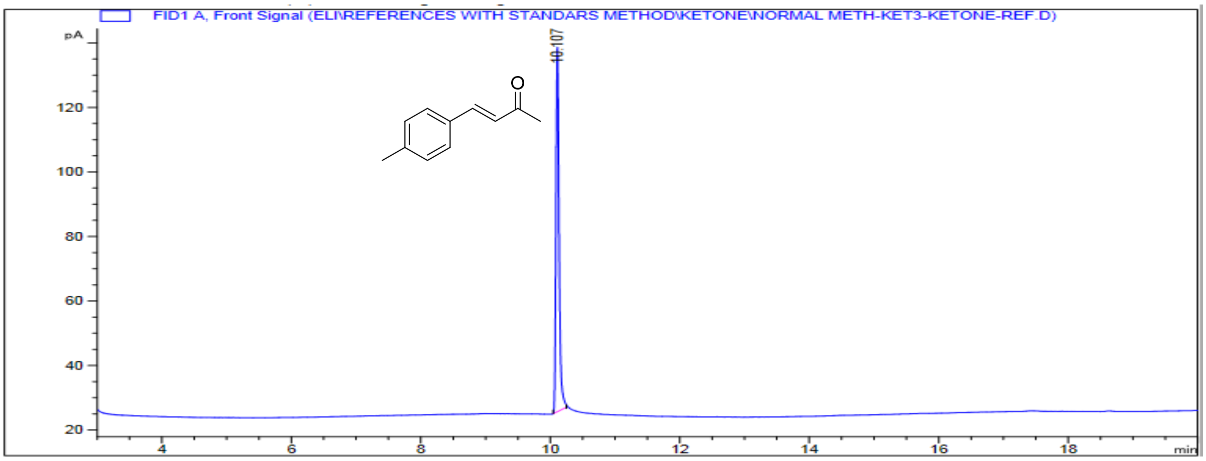

Figure S65. GC chromatogram of **3b** measured HP-5 column (30 m x 0.32 mm x 0.25 µm, J&W Scientific, Agilent Technologies) using He as carrier gas. Injector temperature: 250 °C; Injection volume: 5 µL; Flow rate: 0.7 mL/min; Temperature program (Standard Method): 100 °C, hold time 0.5 min, 10 °C/min to 300 °C, hold time 0 min.

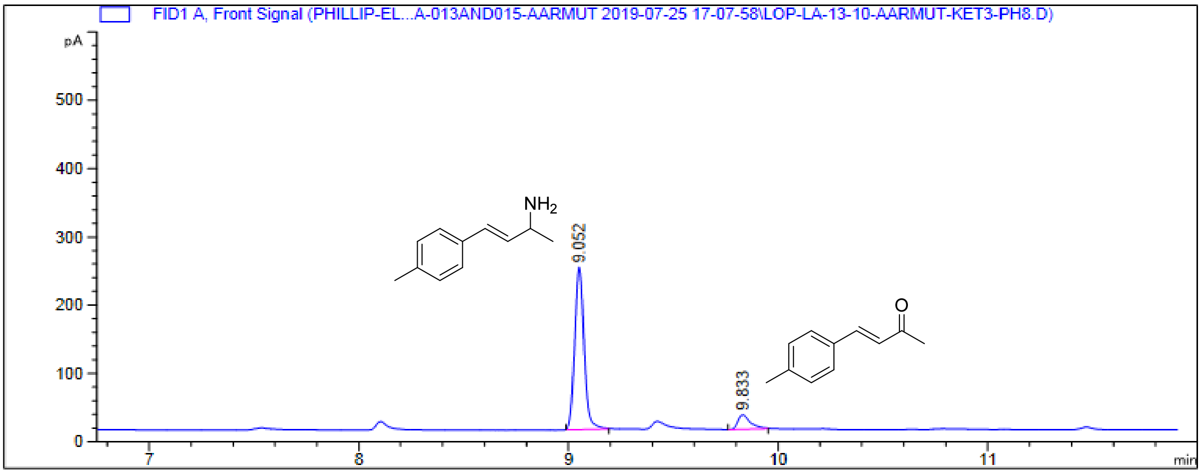


```
=====================================================================
                        Area Percent Report
=====================================================================

Sorted By             :      Signal
Multiplier            :      1.0000
Dilution              :      1.0000
Use Multiplier & Dilution Factor with ISTDs


Signal 1: FID1 A, Front Signal

Peak RetTime Type  Width      Area       Height      Area
  #    [min]        [min]    [pA*s]       [pA]         %
----|-------|----|-------|----------|----------|--------|
   1   9.052 BB    0.0477  741.98438  236.95818 89.63274
   2   9.833 BB    0.0625   85.82067   20.83266 10.36726

Totals :                   827.80505  257.79083

=====================================================================
```

Figure S66. GC chromatogram of amination of **3b** (50 mM) employing ArRmut11-ω-TA and (*R*)-1-phenylethyl amine (1.25 M) as donor measured on HP-5 column (30 m x 0.32 mm x 0.25 µm, J&W Scientific, Agilent Technologies) using He as carrier gas. Injector temperature: 250 °C; Injection volume: 5 µL; Flow rate: 0.7 mL/min; Temperature program (Standard Method): 100 °C, hold time 0.5 min, 10 °C/min to 300 °C, hold time 0 min.

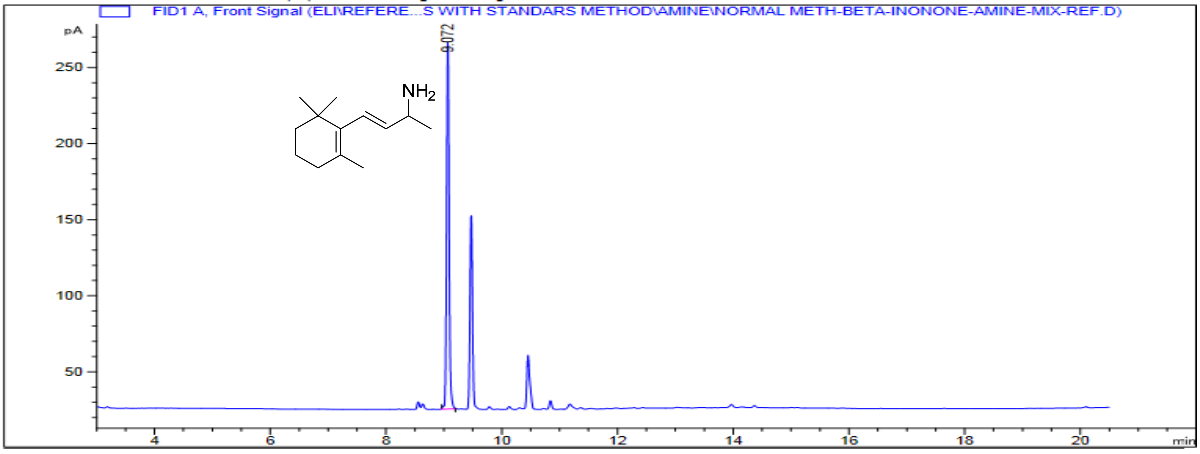

Figure S67. GC chromatogram of **4c** measured HP-5 column (30 m x 0.32 mm x 0.25 µm, J&W Scientific, Agilent Technologies) using He as carrier gas. Injector temperature: 250 °C; Injection volume: 5 µL; Flow rate: 0.7 mL/min; Temperature program (Standard Method): 100 °C, hold time 0.5 min, 10 °C/min to 300 °C, hold time 0 min.

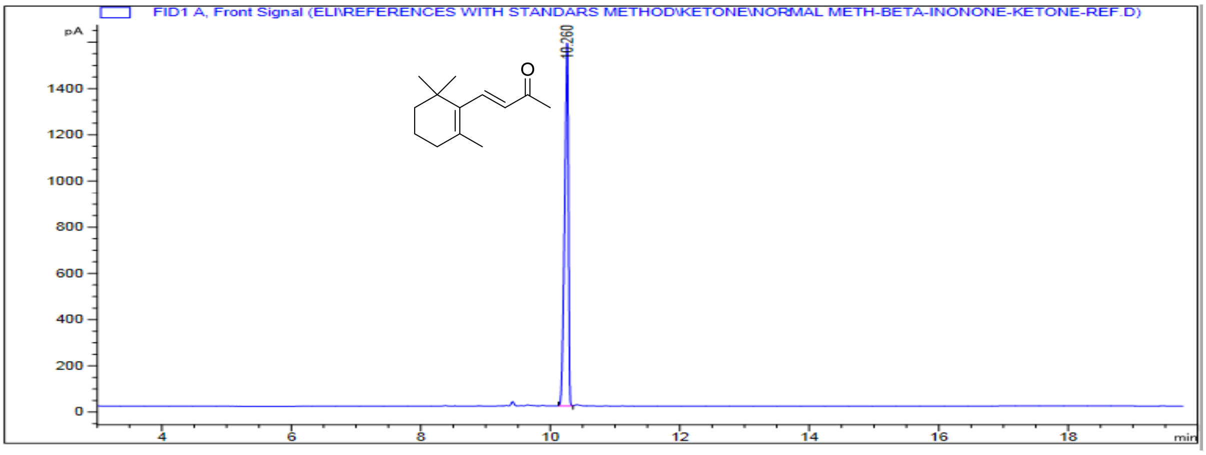

Figure S68. GC chromatogram of **4b** measured HP-5 column (30 m x 0.32 mm x 0.25 µm, J&W Scientific, Agilent Technologies) using He as carrier gas. Injector temperature: 250 °C; Injection volume: 5 µL; Flow rate: 0.7 mL/min; Temperature program (Standard Method): 100 °C, hold time 0.5 min, 10 °C/min to 300 °C, hold time 0 min.

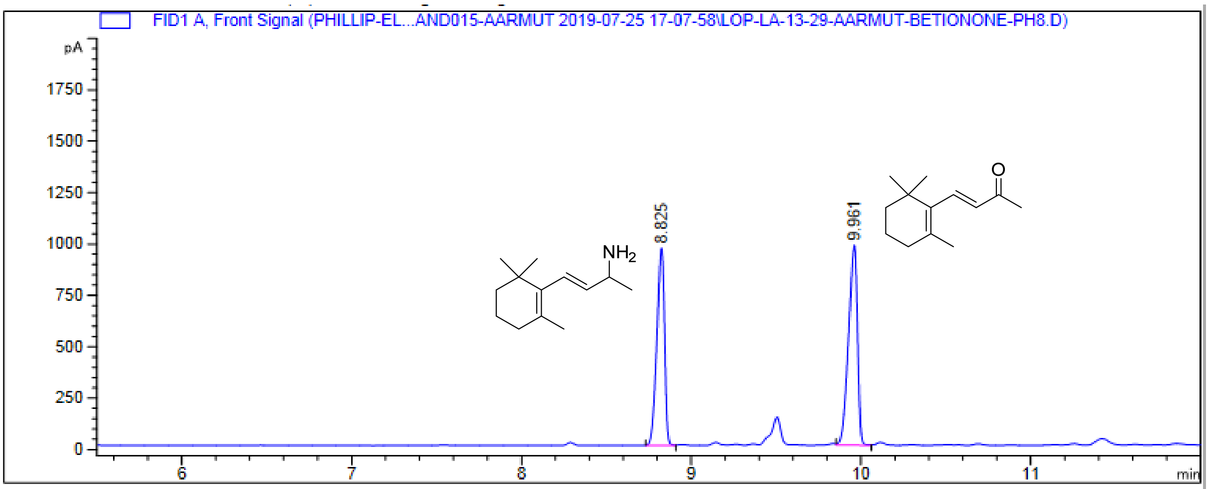


```
=====================================================================
                         Area Percent Report
=====================================================================

Sorted By              :      Signal
Multiplier             :      1.0000
Dilution               :      1.0000
Use Multiplier & Dilution Factor with ISTDs


Signal 1: FID1 A, Front Signal

Peak RetTime Type  Width     Area      Height     Area
  #   [min]        [min]   [pA*s]      [pA]        %
----|-------|----|-------|----------|----------|--------|
   1   8.825 BV    0.0497 3037.38647  960.74481 46.01673
   2   9.961 VV    0.0539 3563.22705  972.78729 53.98327

Totals :                  6600.61353 1933.53210


=====================================================================
```

Figure S69. GC chromatogram of amination of **4b** (50 mM) employing ArRmut11-ω-TA and (*R*)-1-phenylethyl amine (1.25 M) as donor measured on HP-5 column (30 m x 0.32 mm x 0.25 µm, J&W Scientific, Agilent Technologies) using He as carrier gas. Injector temperature: 250 °C; Injection volume: 5 µL; Flow rate: 0.7 mL/min; Temperature program (Standard Method): 100 °C, hold time 0.5 min, 10 °C/min to 300 °C, hold time 0 min.

4.5. GC-MS chromatograms

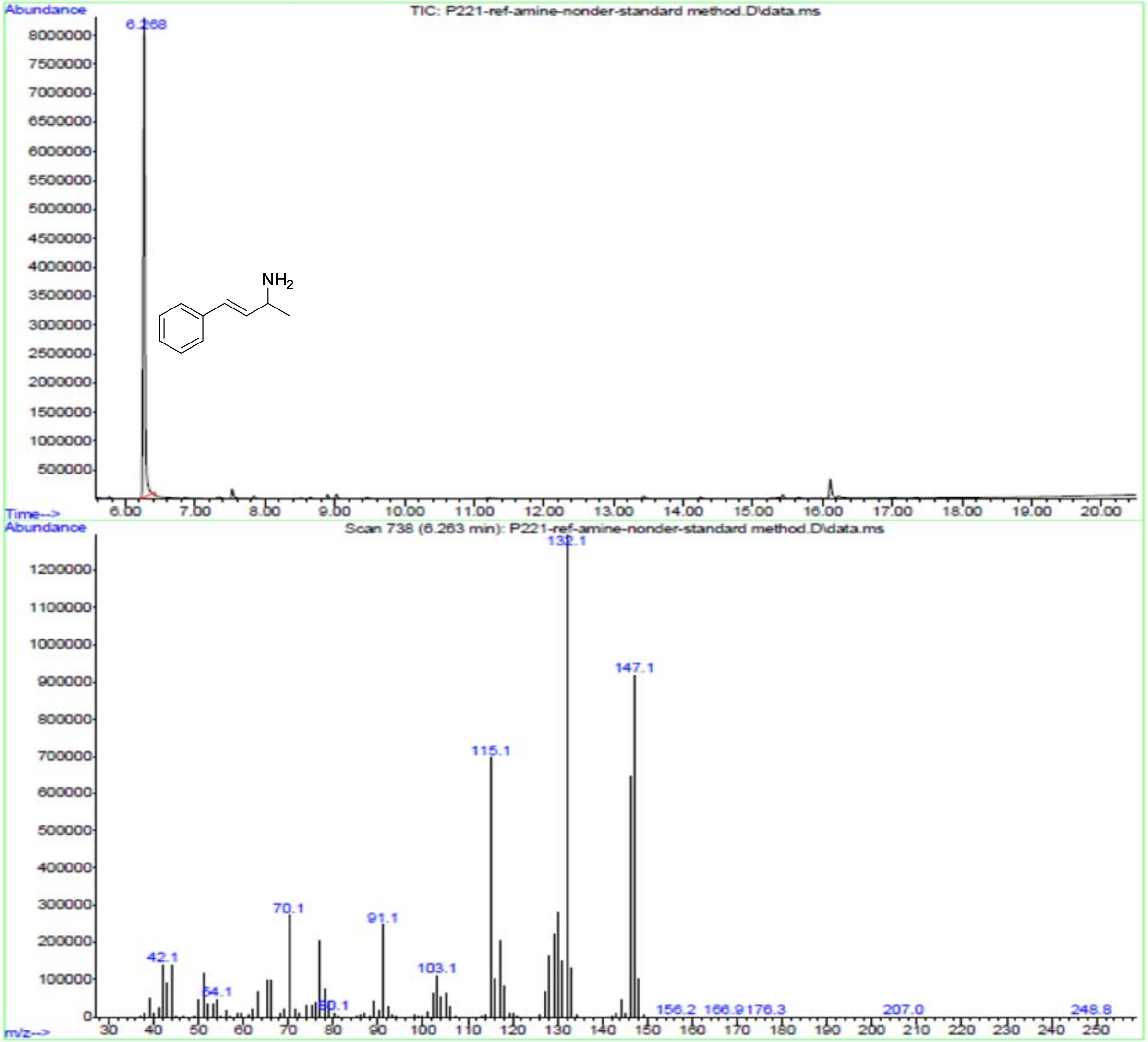

Figure S70. GC-MS chromatogram of **1c** measured on HP-5MS column (5% phenylmethylsiloxane, 30 m x 0.20 mm x 0.25 µm, J&W Scientific, Agilent Technologies) using He as carrier gas. Injector temperature: 250 °C; split ratio: 90:1; Injection volume: 5 µL; Flow rate: 0.7 mL/min; Temperature program (standard method): 100 °C, hold time 0.5 min, 10 °C/min to 300 °C, hold time 0 min; EI mode, energy 70 eV, MS Source: 230 °C, MS Quadrupole: 150 °C.

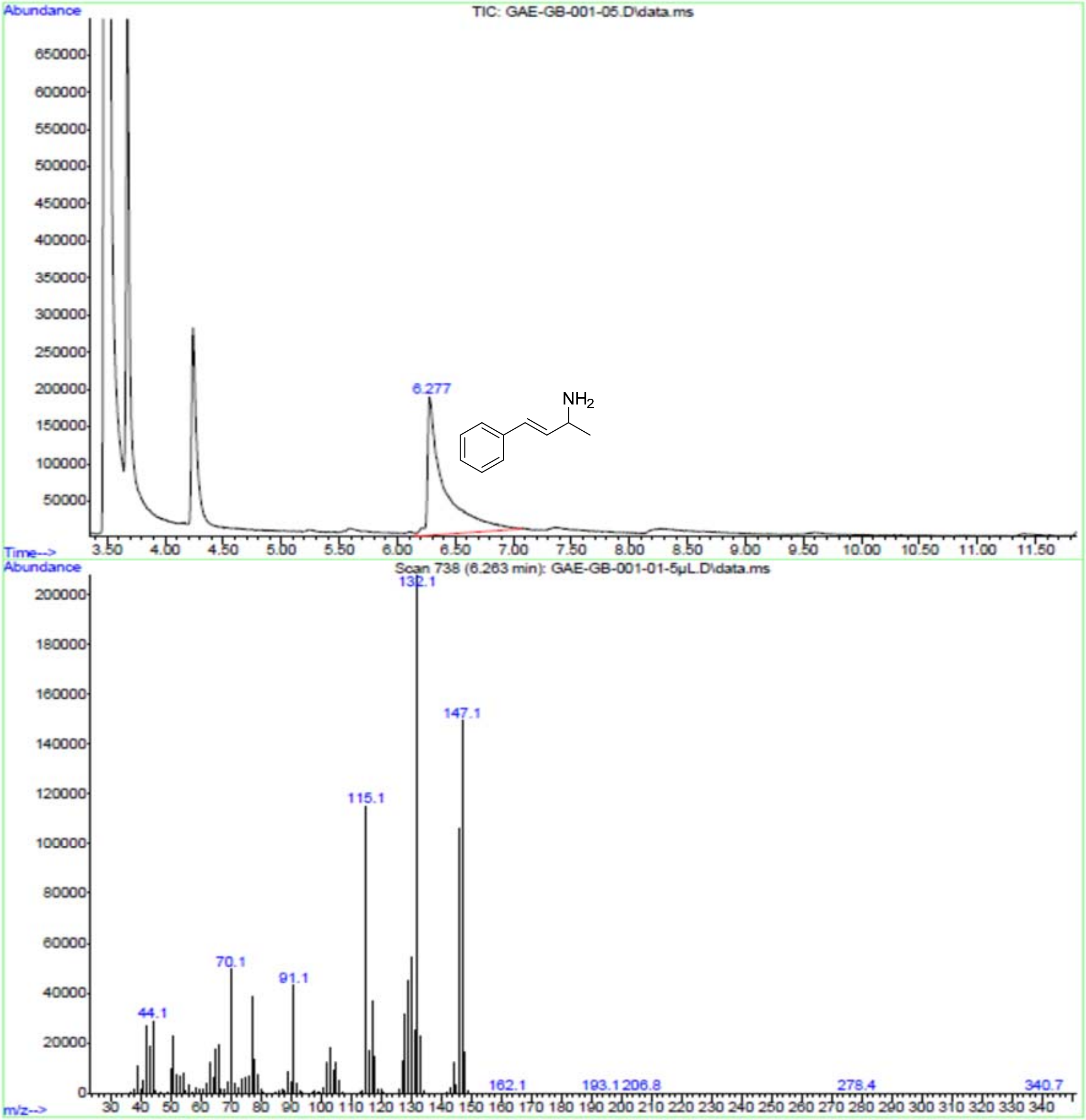

Figure S71. GC-MS chromatogram of amination of **1a** (10 mM) employing ArRmut11-ω-TA and (*R*)-1-phenylethyl amine (250 mM) as donor measured on HP-5MS column (5% phenylmethylsiloxane, 30 m x 0.20 mm x 0.25 µm, J&W Scientific, Agilent Technologies) using He as carrier gas. Injector temperature: 250 °C; split ratio: 90:1; Injection volume: 5 µL; Flow rate: 0.7 mL/min; Temperature program (standard method): 100 °C, hold time 0.5 min, 10 °C/min to 300 °C, hold time 0 min; EI mode, energy 70 eV, MS Source: 230 °C, MS Quadrupole: 150 °C.

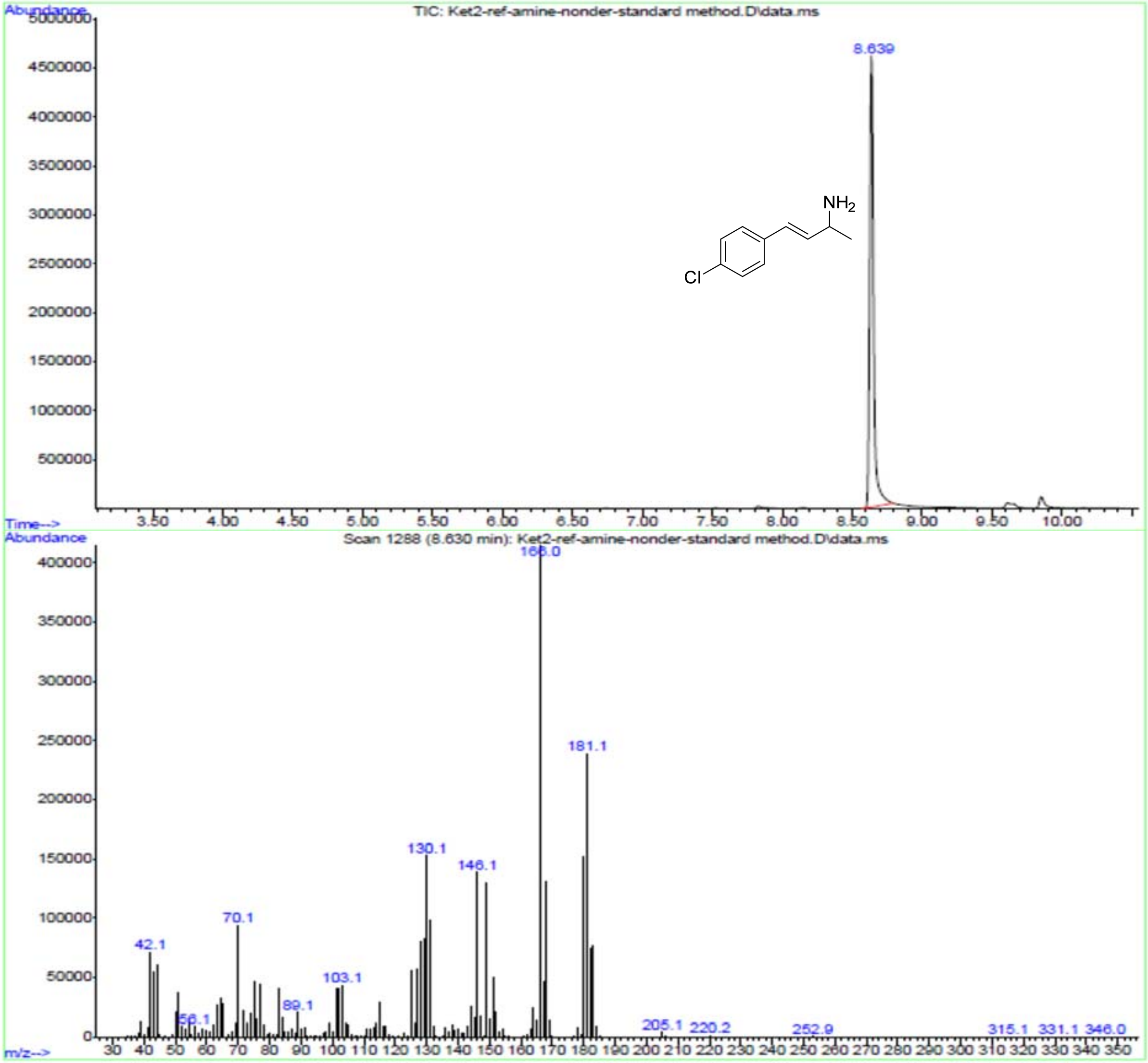

Figure S72. GC-MS chromatogram of **2c** measured on HP-5MS column (5% phenylmethylsiloxane, 30 m x 0.20 mm x 0.25 µm, J&W Scientific, Agilent Technologies) using He as carrier gas. Injector temperature: 250 °C; split ratio: 90:1; Injection volume: 5 µL; Flow rate: 0.7 mL/min; Temperature program (standard method): 100 °C, hold time 0.5 min, 10 °C/min to 300 °C, hold time 0 min; EI mode, energy 70 eV, MS Source: 230 °C, MS Quadrupole: 150 °C.

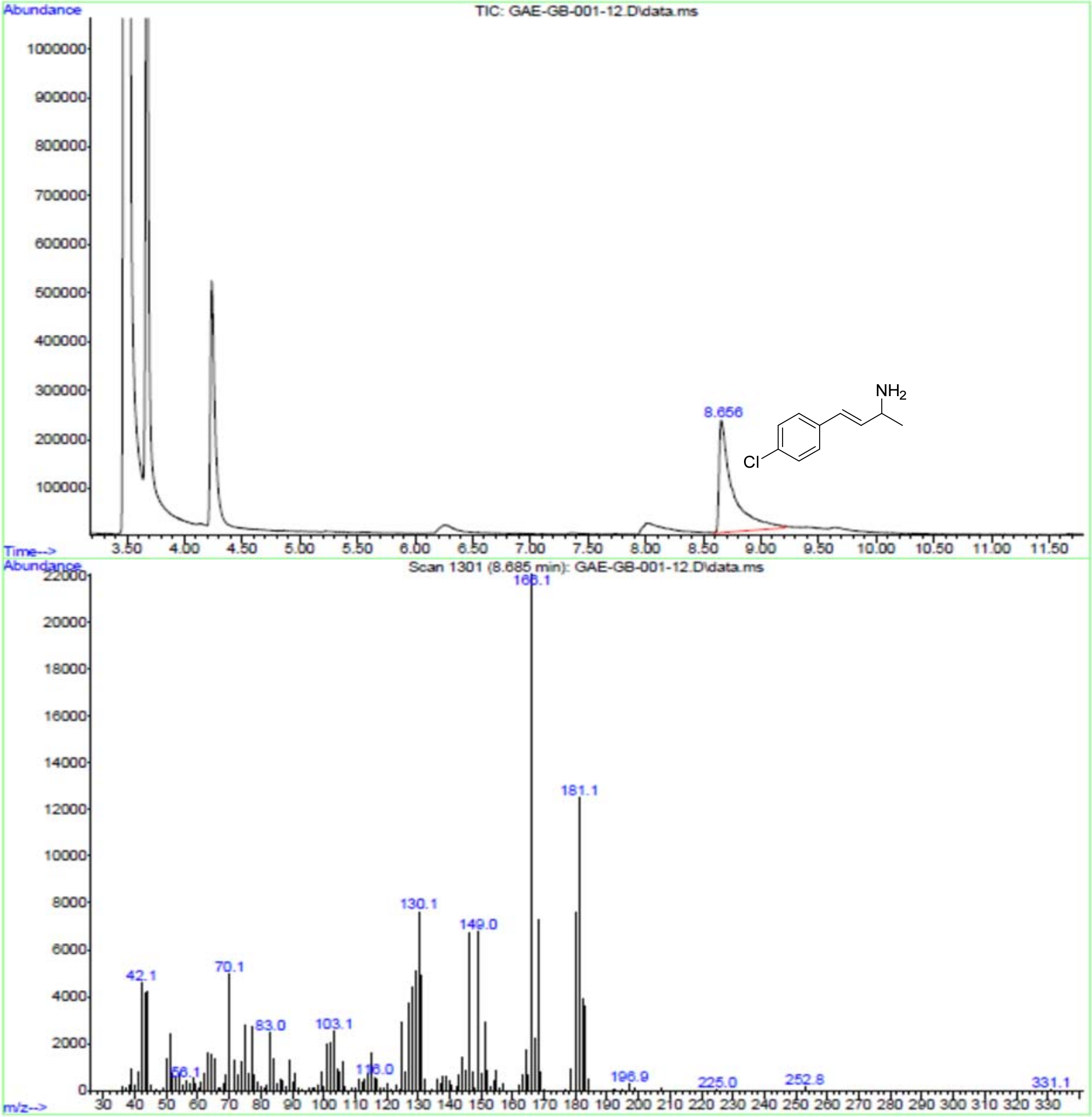

Figure S73. GC-MS chromatogram of amination of **2a** (10 mM) employing ArRmut11-ω-TA and (*R*)-1-phenylethyl amine (250 mM) as donor measured on HP-5MS column (5% phenylmethylsiloxane, 30 m x 0.20 mm x 0.25 µm, J&W Scientific, Agilent Technologies) using He as carrier gas. Injector temperature: 250 °C; split ratio: 90:1; Injection volume: 5 µL; Flow rate: 0.7 mL/min; Temperature program (standard method): 100 °C, hold time 0.5 min, 10 °C/min to 300 °C, hold time 0 min; EI mode, energy 70 eV, MS Source: 230 °C, MS Quadrupole: 150 °C.

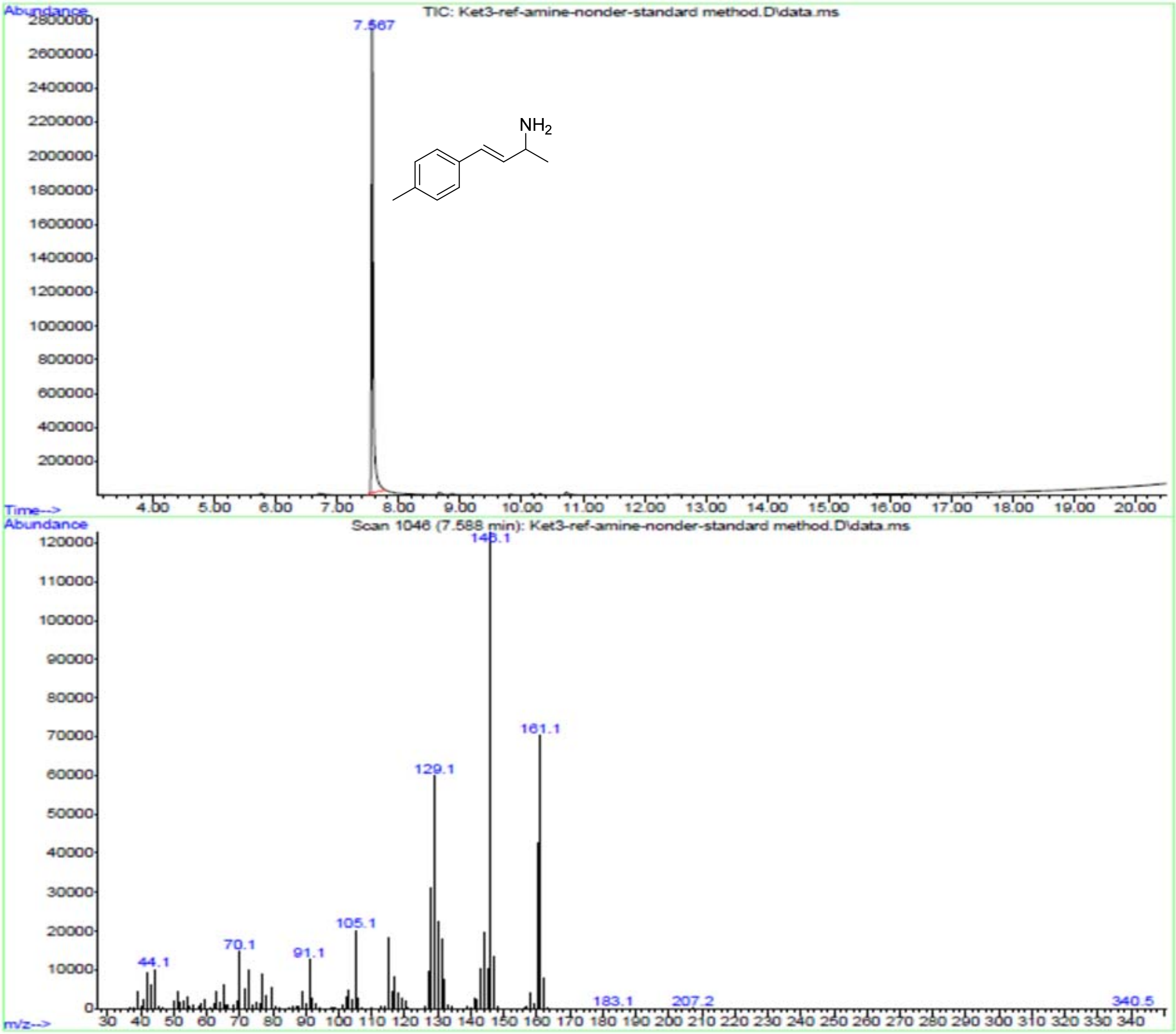

Figure S74. GC-MS chromatogram of **3c** measured on HP-5MS column (5% phenylmethylsiloxane, 30 m x 0.20 mm x 0.25 µm, J&W Scientific, Agilent Technologies) using He as carrier gas. Injector temperature: 250 °C; split ratio: 90:1; Injection volume: 5 µL; Flow rate: 0.7 mL/min; Temperature program (standard method): 100 °C, hold time 0.5 min, 10 °C/min to 300 °C, hold time 0 min; EI mode, energy 70 eV, MS Source: 230 °C, MS Quadrupole: 150 °C.

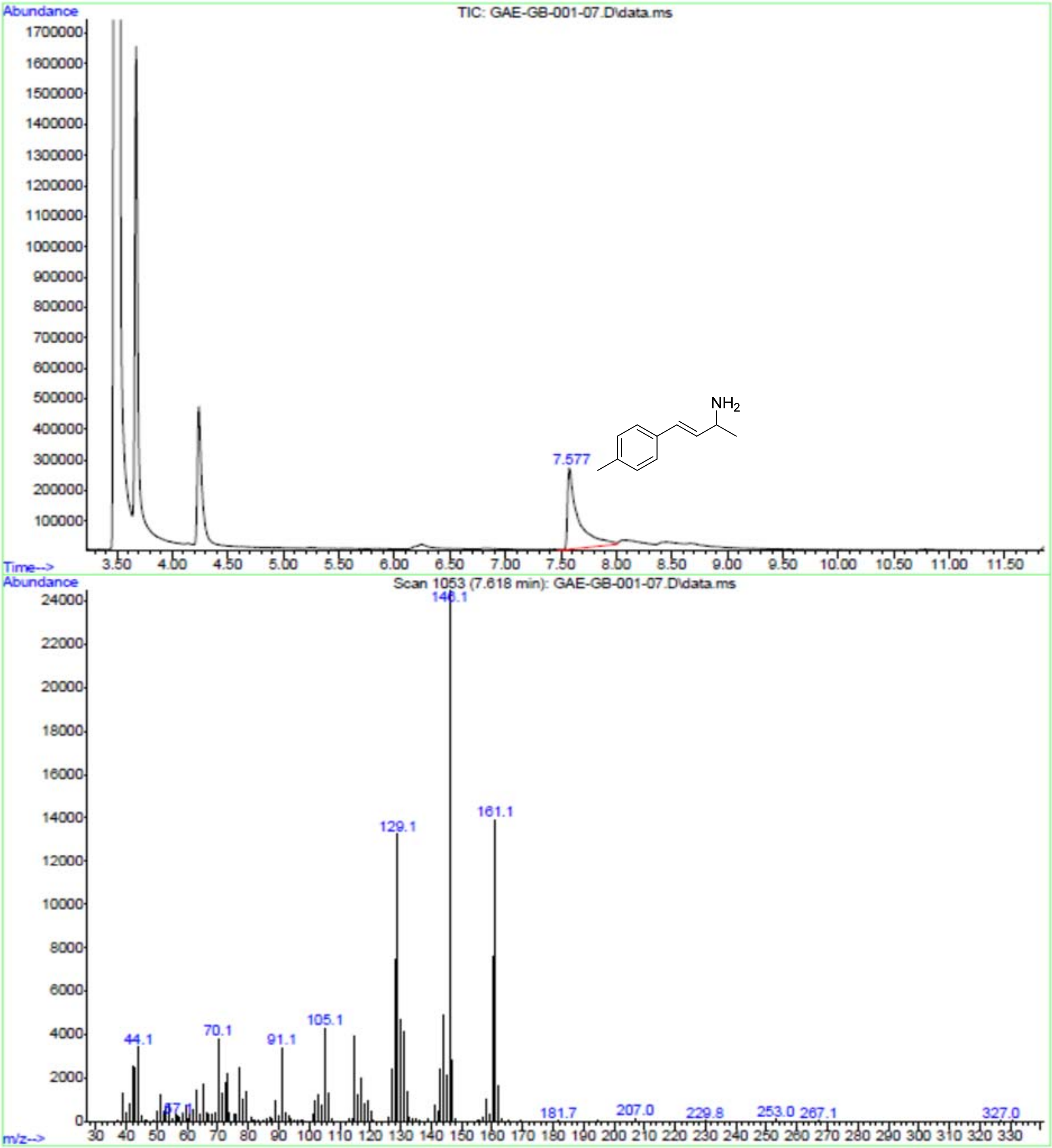

Figure S75. GC-MS chromatogram of amination of **3a** (10 mM) employing ArRmut11-ω-TA and (*R*)-1-phenylethyl amine (250 mM) as donor measured on HP-5MS column (5% phenylmethylsiloxane, 30 m x 0.20 mm x 0.25 µm, J&W Scientific, Agilent Technologies) using He as carrier gas. Injector temperature: 250 °C; split ratio: 90:1; Injection volume: 5 µL; Flow rate: 0.7 mL/min; Temperature program (standard method): 100 °C, hold time 0.5 min, 10 °C/min to 300 °C, hold time 0 min; EI mode, energy 70 eV, MS Source: 230 °C, MS Quadrupole: 150 °C.

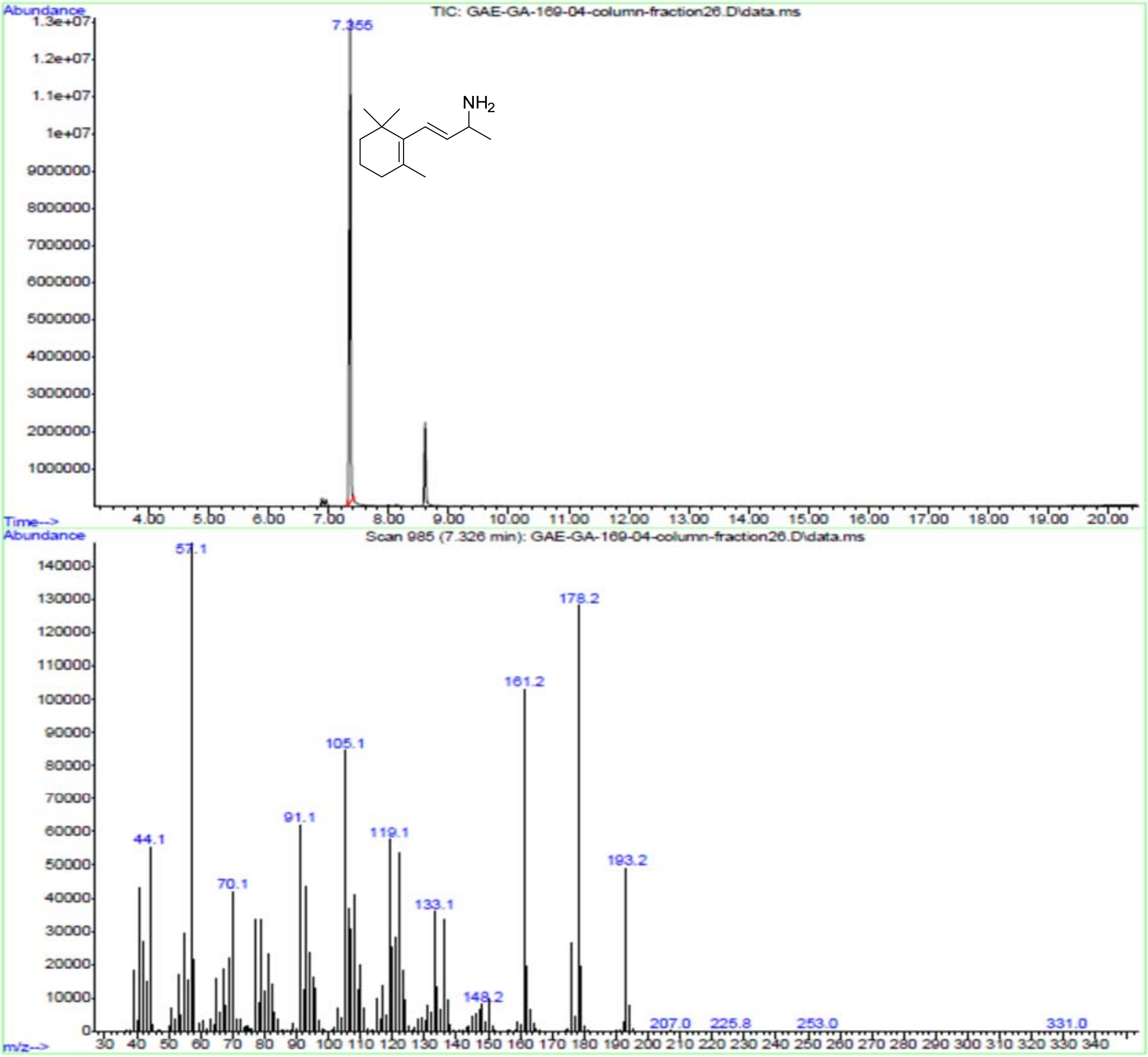

Figure S76. GC-MS chromatogram of **4c** measured on HP-5MS column (5% phenylmethylsiloxane, 30 m x 0.20 mm x 0.25 µm, J&W Scientific, Agilent Technologies) using He as carrier gas. Injector temperature: 250 °C; split ratio: 90:1; Injection volume: 5 µL; Flow rate: 0.7 mL/min; Temperature program (standard method): 100 °C, hold time 0.5 min, 10 °C/min to 300 °C, hold time 0 min; EI mode, energy 70 eV, MS Source: 230 °C, MS Quadrupole: 150 °C. Peak at 8.6 min is related to an impurity.

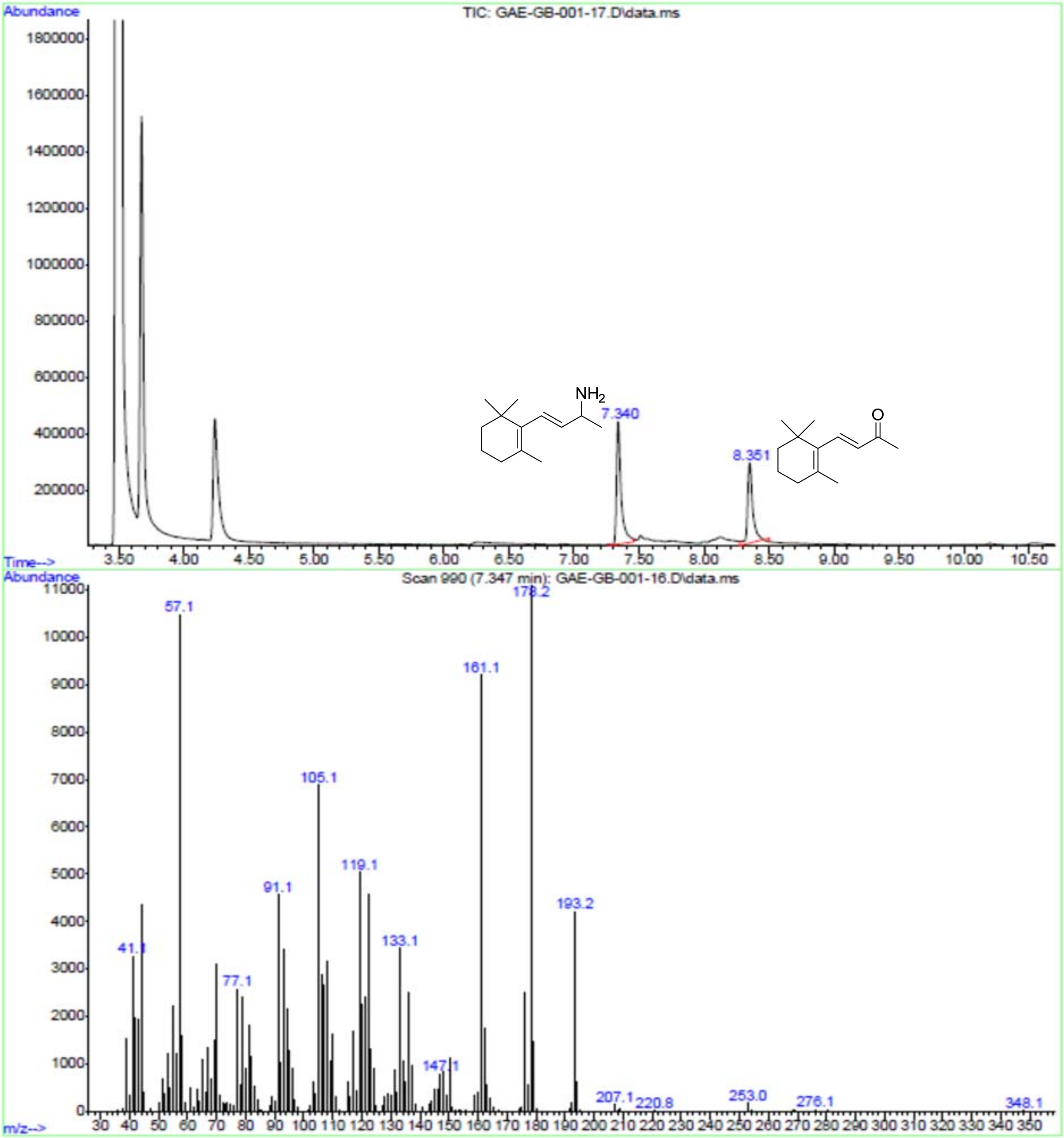

Figure S77. GC-MS chromatogram of amination of **4a** (10 mM) employing ArRmut11-ω-TA and (*R*)-1-phenylethyl amine (250 mM) as donor measured on HP-5MS column (5% phenylmethylsiloxane, 30 m x 0.20 mm x 0.25 µm, J&W Scientific, Agilent Technologies) using He as carrier gas. Injector temperature: 250 °C; split ratio: 90:1; Injection volume: 5 µL; Flow rate: 0.7 mL/min; Temperature program (standard method): 100 °C, hold time 0.5 min, 10 °C/min to 300 °C, hold time 0 min; EI mode, energy 70 eV, MS Source: 230 °C, MS Quadrupole: 150 °C.

4.6. GC-MS calibration

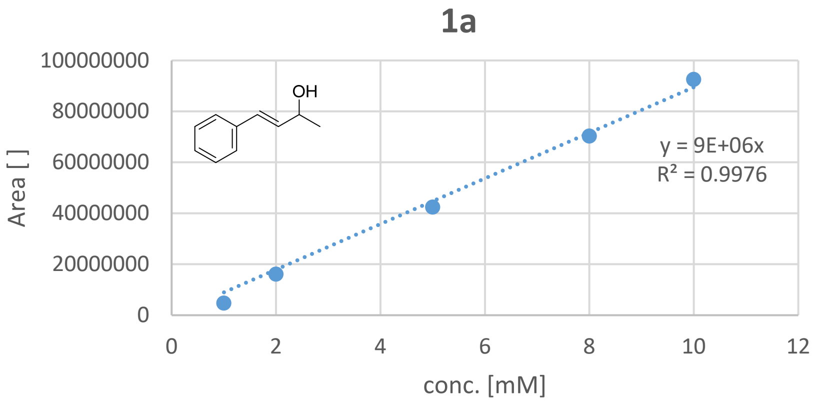

Figure S78. Calibration curve for **1a**.

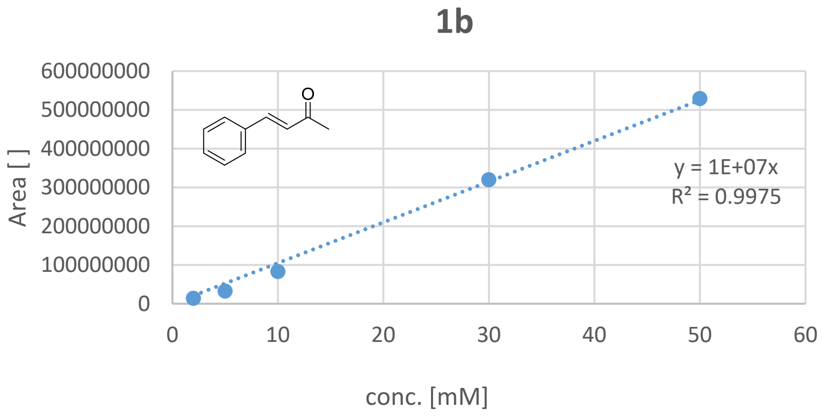

Figure S79. Calibration curve for **1b**.

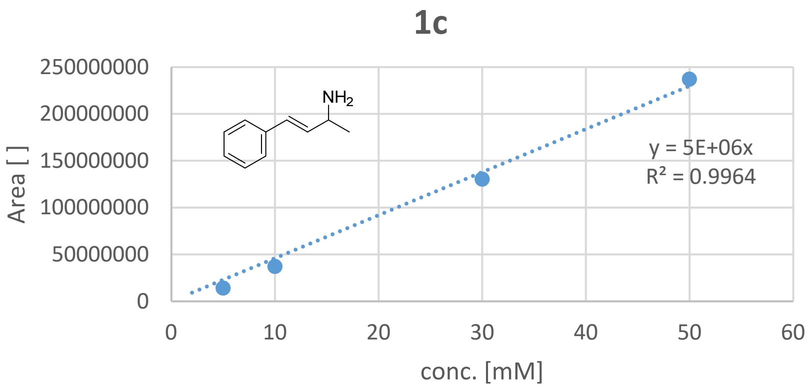

Figure S80. Calibration curve for **1c**.

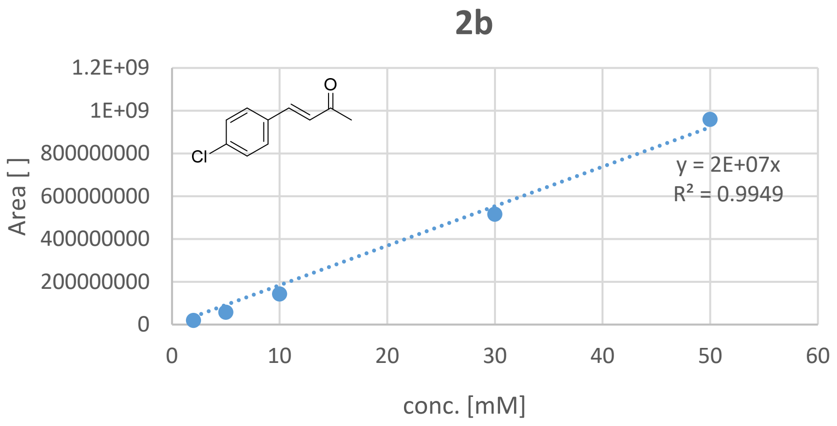

Figure S81. Calibration curve for **2b**.

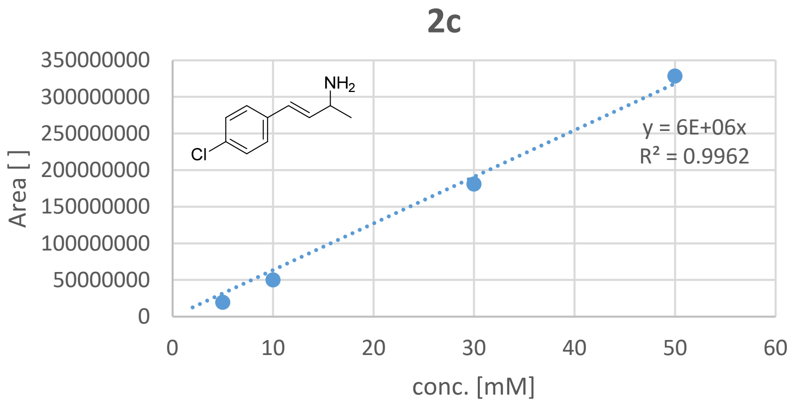

Figure S82. Calibration curve for **2c**.

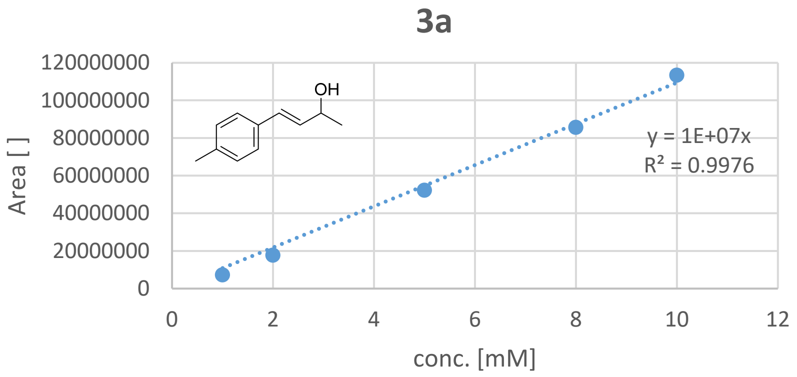

Figure S83. GC-MS calibration curve for **3a**.

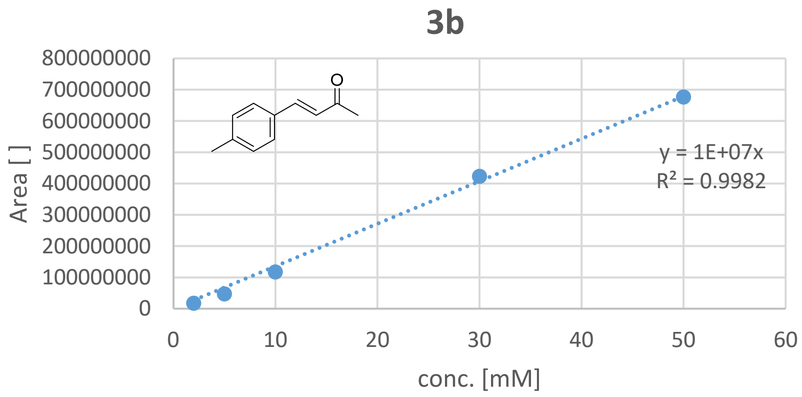

Figure S84. GC-MS calibration curve for **3b**.

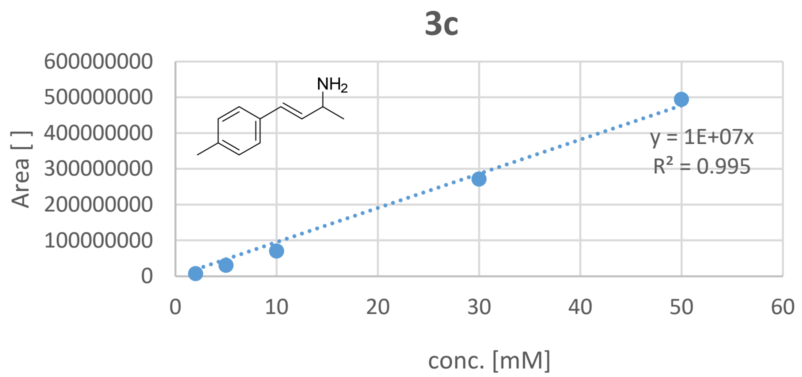

Figure S85. GC-MS calibration curve for **3c**

4.7. GC-FID calibration

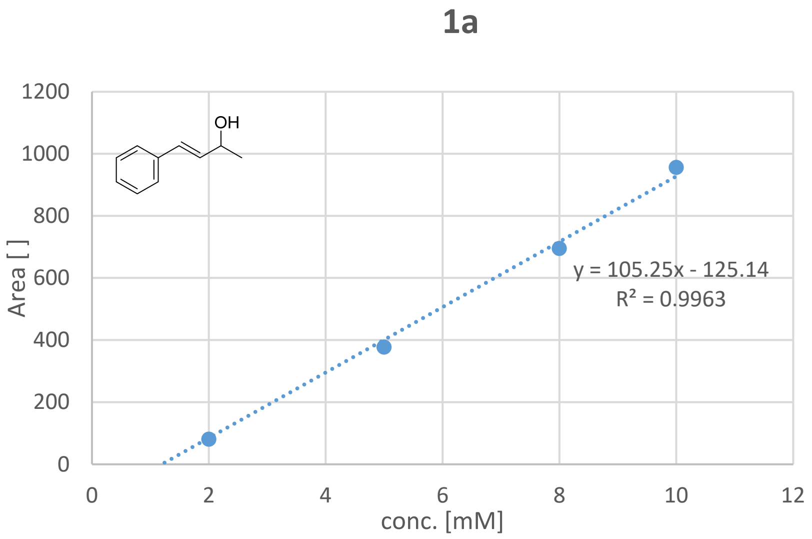

Figure S86. GC-FID calibration curve for **1a**.

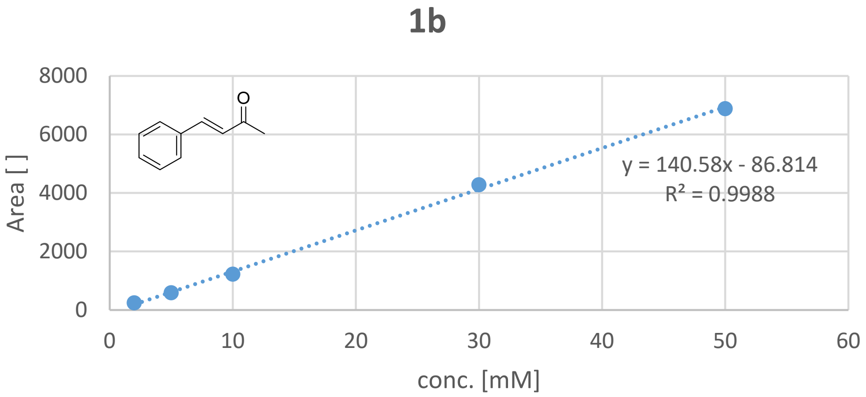

Figure S87. GC-FID calibration curve for **1b**.

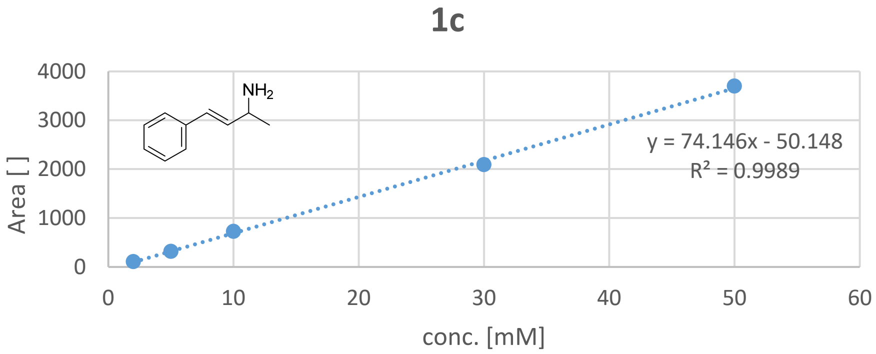

Figure S88. GC-FID calibration curve for **1c**.

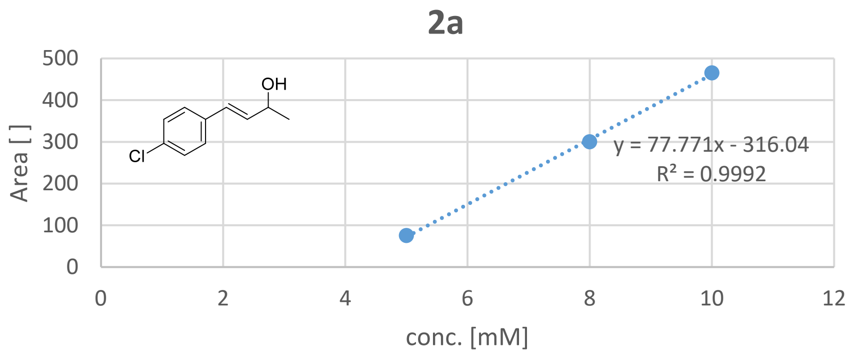

Figure S89. GC-FID calibration curve for **2a**.

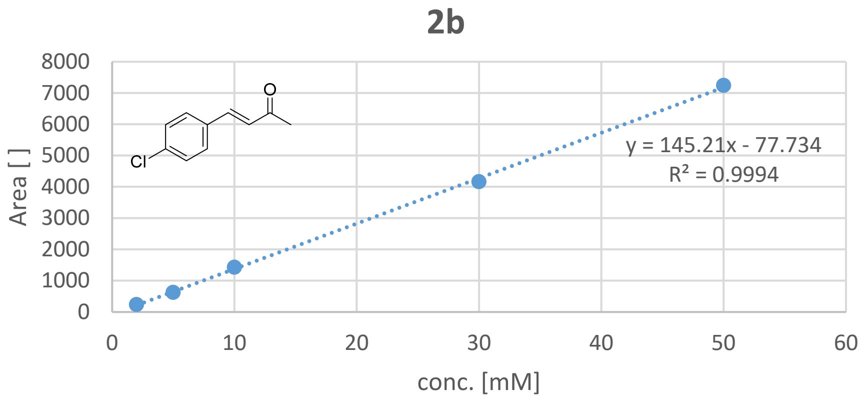

Figure S90. GC-FID calibration curve for **2b** measured on HP-5 column (30 m x 0.32 mm x 0.25 µm, J&W Scientific, Agilent Technologies) using He as carrier gas. Injector temperature: 250 °C; split ratio: 90:1; Injection volume: 5 µL; Flow rate: 0.7 mL/min; Temperature program (Standard Method): 100 °C, hold time 0.5 min, 10 °C/min to 300 °C, hold time 0 min.

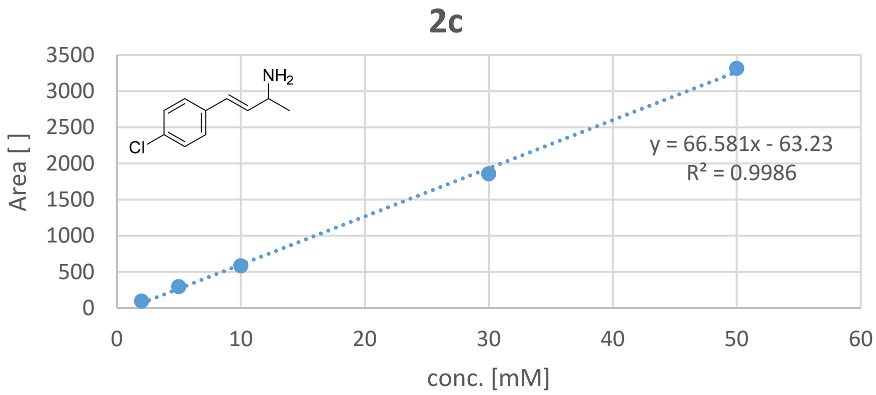

Figure S91. GC-FID calibration curve for **2c** measured on HP-5 column (30 m x 0.32 mm x 0.25 µm, J&W Scientific, Agilent Technologies) using He as carrier gas. Injector temperature: 250 °C; split ratio: 90:1; Injection volume: 5 µL; Flow rate: 0.7 mL/min; Temperature program (Standard Method): 100 °C, hold time 0.5 min, 10 °C/min to 300 °C, hold time 0 min.

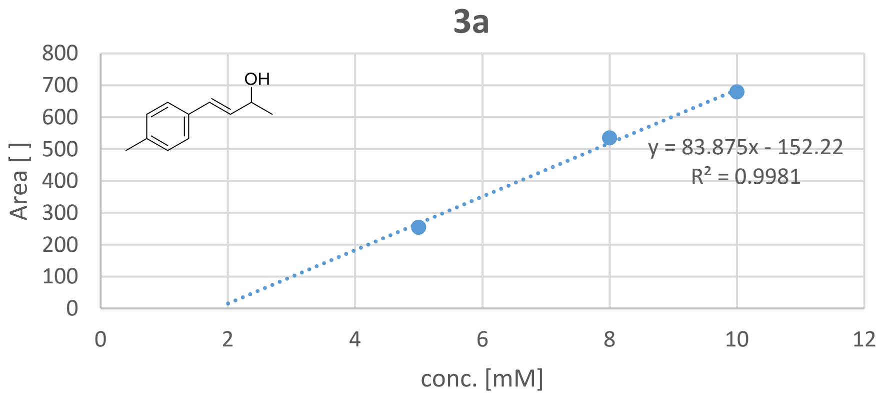

Figure S92. GC-FID calibration curve for **3a** measured on HP-5 column (30 m x 0.32 mm x 0.25 µm, J&W Scientific, Agilent Technologies) using He as carrier gas. Injector temperature: 250 °C; split ratio: 90:1; Injection volume: 5 µL; Flow rate: 0.7 mL/min; Temperature program (Standard Method): 100 °C, hold time 0.5 min, 10 °C/min to 300 °C, hold time 0 min.

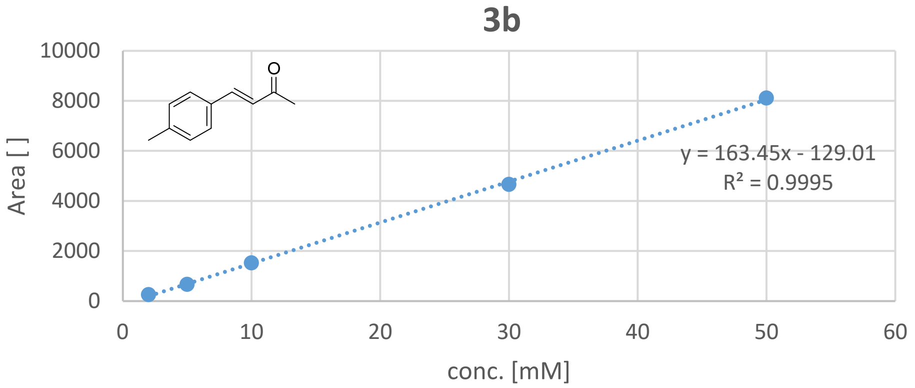

Figure S93. GC-FID calibration curve for **3b** measured on HP-5 column (30 m x 0.32 mm x 0.25 µm, J&W Scientific, Agilent Technologies) using He as carrier gas. Injector temperature: 250 °C; split ratio: 90:1; Injection volume: 5 µL; Flow rate: 0.7 mL/min; Temperature program (Standard Method): 100 °C, hold time 0.5 min, 10 °C/min to 300 °C, hold time 0 min.

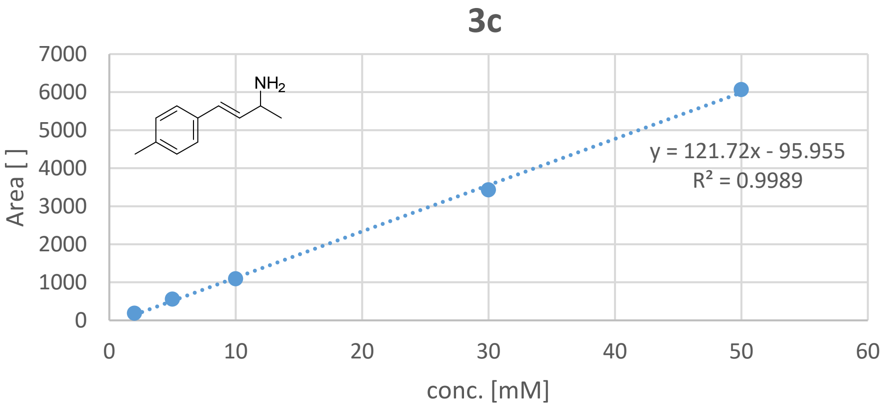

Figure S94. GC-FID calibration curve for **3c** measured on HP-5 column (30 m x 0.32 mm x 0.25 µm, J&W Scientific, Agilent Technologies) using He as carrier gas. Injector temperature: 250 °C; split ratio: 90:1; Injection volume: 5 µL; Flow rate: 0.7 mL/min; Temperature program (Standard Method): 100 °C, hold time 0.5 min, 10 °C/min to 300 °C, hold time 0 min.

5. References

[1] B. Meng, X. Huang, L. Wu, *Adv. Synth. Catal.* **2013**, *355*, 2637-2650.
[2] T. Y. Chaudhari, A. Hossian, M. K. Manna, R. Jana, *Org. Biomol. Chem.* **2015**, *13*, 4841-4845.
[3] Z. Yan, Y. Xu, W. Tian. *Tetrahedron Lett.* **2014**, *55*, 7186-7189.
[4] A. M. Decker, J. S. Partilla, M. H. Baumann, R. B. Rothman, B. E. Blough, *Med. Chem. Commun.* **2016**, *7*, 1657-1663.
[5] A. Alberola, J. M. Banez, L. Calvo, M. T. R. Rodriguez, M. C. Sanudo, *J. Heterocyclic Chem.* **1993**, *30*, 467-471.
[6] E. G. Klauber, N. Mittal, T. K. Shah, D. Seidel, *Org. Lett.* **2011**, *13*, 2464-2467.
[7] G. Buechi, J. C. Vederas, *J. Am. Chem. Soc.* **1972**, *94*, 9128-9132.
[8] F. Kienzle, *Helv. Chim. Acta.* **1973**, *56*, 1662-1671.
[9] A. R. Katritzky, D. Cheng, J. Li, *J. Org. Chem.* **1998**, *63*, 3438-3444.
[10] a) C. K. Savile, J. M. Janey, E. C, Mundorff, J. C. Moore, S. Tam, W. R. Javis, J. C. Colbeck, A. Krebber, F. J. Fleitz, J. Brands, P. N. Devine, G. W. Huisman, G. J. Hughes, *Science* **2010**, *329*, 305-309; b) F. G. Mutti, C. S. Fuchs, D. Pressnitz, J. H. Sattler, W. Kroutil, *Adv. Synth. Catal.* **2011**, *353*, 3227-3233.
[11] a) H. Yun, S. Lim, B.-K. Cho, B.-G. Kim, *Appl. Environ. Microbiol.* **2004**, *70*, 2529-2534; b) D. Koszelewski, D. Pressnitz, D. Clay, W. Kroutil, *Org. Lett.* **2009**, *11*, 4810-4812.
[12] F. G. Mutti, C. S. Fuchs, D. Pressnitz, J. H. Sattler, W. Kroutil, *Adv. Synth. Catal.* **2011**, *353*, 3227-3233.
[13] a) E. Park, M. Kim, J.-S. Shin, *Adv. Synth. Catal.* **2010**, *352*, 3391-3398; b) F. G. Mutti, C. S. Fuchs, D. Pressnitz, N. G. Turrini, J. H. Sattler, A. Lerchner, A. Skerra, W. Kroutil, *Eur. J. Org. Chem.* **2012**, *2012*, 1003-1007.
[14] M. Fuchs, K. Tauber, J. Sattler, H. Lechner, J. Pfeffer, W. Kroutil, K. Faber, *RSC Adv.* **2012**, *2*, 6262-6265.
[15] D. Pressnitz, C. S. Fuchs, J. H. Sattler, T. Knaus, P. Macheroux, F. G. Mutti, W. Kroutil, *ACS Catal.* **2013**, *3*, 555-559.
[16] R. C. Simon, F. Zepeck, W. Kroutil, *Chem. Eur. J.* **2013**, *19*, 2859-2865.
[17] a) R. L. Hanson, B. L. Davis, Y. Chen, S. L. Goldberg, W. L. Parker, T. P. Tully, M. A. Montana, R. N. Patel, *Adv. Synth. Catal.* **2008**, *350*, 1367-1375; b) D. Koszelewski, M. Göritzer, D. Clay, B. Seisser, W. Kroutil, *ChemCatChem* **2010**, *2*, 73-77.
[18] M. Höhne, S. Schätzle, H. Jochens, K. Robins, U. T. Bornscheuer, *Nat. Chem. Biol.* **2010**, *6*, 807-813.
[19] S. E. Payer, J. H. Schrittwieser, W. Kroutil, Eur. *J. Org. Chem.* **2017**, *2017*, 2553-2559.
[20] E.-S. Park, M. Kim, J.-S. Shin, *Appl. Microbiol. Biotechnol.* **2012**, *93*, 2425-2435.
[21] F. Steffen-Munsberg, C. Vickers, A. Thontowi, S. Schätzle, T. Tumlirsch, M. S. Humble, H. Land, P. Berglund, U. T. Bornscheuer, M. Höhne, *ChemCatChem*, **2013**, *5*, 150-153.
[22] F. Cabirol, A. Gohel, S. H. Oh, D. Smith, B. Wong, J. Lalonde, WO2011/159910 A2, Codexis Inc.
[23] J. Albarrán-Velo, I. Lavandera, V. Gotor-Fernández, *ChemBioChem* **2020**, *21*, 200-211.
[24] E. G. Klauber, N. Mittal, T. K. Shah, D. Seidel, *Org. Lett.* **2011**, *13*, 2464-2467.
[25] X. Chen, H. Zhou, K. Zhang, J. Li, H. Huang, *Org. Lett.* **2014**, *16*, 3912-3915.
[26] T. L. Liu, C.J. Wang, X. Zhang, *Angew. Chem.* **2013**, *125*, 8574-8577; *Angew. Chem. Int. Ed.* **2013**, *52*, 8416-8419.
[27] P. He, X. Liu, H. Zheng, W. Li, L. Lin, X. Feng, *Org. Lett.* **2012**, *14*, 5134-5137.
[28] F. Chen, Y. Zhang, L. Yu, S. Zhu, *Angew. Chem.* **2017**, *129*, 2054-2057; *Angew. Chem. Int. Ed.* **2017**, *56*, 2022-2025.
